# Associations between gut microbiota and sarcopenia or its defining parameters in older adults: A systematic review

**DOI:** 10.1002/jcsm.13569

**Published:** 2024-08-27

**Authors:** Laurence Lapauw, Aurélie Rutten, Jolan Dupont, Nadjia Amini, Laura Vercauteren, Muriel Derrien, Jeroen Raes, Evelien Gielen

**Affiliations:** ^1^ Department of Public Health and Primary Care, Division of Gerontology and Geriatrics KU Leuven Leuven Belgium; ^2^ Division of Gerontology and Geriatrics Zuyderland Medisch Centrum Sittard The Netherlands; ^3^ Division of Gerontology and Geriatrics University Hospitals Leuven Leuven Belgium; ^4^ Department of Microbiology, Immunology and Transplantation, Rega Institute KU Leuven Leuven Belgium; ^5^ VIB Center for Microbiology Leuven Belgium

**Keywords:** Gut microbiota, Muscle mass, Muscle strength, Older adults, Physical performance, Sarcopenia

## Abstract

Altered gut microbiota (GM) potentially contribute to development or worsening of sarcopenia through a gut‐muscle axis. This systematic review aims to compare GM between persons with sarcopenia or low sarcopenia‐defining parameters (muscle mass, strength, and physical performance) to those with preserved muscle status, as well as to clarify possible associations between sarcopenia (‐defining parameters) and relative abundance (RA) of GM‐taxa or GM‐(α‐ or β) diversity indices, in order to clarify whether there is robust evidence of the existence of a GM signature for sarcopenia. This systematic review was conducted according to the PRISMA‐reporting guideline and pre‐registered on PROSPERO (CRD42021259597). PubMed, Web of Science, Embase, ClinicalTrials.gov, and Cochrane library were searched until 20 July 2023. Included studies reported on GM and sarcopenia or its defining parameters. Observational studies were included with populations of mean age ≥50 years. Thirty‐two studies totalling 10 781 persons (58.56% ♀) were included. Thirteen studies defined sarcopenia as a construct. Nineteen studies reported at least one sarcopenia‐defining parameter (muscle mass, strength or physical performance). Studies found different GM‐taxa at multiple levels to be significantly associated with sarcopenia (*n* = 4/6), muscle mass (*n* = 13/14), strength (*n* = 7/9), and physical performance (*n* = 3/3); however, directions of associations were heterogeneous and also conflicting for specific GM‐taxa. Regarding β‐diversity, studies found GM of persons with sarcopenia, low muscle mass, or low strength to cluster differently compared with persons with preserved muscle status. α‐diversity was low in persons with sarcopenia or low muscle mass as compared with those with preserved muscle status, indicating low richness and diversity. In line with this, α‐diversity was significantly and positively associated with muscle mass (*n* = 3/4) and muscle strength (*n* = 2/3). All reported results were significant (*P* < 0.05). Persons with sarcopenia and low muscle parameters have less rich and diverse GM and can be separated from persons with preserved muscle mass and function based on GM‐composition. Sarcopenia and low muscle parameters are also associated with different GM‐taxa at multiple levels, but results were heterogeneous and no causal conclusions could be made due to the cross‐sectional design of the studies. This emphasizes the need for uniformly designed cross‐sectional and longitudinal trials with appropriate GM confounder control in large samples of persons with sarcopenia and clearly defined core outcome sets in order to further explore changes in GM‐taxa and to determine a sarcopenia‐specific GM‐signature.

## Introduction

According to the second definition of the European Working Group on Sarcopenia in older People (EWGSOP2), sarcopenia is a progressive and generalized skeletal muscle disorder (muscle failure), that is associated with an increased risk of adverse outcomes such as falls, fractures, disability, and mortality.^1^, ^2^ The EWGSOP2‐definition considers sarcopenia as primary when no other factor than the aging process is evident. If co‐morbidities influencing muscle are prevalent, sarcopenia is considered ‘secondary’.^3^ Sarcopenia affects 10–27% of persons >60 years, predisposing older adults to an increased risk of adverse health outcomes, for example, functional decline, falls, and fractures.^4^ Consequently, sarcopenia exerts a high burden on health care and results in substantial financial costs, which are predicted to further increase.^5^, ^6^ This warrants new, specific biomarkers for sarcopenia to support imaging techniques and performance tests currently applied for its diagnosis, eventually allowing timely initiation of treatment.

Recent studies in large populations indicate that gut microbiota (GM) composition changes with age^7^ and even in persons of the same age, is altered depending on health status.^8^ As such, gut dysbiosis is associated with age‐related conditions, for example, frailty, Alzheimer's, and Parkinson's disease.^9^, ^10^ GM may influence muscle physiology and metabolism by modulating systemic inflammation, energy production, and insulin sensitivity—the so‐called ‘gut‐muscle’ axis. Data from animal studies demonstrated associations between GM‐composition, low muscle mass, and sarcopenia.^11^, ^12^, ^13^ These studies suggest GM as a potential prognostic, diagnostic, or even modulatory factor as a potential future treatment option for sarcopenia.^14^

Nevertheless, some research gaps remain. First, before considering modulation of GM as a possible treatment for sarcopenia, the alterations in the GM landscape in sarcopenic subjects should be fully understood. Previously, psychiatric conditions and inflammatory bowel disease (IBD), the latter with a sarcopenia prevalence of 42%, have been associated with a specific GM‐signature.^15^, ^16^, ^17^ However, prior studies failed to unambiguously report on a specific GM‐signature related to sarcopenia or its defining parameters (low muscle mass, muscle strength, and physical performance).^14^

Second, although prior systematic reviews have been conducted regarding the association between GM and sarcopenia, muscle mass, muscle strength, or physical performance, these reviews mainly included animal studies and only to a lesser extent human studies.^14^, ^18^ Moreover, the populations of some of the studies included in these reviews were often relatively young, mean ages ranging between 30 ± 6 years to 45.12 ± 12.47 years, and one review investigated ‘muscle wasting’ in persons suffering from renal failure, liver cirrhosis, or cancer, but not ‘primary’ sarcopenia. Also, disentangling the effect of aging from other factors, such as diet or (age‐related) disease is difficult, which was insufficiently addressed in any of these two reviews. Additionally, none of these reviews systematically reported on diversity markers, which could also be investigated as potential biomarker for sarcopenia, rather than specific bacterial taxa.

Finally, research on the association between GM and sarcopenia or its defining parameters has gained interest, resulting in an increased number of published studies. This warrants a more updated state of the art literature overview.

Therefore, we performed a systematic review to summarize the most recent literature on associations between GM and sarcopenia (‐defining parameters), in studies conducted in older adults. This review also aims investigate GM‐composition in both primary and secondary sarcopenia. Finally, in this review, we aim to systematically investigate GM‐diversity markers rather than solely the relative abundance (RA) of bacterial taxa in order to investigate the existence of sarcopenia‐associated GM markers, potentially serving as a new GM‐derived biomarker for sarcopenia.

## Methods

A protocol was preregistered on PROSPERO (CRD:42021259597). This systematic review followed the PRISMA (Preferred Reporting Items for Systematic Reviews and Meta‐analyses) reporting guideline^19^ and aimed to address the research question, ‘What is the potential role of GM on sarcopenia or its defining parameters in older adults?’, based on Patient Intervention Comparator Outcome criteria (Table 1).

**Table 1:** Patient/population intervention comparator outcome (PICO) strategy for the research question of this systematic review

Patient/population	Persons with sarcopenia or decline in muscle mass, muscle strength or physical performance
Intervention	No intervention, only cross‐sectional design
Comparator	Persons without sarcopenia or decline min muscle mass, muscle strength or physical performance
Outcome	Difference in abundance of GM between groups or associations between GM and sarcopenia (−defining parameters)

### Search strategy

Four electronic databases (Embase, Cochrane Library, Web of Science, and MEDLINE through PubMed) and one registry (ClinicalTrials.gov) were searched from inception to 20 July 2023. The complete search string for each database can be found in Appendix S1. A combination of Boolean operators was used for this search comprising the Medical Subject Headings (MeSH) with related key words: ‘sarcopenia’, ‘muscle strength’, ‘muscle mass’, ‘physical performance’, and ‘gut microbiota’.

After deduplication in EndNote using a standardized approach, three independent reviewers (J.D., L.L., and A.R.) assessed titles and abstracts of references using the online Rayyan screening tool.^20^ When relevance could not be determined from the abstracts, full‐text papers were retrieved and reviewed. Any persisting disagreements were resolved by a fourth reviewer (EG). Citation searching was applied to identify additional studies.

### Study selection

Three independent reviewers (J.D., L.L., and A.R.) read the full texts of remaining references, selecting studies applying to the following, eligibility criteria: mean age ≥50 years (due to accelerated atrophy of skeletal muscle from the age of 50 years^21^, ^22^), in a community‐dwelling, hospitalized, or residential setting. Eligible studies reported on sarcopenia or its defining parameters, on faecal sample collection and on methods of GM‐analysis, having a cross‐sectional, case–control or cohort design, and absence of underlying neurological or neurodegenerative conditions. Intervention trials, animal studies, in vitro studies, conference proceedings, and case studies were excluded from our systematic review.

### Data extraction

Data extracted using a standardized form, include author name, journal, study set‐up, population, socio‐demographic data (country, age range or mean age, community‐setting, number of included participants, ratio of men and women, co‐morbidities), definition of sarcopenia, prevalence of sarcopenia, sarcopenia‐defining parameters and tools to measure these, GM‐analysis methodology (e.g., 16S rRNA gene amplicon sequencing and shotgun whole metagenome sequencing), RA of bacterial taxa at multiple levels, GM‐diversity markers (α‐ and β‐diversity), confounders (e.g., intake of supplements or medications that influence the muscle or GM, such as antibiotics, corticoid medications and pre‐, pro‐, or synbiotics, and known microbiota confounders being diet, stool consistency, transit time, and inflammation) and the main results of included studies. Both non‐significant and significant findings were extracted from included studies. Due to the large quantity of findings, this review emphasizes significant results (*P*‐value < 0.05), but a complete overview of the non‐significant associations and differences in GM abundance is given in the appendices. Strength of reported correlations between GM and sarcopenia (−defining parameters) was considered weak (*r* < 0.3), fair (0.3 ≥ *r* < 0.6), moderate (0.6 ≥ *r* < 0.8), or strong (*r* ≤ 0.8).^23^

### Types of outcome measurements

Primary outcomes of interest can be categorized in two groups: sarcopenia‐associated outcomes and gut bacterial‐associated outcomes:

- Sarcopenia‐associated: the construct of sarcopenia according to different expert group definitions, prevalence of sarcopenia, measures of low muscle mass, muscle strength, and physical performance.
- GM‐associated: bacterial RA at multiple taxonomic (phylum, class, order, family, genus, and species) levels, *Firmicutes/Bacteroidetes* (F/B) ratio, markers of gut bacterial diversity, such as α‐ (within sample) diversity/richness indices (Chao1, Shannon, Amplicon Sequence Variants (ASV), Simpson, ACE, Pielou's evenness, and Faith's phylogenetic diversity indices), β‐(between samples) diversity (Bray–Curtis and (un)weighted UniFrac distance) and enterotypes.^24^, ^25^

### Quality assessment

Two reviewers (L.L. and A.R.) independently assessed the quality of the included studies using the adapted version of the Newcastle Ottawa Scale (NOS) for cohort (8 items, maximum score of 9 points), case–control (8 items, maximum score 9 points), and cross‐sectional studies (6 items, maximum score 7 points). When agreement was not met upon, a third reviewer (E.G.) was consulted. A higher score reflects higher quality, and an overview is given in Appendix S7.

## Results

### Characteristics of included studies

**Figure 1:**
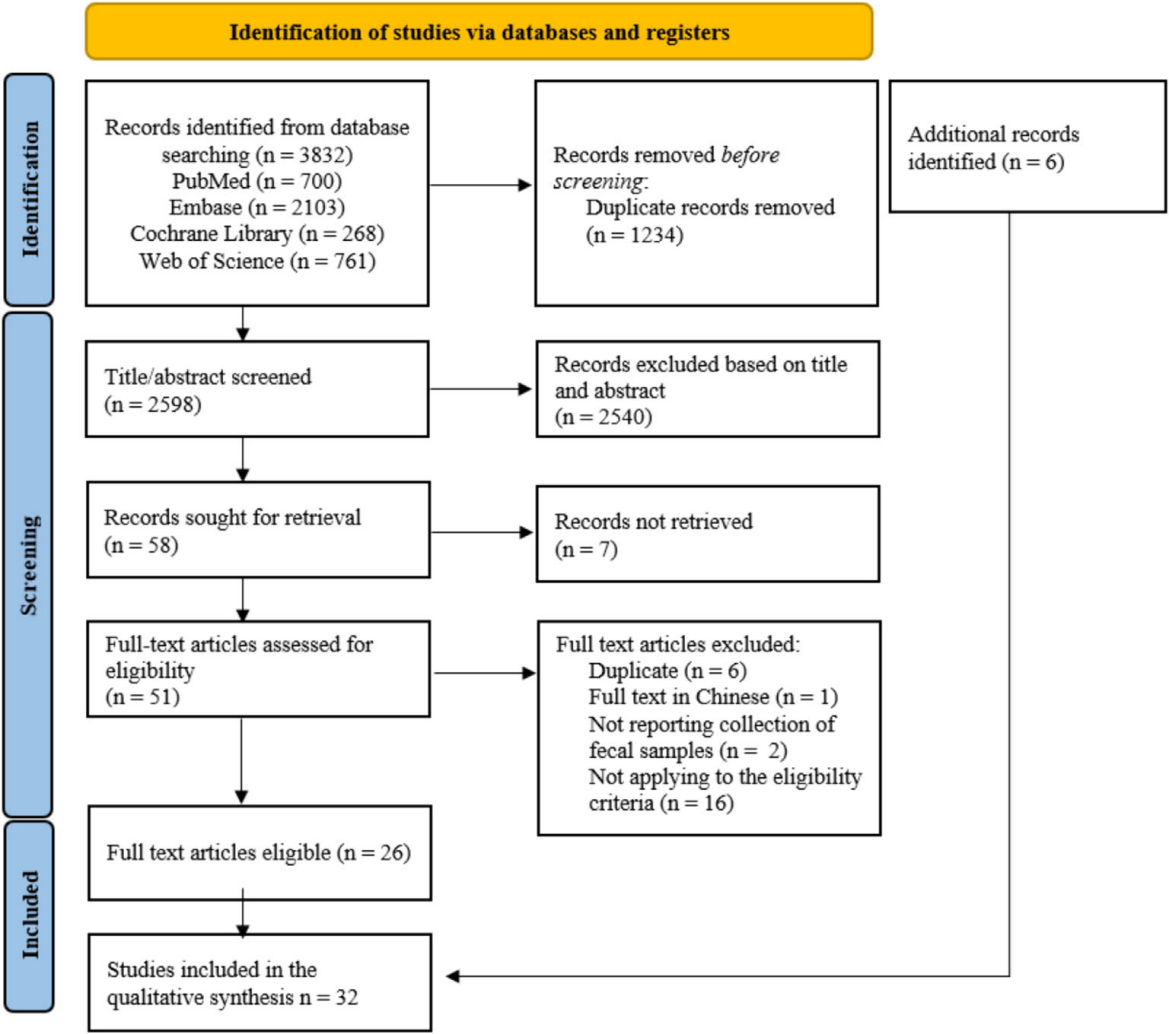
Preferred reporting items for systematic reviews.^21^

The electronic search generated 3832 records. Finally, 32 studies published between 2012 and 2023 were included in the qualitative analysis (Figure 1). In total, 10 781 persons were included, study sample sizes ranged between 17 and 5196 participants, and mean ages ranged between 50 and 85 years. Women were slightly more represented in the studies (58.56%; *n* = 6313). Twenty studies had a cross‐sectional design,^12^, ^26^, ^27^, ^28^, ^29^, ^30^, ^31^, ^32^, ^33^, ^34^, ^35^, ^36^, ^37^, ^38^, ^39^, ^40^, ^41^, ^42^, ^43^, ^44^ six were case–control studies,^13^, ^45^, ^46^, ^47^, ^48^, ^49^ and six were cohort studies, as based on their size.^50^, ^51^, ^52^, ^53^, ^54^, ^55^ Fifteen studies were conducted in Caucasian populations, 15 were conducted in Asian populations, one study included Asian and Caucasian participants, and one study included participants with Caucasian, Surinamese, North, and West African ethnicity. Twenty‐six studies comprised a community‐dwelling population, three studies included a hospitalized population, and one study included persons in long‐term residential care. Four studies did not clearly state upon the residency of the participants. One study included a mixed population regarding living arrangements.

### Assessed domains of sarcopenia

Multiple tools were used to assess sarcopenia‐defining parameters across included studies. Muscle mass was assessed using computed tomography (CT) (*n* = 2), bio‐electrical impedance analysis (BIA) (*n* = 14), dual X‐ray absorptiometry (DXA) (*n* = 9), and anthropometry (*n* = 4). Two studies did not clearly state upon the tool used to assess muscle mass. Muscle strength was assessed using a hand‐held dynamometer (21 studies), the chair stand test (CST) (*n* = 1), leg press (*n* = 1), lateral press (*n* = 1), and bench press (*n* = 1 study). Physical performance was assessed using the Short Physical Performance Battery (SPPB) (*n* = 5), gait speed tests (*n* = 8; e.g., 6‐m gait speed test, the 400 m walking test, the 6 min gait speed test), and the Timed‐Up and Go test (TUG) (*n* = 2). A detailed overview is given in Appendix S2.

### Assessment of gut microbiota

Twenty‐eight studies reported on 16S rRNA gene amplicon sequencing methods for GM‐analyses. Three studies applied shotgun whole genome sequencing (either deep with 10 million reads/sample^48^, ^53^ or shallow with 2–3 million reads/sample^39^). Another study applied Precision Microbiome Profiling.^55^ This latter technique performs a quantitative polymerase chain reaction (qPCR) on an open array.^56^ In shotgun whole genome sequencing, quantified and qualified DNA is fragmented to construct a DNA library, which is afterwards sequenced to identify the GM present and to gain insight in their function.^57^

**Figure 2:**
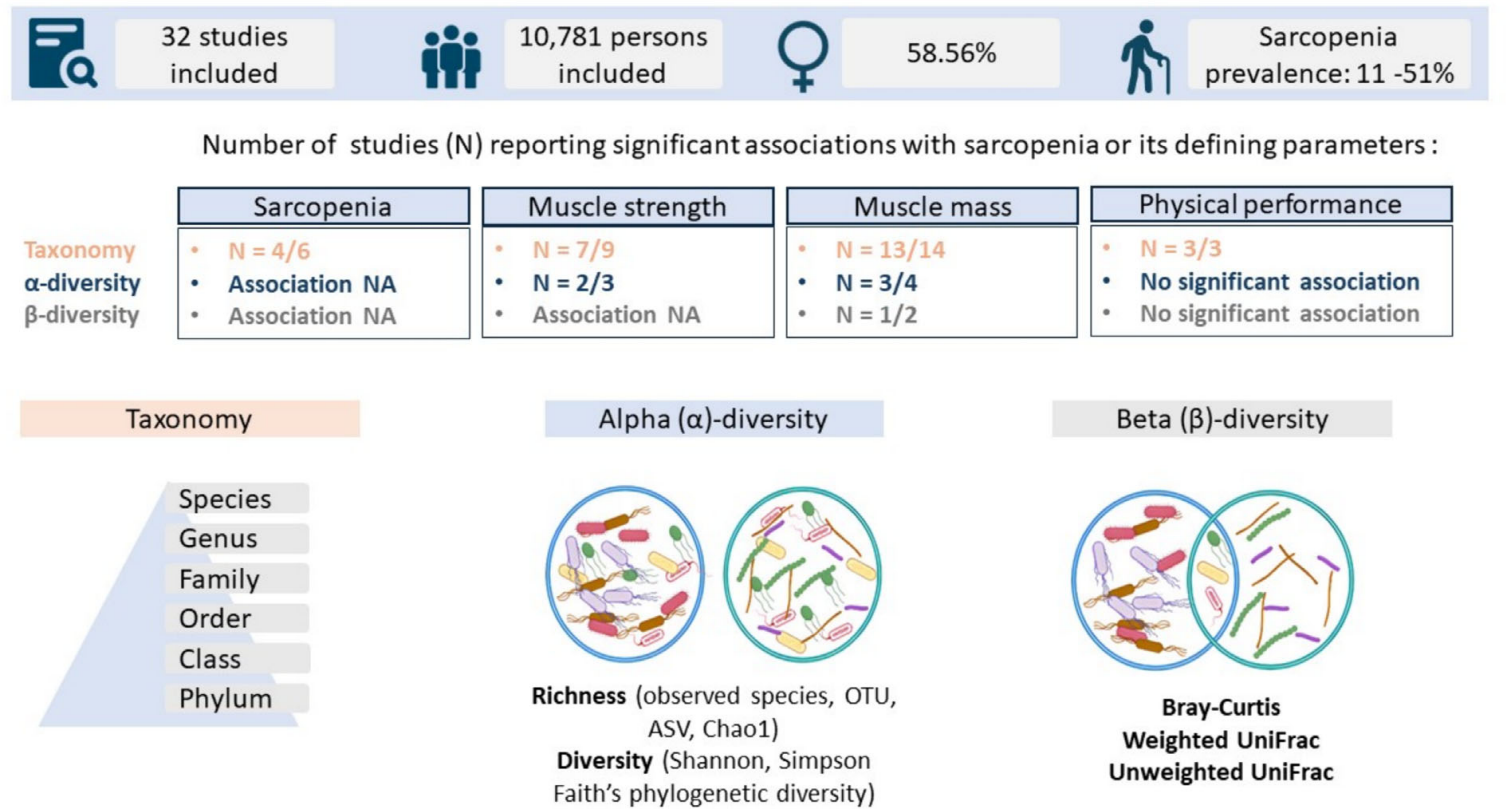
Overview on the main findings regarding associations between sarcopenia and GM‐related outcomes assessed in this systematic review. ASV, amplicon sequencing variants; GM, gut microbiota; NA, not assessed; OTU, operational taxonomic units.

Twelve of 32 studies corrected for at least one of the following confounders in their analyses: age, sex, body mass index (BMI), height, weight, waistline, ethnicity, community setting, Mini‐Nutritional Assessment score, physical activity, smoking status, ethyl intake, dietary intake, Bristol stool score, batch effects, chronic co‐morbidity, ‘medications potentially affecting the gut or muscle’, and liver test such as total bilirubin, aspartate aminotransferase (AST), alanine transaminase (ALT) and AST/ALT ratio. In 22/32 studies ‘recent intake’ of antibiotics, varying between 2 days and 6 months preceding study inclusion was an exclusion criterion, whereas one study specifically stated upon antibiotics intake in participants. The remaining nine studies did not state upon antibiotics intake, neither as an exclusion criterium. In respectively three, six, and one of 32 studies, intake of prebiotics, probiotics, and synbiotics was an exclusion criterium. Remaining studies did not state upon exclusion of pre‐, pro, or synbiotics. A detailed overview of the study characteristics is given in Appendix S2. An overview of the main sarcopenia and GM‐related outcomes is given in Figure 2.

### Quality assessment

Of the 32 studies, 12 were of low quality (NOS: 0–3 points), and the 20 remaining studies were of moderate quality (NOS: 4–6 points), whereas none of the studies were of high quality (NOS ≥ 7 points). Low quality of studies was mainly attributable to uncertain representativeness of the sample, low comparability, inadequate follow‐up, and unclear representativeness of the included population. A detailed overview is given in Appendix S7.

### Sarcopenia‐associated outcomes: Sarcopenia as a construct

Thirteen studies reported on the construct of sarcopenia. Seven studies defined sarcopenia according to the second definition of the Asian Working Group on Sarcopenia (AWGS2).^13^, ^42^, ^43^, ^45^, ^48^, ^53^, ^54^ The other studies defined sarcopenia according to the first or the revised definition of the European Working Group on Sarcopenia in Older People, respectively, EWGSOP1^12^, ^39^ and EWGSOP2,^35^, ^49^ the Foundation of National Health Institutes (FNIH)^46^ and the Sarcopenia and Physical fRailty IN older people: multi‐componenT treatment strategies (SPRINTT) project.^38^ The prevalence of sarcopenia ranged between 11.05% and 51.43%.

#### Relative abundance of gut microbiota taxa at multiple levels and markers of gut microbiota diversity

Thirteen studies reported on changes in GM‐composition at all taxonomic levels in persons with sarcopenia compared with persons without sarcopenia.

α‐diversity indices were investigated in 11 studies^12^, ^13^, ^38^, ^39^, ^42^, ^43^, ^45^, ^46^, ^48^, ^49^, ^53^, ^54^ of which six reported significant differences comparing between persons with and without sarcopenia.^12^, ^13^, ^42^, ^43^, ^46^, ^49^ Two and four studies found respectively that one (Chao1)^46^, ^49^ and multiple α‐diversity indices^12^, ^13^, ^42^, ^43^ were significantly lower in persons with sarcopenia. One study reported increased F/B ratio (descriptive composition value that is usually positively associated with α‐diversity) in persons with sarcopenia, but significance was unclear.^48^ Ten studies reported on β‐diversity,^12^, ^13^, ^39^, ^42^, ^43^, ^45^, ^46^, ^49^, ^53^, ^54^ and six found GM of persons with sarcopenia to cluster significantly different from persons without sarcopenia.^13^, ^42^, ^43^, ^45^, ^53^, ^54^ Appendix S3a,b gives an overview of respectively significantly and non‐significantly^13^, ^35^, ^39^, ^42^, ^43^, ^45^, ^49^, ^53^, ^54^ altered GM RA.

#### Associations between sarcopenia as a construct and respectively gut microbiota taxa and gut microbiota diversity markers

Six studies^35^, ^45^, ^48^, ^49^, ^53^, ^54^ reported on associations between GM and sarcopenia, of which all but two^35^, ^49^ withheld significant associations. Table 2 and Table S1 give an overview on respectively significant and non‐significant associations.^35^, ^45^, ^49^, ^54^ No studies have investigated associations between sarcopenia and diversity markers. The strength of reported correlations varied from poor (*r* < 0.3) to fair (0.3 ≤ *r* < 0.6).^23^

**Table 2:** Associations between the construct sarcopenia and multiple levels of GM taxa

Author, country, year	Sample size (*N*), sarcopenia definition, prevalence	Positive associations	Negative associations	Adjustment for confounders
Lee et al., South‐Korea, 2022^45^	*N* = 60, AWGS2, 45.00%	Genus: *Anaerotruncus* (*r* = 0.351) Species: *Phascolarctobacterium sp*. (*r* = 0.329)	Genus: *Prevotella* (*r* = −0.297) Species: *Prevotella copri* (*r* = −0.305)	Not adjusted
^c^Wu et al., Turkey, 2022^59^	*N* = 192, EWGSOP2, 45.83%	Genus: *Coprococcus*	Family: *Lachnospiraceae*	Not adjusted
Wang et al., China, 2022^53^	*N* = 1417, AWGS2, 11.05%	Genus: *Lawsonibacter* (β: 0.445) *Coprococcus* (β: 0.835) Species: *Desulfovibrio piger* ^a^ (β: 1.310) *Clostridium symbiosum* ^a^ (β: 0.729) *Hungatella effluvii* ^a^ (β: 0.701) *Bacteroides fluxus* ^a^ (β: 0.654) *Absiella innocuum* ^a^ (β: 0.651) *Coprobacter secundus* ^a^ (β: 0.597) *Clostridium citroniae* ^a^ (β: 0.513)		Adjusted for age, sex, BMI, smoking status and alcohol intake, fracture history, physical activity, frequency of dietary intake of meat/eggs, dairy products and vegetables
Lee et al., Taiwan, 2023^54^	*N* = 89, AWGS2, 32.58%	Genus: *Dialister* (OR: 6.823; 95% CI: [1.978–23.529]) *Ruminococcus 2* (OR: 4.590; 95% CI: [1.148–18.357]) *Anaerostipes* (OR: 4.640; 95% CI: [1.395–15.431]) *Megasphaera* ^b^ (OR: 2.931; 95% CI: [1.043–8.293])		Not adjusted
Wang et al., China, 2023^48^	*N* = 100, EWGSOP2, 50%	Species*: P. copri* GCF00157935 (β: 0.126)	Genus: *Atopium* (β: −0.019) Species: *Bifidobacterium longum* unclassified (β: −0.148)	Not adjusted

AWGS2, second gathering of the Asian Working Group on Sarcopenia; BMI, body mass index; CI, confidence interval; EWGSOP2, second gathering of the European Working Group on Sarcopenia in Older People (EWGSOP2); GM, gut microbiota; NA, not reported; NS, non‐significant; OR, odds ratio; *r*, correlation coefficient; β, regression coefficient.

^a^ Associated with sarcopenia severity.

^b^ Association became non‐significant after adjustment for multiple confounders.

^c^ Not clearly stated whether correlation was significant.

Specifically, two studies found associations between sarcopenia and the species *Prevotella copri* , but the results were inconsistent. As such Lee et al. found a fair negative correlation with sarcopenia, indicating that abundance of this species decreases the risk of sarcopenia.^45^ Contrarily, Wang et al. found this species to be positively associated with sarcopenia, indicating that its presence would increase the risk of sarcopenia.^48^ In the latter study *Prevotella copri* had a sensitivity of 4.1% and a specificity of 98.0% [area under the curve (AUC): 0.372; 95% CI: 0.261–0.484] to predict sarcopenia in older women,^48^ whereas *Bifidobacterium longum* appeared to be a better classifier of sarcopenia (sensitivity of 53.1%; specificity of 74.0%; AUC of 0.647, 95% CI: 0.539–0.756). Also, one study investigated a model to distinguish persons with and without sarcopenia by plotting a receiver operating curve (ROC) based on the presence the *Lachnospiraceae* family, the *Coprococcus* genus, and the *Prevotella/Bacteroides* ratio.^49^ The area under the curve (AUC) was 0.61, moderately distinguishing between persons with and without sarcopenia. However, sensitivity and specificity, nor specific correlations were reported.^49^ A final study found *Desulfovibrio piger* as a good classifier for sarcopenia (AUC 0.852; specificity nor sensitivity were reported). This study also found multiple species to be associated with sarcopenia severity, independent of age, sex, BMI, smoking, ethyl intake, fracture history, physical activity, frequency of eggs/meat, and dairy product intake.^53^

### Sarcopenia‐associated outcomes: Sarcopenia‐defining parameters (muscle mass, muscle strength and physical performance)

#### Muscle mass

##### Relative abundance of gut microbiota taxa at multiple levels and gut microbiota diversity markers

Nine studies^12^, ^26^, ^27^, ^31^, ^32^, ^33^, ^36^, ^41^, ^44^ investigated potentially altered RA of GM in persons with low compared with preserved muscle mass, although in two of these studies,^26^, ^36^ the difference between low and preserved muscle mass was borderline non‐significant. Eight studies found significantly altered bacterial RA in persons with low muscle mass. Significant, but heterogeneous, alterations were reported at all taxonomic levels. Appendix S4a,b gives an overview on respectively significantly and non‐significantly^12^, ^26^, ^27^, ^31^, ^32^, ^33^, ^36^, ^41^ altered GM comparing between persons with low and preserved muscle mass.

Of the seven studies investigating α‐diversity,^12^, ^27^, ^31^, ^32^, ^36^, ^41^, ^44^ four reported significant decreased α‐diversity indices, being Shannon (*n* = 2), Chao1 (*n* = 2), observed ASV/Richness (*n* = 3), Faith's phylogenetic (*n* = 1), and Simpson (*n* = 1) indices.^12^, ^31^, ^33^, ^41^ These indices were significantly decreased in persons with low muscle mass, indicating that their GM is less diverse/rich. However, in one study, these findings became non‐significant for Shannon and richness indices after adjustment for age, sex, BMI, and ethnicity.^31^ Also, the F/B ratio was significantly decreased in persons with low muscle mass in three studies.^12^, ^33^, ^41^ Of the six studies investigating β‐diversity,^12^, ^27^, ^31^, ^36^, ^41^, ^44^ three found persons with low muscle mass to cluster in separate groups compared with those with preserved muscle mass based on their GM‐composition, independent of age.^12^, ^27^, ^31^ Again, in the study by Houttu et al., significant clustering of GM between persons with low and preserved muscle mass became non‐significant after adjustment for age, sex, BMI, and ethnicity.^31^

##### Associations between muscle mass and gut microbiota taxa and gut microbiota diversity markers

Of the 14 studies^12^, ^13^, ^31^, ^32^, ^33^, ^37^, ^42^, ^44^, ^46^, ^47^, ^48^, ^50^, ^52^, ^55^ reporting associations between muscle mass and GM, one did not clearly state upon significance of the reported associations.^52^ Table 3 gives an overview on significant associations, whereas Table S2 gives an overview on non‐significant associations.^12^, ^13^, ^31^, ^32^, ^33^, ^37^, ^42^, ^44^, ^46^, ^47^, ^48^, ^50^, ^52^, ^55^ In studies reporting correlations, coefficients varied between weak (*r* < 0.3) and moderate (0.6 ≥ *r* < 0.8). If no correlation coefficients were reported, direction of correlations were derived.

**Table 3:** Associations between muscle mass and multiple levels of GM taxa and GM diversity markers

Authors, country, year	Sample size (*N*), muscle mass measure	GM‐taxa		Diversity markers	Adjustment for confounders
		Positive association	Negative association		
Claesson et al., Ireland, 2012^30^	*N* = 178, CC	NA	NA	α‐diversity: NA β‐diversity: Significantly associated with calf circumference in long‐term residence stay persons; at all four residence locations	Age, sex and community‐setting
Akashi et al., Japan, 2019^50^	*N* = 127, stTPA	No significant associations	Species: *Clostridium perfringens* (*r* = −0.15)	α‐diversity: NA β‐diversity: NA	Not adjusted
Dillon et al., USA, 2020^47^	*N* = 36, ALM, LBM	Genus: *Coprococcus* (β_HIV+, LBM_: 1.301) *Catenibacterium* (β_ALM, HIV−_: 0.486; β_LBM_: 0.821) *Butyrivibrio* (β_HIV−, ALM_: 2.16; β_HIV−, LBM_: 3.61)	Phylum: *Proteobacteria* (β_ALM, HIV+_: −0.080) Family: *Bacteroidaceae* (β_ALM, HIV+_: −0.040) Genus: *Subdoligranulum* (β_ALM, HIV+_: −0.470; β_LBM, HIV+_: −0.843) *Escherichia* (β_ALM, HIV+_: −0.288; β_LBM, HIV+_: −0.467) *Bacteroides* (β_ALM, HIV+_: −0.040)	α‐diversity: NA β‐diversity: NA	Not adjusted
Houttu et al., The Netherlands, 2021^31^	*N* = 1334, CC, TC	Phylum: *Euryarchaeota* (r_TC_: 0.099) *Lentisphaerea* (r_TC_: 0.127) Family: *Muribaculaceae* (r_TC_: 0.103) *Methanobacteriaceae* (r_TC_: 0.094) *Eggerthellaceae* (r_TC_: 0.101) *Atopobiaceae* (r_TC_: 0.089)Genus: *Lachnospiraceae UCG.008* (r_CC, TC_: 0.08) *Lachnospiraceae* AC2044 (r_TC_: 0.087) *Ruminococcaceae UCG.003* (r_CC_: 0.08; r_TC_: 0.127) Ruminococcaceae UCG.005 (r_TC_: 0.084) Ruminococcaceae UCG.010 (r_TC_: 0.122) *Ruminococcaceae UCG.014* (r_TC_: 0.09) *Ruminococcaceae NK4A214* (r_TC_: 0.100) *Butyriovibrio* (r_TC_: 0.096) *Methanobrevibacter* (r_TC_: 0.091) Species: *Butyriovibrio crossotus* (r_TC_: 0.09) *Dialister succinatuphilus* (r_TC_: 0.12)	Genus: *Blautia* (r_CC_: −0.085) *Turicibacter* (r_TC_: −0.085) Species: *Acidaminococcus intestini* (r _TC_: −0.13) *Turicibacter sanguinis* (r_TC_: −0.085)	α‐diversity: Shannon index (β_CC_: 0.013), richness (β_TC_: 0.003^a^; β_CC_: 5.106) and Faith's phylogenetic diversity index (β_CC_: 0.236) β‐diversity: NA	Age, sex, BMI, ethnicity, average fat‐, grain and carbohydrate intake
Hung et al., Taiwan, 2021^33^	*N* = 179, LTI%	Phylum: *Firmicutes* (*r* = 0.213)	No significant associations	F/B‐ratio: (*r* = 0.239) β‐diversity: NA	Age, sex, BMI
Kang et al., China, 2021^13^	*N* = 87, ASMI	Genus: *Eubacterium, Roseburia*	No significant associations	α‐diversity: NA β‐diversity: NA	Not adjusted
Palmas et al., Italy, 2021^37^	*N* = 92, DXA‐determined muscle mass	No significant associations	Family: *Thermicanaceae* (*r* = −0.325) Genus: *Thermicanus* (*r* = −0.325) Species: *Desulfvibrio piger* (*r* = −0.74)	α‐diversity: NA β‐diversity: NA	Age, sex, smoking
Ponziani et al., Italy, 2021^46^	*N* = 100, ALM	No significant associations	Genus: *Slackia* (*r* = 0.29)	α‐diversity: NA β‐diversity: NA	Not adjusted
Tavella et al., Italy, 2021^b^, ^52^	*N* = 201, SMI	Genus: *Christenellaceae R7* *Ruminococcaceae UCG002* *Ruminococcaceae UCG005* *Ruminococcaceae UCG014* Species: *Eubacterium rectale*	Genus: *Ruminococcus 2* *Subdoligranulum* *Fusicantenibacter* *Blautia*	α‐diversity: NA β‐diversity: NA	Not adjusted
Han et al., Taiwan, 2022^12^	*N* = 88, SMI	Genus: *Marvinbryanthia* *Ruminococcaceae* NK4A214 group sp *Christensenellaceae*R‐7 group sp. *Family XII UCG* – 001 sp. *Family XII AD3011* sp.	Genus: *Flavonifractor sp*. *Sellimonas sp*.	α‐diversity: Observed ASV (β: −61.36), Shannon index (β: −0.3084); Chao1 index (β: −63.50) β‐diversity: NA	Age, BMI, MNA‐score, physical activity level
Hu et al., China, 2022^32^	*N* = 102, MAC, MAMC, TSF	Genus: *Roseburia* (r_MAC, HD+_: 0.287 r_MAMC, HD+_ = 0.285) *Coprococcus* (r_MAC, HD+_: 0.370; r_MAMC, HD+_: 0.424)	Genus: *Escherichia* (r_MAC, PD+_: −0.343; r_MAMC, PD+_ = −0.361) *Coprococcus* (r_MAC, PD+_: −0.33; r_MAMC, PD+_: −0.18)	α‐diversity: Shannon index (r_MAC_: 0.353; r_MAMC_: 0.427) Simpson index (r_MAMC_: −0.313) β‐diversity: NA	Not adjusted
Davis et al., Australia, 2023^44^	*N* = 490, SMI	Genus: combined abundance of butyrate producing bacteria*: Coprococcus 2*, *Coprococcus 3*, *Faecalibacterium*, *Subdoligranulum*, *Roseburia*, *Anaerostipes* (β: 0.02)^a^	No significant associations	α‐diversity: No significant associations β‐diversity: No significant associations	Age, smoking, physical activity, intestinal symptoms, batch effects, medications
Grahnemo et al., Norway, 2023^55^	*N* = 5196, ALM	Species: *Dorea longicatena* (β_ALM_: ± 0.5) *Coprococcus comes* (β_ALM_: ± 0.5) *Eubacterium ventriosum* (β_ALM_: ± 0.3)		α‐diversity: NA β‐diversity: NA	Age, sex, height, fat mass, chronic disease, medication, smoking, stool consistency
Yan et al., China, 2023^42^	*N* = 276, ASMI, CC	No significant associations	Genus: *Bifidobacterium* (r_ASMI_)	α‐diversity: NA β‐diversity: NA	Age, BMI, height, weight, waistline, ALT, ALT/AST, total bilirubin
Wang et al., China, 2023^48^	*N* = 100, BIA‐derived skeletal muscle mass	Genus: *Gammaretrovirus* Species: *Bacteroides fluxus* *Barnesiella intestinihominis* *Bacteroides coprocola* *Bacteroidales bacterium ph8* *Bacteroides massiliensis* *Mitsuokella multitacida* *Bacteroides coprophilus*	Species: *Subdoligranulum variabile* *Collinsella aerofaciens* *Eggerthella lenta*	α‐diversity: NA β‐diversity: NA	

ALM, appendicular lean mass; ALT, alanine transaminase; AST, aspartate aminotransferase; BIA, bio‐electrical impedance analysis; CC, calf circumference; LTI%, lean tissue index; MAC, mid upper arm circumference; MAMC, mid‐upper arm muscle circumference; MNA, Mini‐Nutritional Assessment; SMI, skeletal muscle index; stTPA, standardized total psoas area; TC, thigh circumference; TSF, triceps skinfold thickness.

^a^ Association became non‐significant after adjustment for confounders.

^b^ Not clearly stated whether association was significant.

Six studies^31^, ^33^, ^37^, ^42^, ^46^, ^47^ reported associations between phyla and muscle mass, of which three studies found significant associations.^31^, ^33^, ^47^ Hung et al. and Houttu et al. found weak positive correlations between muscle mass and respectively *Firmicutes* and *Euryarchaeota*.^31^, ^33^ Contrarily, Dillon et al. found a negative association between appendicular lean mass and *Proteobacteria*.^47^

Five studies^31^, ^37^, ^46^, ^47^, ^50^ reported associations of GM‐families with muscle mass, of which three^31^, ^37^, ^47^ reported significant associations. However, findings were heterogeneous since one study reported positive correlations^31^ and two others reported negative associations^37^, ^47^ between GM and muscle mass.

Thirteen studies^12^, ^13^, ^31^, ^32^, ^33^, ^37^, ^42^, ^44^, ^46^, ^47^, ^48^, ^50^, ^52^ investigated associations between GM‐genera and muscle mass, of which eleven^12^, ^13^, ^31^, ^32^, ^33^, ^37^, ^44^, ^46^, ^47^, ^48^, ^52^ reported significant associations. Both positive and negative associations were reported, but findings were heterogeneous and sometimes contradictory. To illustrate, *Coprococcus* was fairly positively correlated with anthropometric estimates of muscle mass in persons on haemodialysis, whereas this genus was weakly and fairly negatively correlated with respectively mid‐upper arm muscle circumference (MAMC) and mid‐upper arm circumference in persons on peritoneal dialysis.^32^ Similarly, one study reported both weak positive and negative correlations between several *Ruminococcus* genera and SMI, however without clear statement about significance of the findings.^52^ *Roseburia* correlated weakly positively with muscle mass estimates in two studies.^13^, ^32^ Also, Davis et al. found that a combination of butyrate‐producing genera, among which *Roseburia*, was positively associated with SMI, however, after adjustment for multiple confounders, this association became non‐significant.

Eight studies^12^, ^31^, ^33^, ^37^, ^48^, ^50^, ^52^, ^55^ reported associations between muscle mass and GM‐species, of which six reported significant associations.^31^, ^37^, ^48^, ^50^, ^52^, ^55^ For studies reporting correlations, in both directions strength varied from weak (*r* < 0.3) to moderate‐high (0.6 ≤ *r* < 0.8).^23^ One study did not clearly state upon significance or strength of reported correlations.^52^

Four studies investigated associations between α‐diversity indices and muscle mass,^12^, ^31^, ^32^, ^44^ which were significant in three.^12^, ^31^, ^32^ Houttu et al.^31^ and Hu et al.^32^ reported respectively positive associations (independent of ethnicity, sex, age, BMI, and diet) and fair positive correlations with anthropometric estimates of muscle mass. The latter found a fair negative correlation between Simpson index and MAMC. Smaller Simpson indices correspond with higher biodiversity, indicating that a higher MAMC was correlated with higher biodiversity.^31^, ^32^ Han et al. reported negative associations between low muscle mass and observed ASV, Shannon and Chao1 indices,^12^ suggesting that persons with preserved or higher muscle mass had more diverse GM. Hung et al. reported a weak positive correlation between LTI% and F/B‐ratio.^33^ Two studies investigated associations between β‐diversity and muscle mass, and one found a significant association between β‐diversity and CC in long‐term care residents.^30^

#### Muscle strength

##### Relative abundance of gut microbiota taxa at multiple levels & gut microbiota diversity markers

Eight studies investigated RA of GM between persons with low and preserved muscle strength,^12^, ^28^, ^29^, ^31^, ^32^, ^40^, ^44^, ^51^ and all but one^44^ reported significantly, but heterogeneously, altered GM at all taxonomic levels comparing between persons with low and preserved muscle strength. Appendix S5a,b gives an overview of respectively significant and non‐significant findings.^12^, ^28^, ^29^, ^31^, ^32^, ^40^, ^51^

Eight studies compared α‐diversity between persons with low and preserved muscle strength,^12^, ^28^, ^29^, ^31^, ^32^, ^40^, ^44^, ^51^ of which two studies found significantly altered indices in persons with low muscle strength.^29^, ^31^ One study found significantly decreased Shannon and Richness indices in persons with low HGS, but after adjusting for age, sex, BMI, and ethnicity, this result became non‐significant.^31^ Contrarily, the other study reported decreased observed species in persons who performed more chair rises in 30 s, however, without adjustment for confounders.^29^ Seven studies investigated β‐diversity,^12^, ^28^, ^29^, ^31^, ^40^, ^44^, ^51^ and five found GM to cluster significantly different between persons with low muscle and preserved muscle strength.^28^, ^29^, ^31^, ^40^, ^51^

##### Associations between muscle strength and gut microbiota taxa and gut microbiota diversity measures

Of the nine studies^12^, ^13^, ^31^, ^32^, ^34^, ^42^, ^44^, ^46^, ^47^ investigating associations between muscle strength and GM, seven found significant associations.^12^, ^13^, ^32^, ^34^, ^42^, ^44^, ^47^ Table 4 shows significant findings, whereas non‐significant findings are given in Table S3.^12^, ^13^, ^31^, ^32^, ^34^, ^42^, ^44^, ^46^, ^47^

**Table 4:** Associations between muscle strength and multiple levels of GM taxa and GM diversity markers

Authors, country, year	Sample size, muscle strength measure	GM taxa		Diversity markers	Adjustment for confounders
		Positive associations	Negative associations		
Dillon et al., USA, 2020^47^	*N* = 36, CST time, HGS, BP, LPD, and LP	Phylum: *Proteobacteria* (β_CST, HIV+_: 0.5743) *Bacteroidetes* (β_BP, HIV−_: 0.0066; β_LP, HIV−_: 0.0068) Family: *Bacteroidaceae* (β_LPD, HIV−_: 0.0068)*; Rikenellaceae* (β_LP, HIV−_: 0.0988) Genus: *Alistipes* (β_CST, HIV+_: 2.705; β_LP, HIV−_: 0.098) *Escherichia* (β_CST, HIV+_: 1.350) *Bacteroides* (β_LP, HIV−_: 0.007) *Sutterella* (β_HGS, HIV−_: 4.206)	Family: *Lachnospiraceae* (β_CST, HIV+_: −0.432) *Coriobacteriaceae* (β_BP_,_HIV−_: −0.028; β_LPD, HIV−_: −0.041) Genus: *Dorea* (β_CST_,_HIV−_: −2.43) *Bifidobacterium* (β_PB, HIV−_: −0.074; β_LPD, HIV−_: −0.089) *Collinsella* (β_BP, HIV−_: −0.035; β_LP, HIV−_: −0.050)	α‐diversity: NA β‐diversity: NA	Not adjusted
Lim et al., South‐Korea, 2020^34^	*N* = 176, HGS	No significant associations	Species: *Bacteroides caccae* (*r* = −0.0030) *Alistipes indistinctus* (*r* = −0.0010)	α‐diversity: No significant associations reported β‐diversity: Significantly correlated with HGS	Age, sex
Houttu et al., The Netherlands, 2021^31^	*N* = 1334, HGS	No significant associations	No significant associations	α‐diversity: Species richness (β: 0.154) β‐diversity: NA	Age, sex, BMI, ethnicity, average fat‐, grain, and carbohydrate intake
Kang et al., China, 2021^13^	*N* = 87, CST time, HGS	Genus: *Lachnospira* (r_HGS_) *Ruminococcus* (r_HGS_) *Eubacterium* (r_HGS_)	Genus: *Roseburia* (r_CSTtime_) *Eubacterium rectale group* (r_CSTtime_) *Lachnospira* (r_CSTtime_)	α‐diversity: NA β‐diversity: NA	Not adjusted
Davis et al. Australia, 2021^44^	*N* = 490, HGS	Genus: *Faecalibacterium Subdoligranulum* *Roseburia* *Coprococcus 2* *Anaerostipes* *Coprococcus 3* ^a^ (β: 0.010)	Genus: No significant associations		Age, smoking, physical activity, intestinal symptoms, medications, batch effects, fat mass, Australian Recommended Food Score
Han et al., Taiwan, 2022^12^	*N* = 88; HGS	Genus: *Akkermansia sp*.	Species: *Bifidobacterium longum*	α‐diversity: NA β‐diversity: NA	Age, BMI, MNA‐score, physical activity level
Hu et al., China, 2022^32^	*N* = 102, HGS	Genus: *Roseburia* (r_HD+_: 0.41; r_PD+_: 0.292) *Coprococcus* (r_HD+_: 0.410) *Phascolarctobacterium* (r_HD+_: 0.343; r_PD+_: 0.399)	Genus: *Escherichia* (r_HD+_: −0.388) *Coprococcus* (r_PD+_: −0.33)	α‐diversity: Shannon index (r_HD+_: 0.559; r_PD+_: 0.598) Simpson index (r_HD+_: −0.440; r_PD+_: −0.503) β‐diversity: NA	Not adjusted
Yan et al., China, 2023^42^	*N* = 276, HGS	Genus: *Agathobacter*	Phylum: *Actinobacteria* Genus: *Bifidobacterium*	α‐diversity: NA β‐diversity: NA	Age, BMI, waistline, ALT, ALT/AST, total bilirubin

ALT, alanine transaminase; AST, aspartate aminotransferase; BMI, body mass index; BP, bench press; CST, chair stand test; HD, haemodialysis; HGS, hand grip strength; HIV−, persons without HIV; HIV+, persons with HIV; LP, leg press; LPD, lateral pulldown; MNA, Mini‐Nutritional Assessment; PD, peritoneal dialysis.

^a^ Result became non‐significant after adjustment for confounders.

Four studies investigated associations at the phylum level,^31^, ^42^, ^46^, ^47^ and two of these found significant associations with muscle strength.^42^, ^47^ The first study reported a fair negative correlation between *Actinobacteria* and HGS,^42^ whereas the latter reported a positive association between *Proteobacteria* and CST time and between *Bacteroidetes* and leg press and bench press (maximal weight lifted), indicating that persons with decreased lower limb strength have a higher RA of *Proteobacteria* and a lower RA of *Bacteroidetes*.^47^

Three studies investigated associations with muscle strength at family level,^31^, ^46^, ^47^ but only one reported significant positive associations between *Bacteroidaceae* and lateral pulldown (maximal weight lifted), and between *Rikenellaceae* and leg press.

All studies^12^, ^13^, ^31^, ^32^, ^34^, ^42^, ^44^, ^46^, ^47^ investigated associations between muscle strength and GM‐genera, of which six^12^, ^13^, ^32^, ^42^, ^44^, ^47^ found significant results, which became non‐significant for one study after adjustment for confounders.^44^ *Lachnospira* was positively correlated with HGS and negatively with CST time, indicating that in persons with higher strength, this genus was more abundant.^13^ Also for *Roseburia*, similar findings were reported, as this genus was fairly positively correlated with HGS^32^ in one study, and negatively correlated with CST time in another study.^13^ Two studies found *Bifidobacterium* to be negatively associated with respectively bench press and lateral pulldown in persons with HIV^47^ and negatively correlated with HGS in older women.^42^ Hu et al. reported conflicting findings regarding *Coprococcus*, being fairly positively correlated with HGS in persons on haemodialysis, but fairly negatively correlated with HGS in persons on peritoneal dialyses.^32^

Three studies^12^, ^31^, ^34^ investigated associations between GM‐species and muscle strength, of which two found significant results. More specifically, Lim et al. found *Bacteroides caccae* and *Alistipes indistinctus* to be weakly negatively correlated with HGS. Also, Han et al. identified a weak negative correlation with *Bifidobacterium longum* subspecies.^12^, ^34^

Of three studies^31^, ^32^, ^34^ investigating possible associations between muscle strength and α‐diversity, two withheld a significant positive association with observed species^31^ and Shannon indices.^32^ The latter study also found a fair negative correlation between HGS and the Simpson index. Only Lim et al. investigated and withheld a significantly weak correlation between muscle strength and β‐diversity.^34^ No studies investigated associations between muscle strength and β‐diversity.

#### Physical performance

##### Relative abundance of gut microbiota taxa at multiple levels and gut microbiota diversity markers

Four studies^27^, ^38^, ^44^, ^47^ reported differences in RA of GM in persons with low and preserved physical performance, although for one of these studies, the difference between low and preserved physical performance was borderline non‐significant (*P* = 0.05).^47^ Significantly altered GM were found at all taxonomic levels, species exempted. Appendix S6a,b gives an overview of respectively significant and non‐significant^27^, ^38^, ^44^, ^47^ alterations between persons with low and preserved physical performance.

For α‐diversity, four studies^27^, ^38^, ^44^, ^47^ investigated differences between persons with low and preserved physical performance, but none were significant. Of two studies reporting β‐diversity, one withheld significant results, indicating that persons with low physical performance clustered differently based on their GM.^27^ In the study by Davis et al., findings for β‐diversity became non‐significant after adjustment for multiple confounders.^44^

##### Associations between physical performance and gut microbiota taxa and gut microbiota diversity markers

Four studies investigated associations between physical performance and GM, of which three reported significant findings.^12^, ^44^, ^47^ Table 5 and Table S4 show respectively significant and non‐significant^12^, ^42^, ^44^, ^47^ findings.

**Table 5:** Associations between physical performance and multiple levels of GM taxa

Author, country, year	Sample size (*N*), physical performance estimate	GM taxa		Diversity markers	Adjustment for confounders
		Positive association	Negative association		
Dillon et al., USA, 2020^47^	*N* = 36, SPPB, 400 m walk test, stair climb time	Phylum: *Proteobacteria* (β_tair climb time, HIV+_: 0.1184) *Bacteroidetes* (β_400 m_,_HIV+_: 0.7443) Family: *Enterobacteriaceae* (β_stair climb time_, _HIV+_:0.1147) *Veillonellaceae* (β_stair clim, HIV+_: 0.10001) Genus: *Dorea* (β_SPPB, HIV+_: 0.243) *Prevotella* (β_stair climb, HIV+_: 0.033) *Megasphaera* (β_stair climb, HIV+_:0.251) *Phasolarctobacterium* (β_SPPB, HIV+_: 0.0068) *Bifidobacterium* (β_400 m, HIV ‐_:6.572; β_stair climb, HIV−_:0.184)	Phylum: *Proteobacteria* (β_SPPB, HIV+_: −0.9) Family: *Enterobacteriaceae* (β_SPPB, HIV+_: −0.069) *Bacteroidaceae* (β_SPPB, HIV+_: −0.028) Genus: *Bacteroides* (β_SPPB, HIV+_: −0.028) *Escherichia* (β_SPPB, HIV+_: −0.252)	α‐diversity: NA β‐diversity: NA	Not adjusted
Davis et al., Australia, 2021^44^	*N* = 490, TUG test	No significant associations	Genus: *Faecalibacterium Subdoligranulum* *Roseburia* *Coprococcus 2* *Anaerostipes* *Coprococcus 3* ^a^ (β: −0.01)	α‐diversity: No significant associations β‐diversity: No significant associations	Age, smoking, physical activity, intestinal symptoms, medications, batch effects, fat mass, Australian Recommended Food Score
Han et al., Taiwan, 2022^12^	*N* = 88, gait speed	Species: *Parabacteroides johnsonii* CL02T12C29	Species: *Streptococcus* sp. *Fusobacterium* sp.	α‐diversity: NA β‐diversity: NA	Age, BMI, MNA‐score, physical activity

BMI, body mass index; HIV, human immunodeficiency virus; MNA, Mini‐Nutritional Assessment; SPPB, Short Physical Performance Battery; TUG, Timed‐Up and Go.

^a^ Result became non‐significant after adjusting for confounders.

At the phylum and family level, one study investigated associations with physical performance, which were significant.^47^ At class and order level, no studies investigated associations with physical performance.

Three studies investigated associations between GM‐genera and physical performance,^12^, ^44^, ^47^ and two of these reported significant results.^44^, ^47^ However, the negative associations reported between butyrate‐producing genera and the TUG test time became non‐significant after adjustment for multiple confounders.^44^

The study by Han et al. was the only one to investigate correlations between GM‐species and physical performance estimates, which remained significant. Several species were negatively or positively correlated with gait speed. However, no statement about the strength of the correlations was made as exact values of correlation coefficients were not reported.^12^

Only Davis et al. reported on associations between physical performance and α‐ or β‐diversity; however, none of the associations were significant.^44^

#### Associations between gut microbiota taxa and muscle mass, strength and physical performance

Only Dillon et al. reported on associations between all three sarcopenia‐defining parameters and GM phyla, families and genera in persons with and without HIV.^47^ More specifically, in older persons with HIV, greater RA of *Proteobacteria*, *Enterobacteriaceae*, *Bacteroidaceae*, *Escherichia*, *Prevotella*, *Megasphaera*, *Subdoligranulum*, and *Bacteroides* were associated with reduced muscle mass, reduced lower limb strength or lower physical performance. Contrarily, greater RA of *Lachnospiraceae*, *Coprococcus*, *Alistipes*, *Catenibacterium*, and *Phascolarctobacterium* was significantly associated with improved muscle mass, better lower limb strength, and higher physical performance in older persons with HIV (Tables 3, 4, and 5).

## Discussion

This systematic review pioneered by summarizing existing literature regarding GM‐signatures for sarcopenia, through describing GM RA and diversity at multiple levels in persons aged ≥50 years with sarcopenia or a decrease in one of its defining parameters, as well as through exploring the associations between the GM and these muscle parameters.

The abundance of GM‐taxa was significantly altered in persons with sarcopenia, low muscle mass, low muscle strength, and physical performance. Studies reported uniform findings regarding RA of *Faecalibacterium prausnitzii* , which was lower in persons with sarcopenia, low muscle mass and low muscle strength. Depleted levels of this butyrate‐producing species have been linked to various pathologies, especially IBD, with a high sarcopenia prevalence.^58^ Another butyrate‐producer, *Roseburia*, was consistently lower in persons with sarcopenia^13^, ^39^ and low muscle mass.^32^ Therefore, butyrate‐producers could be taken into account when determining a sarcopenia‐related GM‐signature.

However, generally, differences in GM RA in persons with low versus preserved muscle parameters, and the direction of the alterations of GM (increase or decrease) were heterogeneous and at times counterintuitive. To illustrate, *Lactobacillus* was increased in persons with sarcopenia,^43^, ^48^ low muscle mass,^35^ and low muscle strength.^40^ Mostly *Lactobacillus* does not reside in the gut, and increase could potentially reflect intake of probiotic (‐enriched)‐compounds, underscoring the need to record these potential sources of bias. Also *Bifidobacterium* was increased in persons with sarcopenia in one study,^43^ but decreased in another.^48^ It would be expected that levels of these strains are lower in persons with decreased muscle status, which was not consistently the case in our systematic review. A compensatory mechanism could potentially explain the increase of *Lactobacillus* and *Bifidobacterium* in persons with low muscle status. Also, age‐related decreases in other bacterial taxa could potentially cause bias in levels of other strains such as Lactobacillus and Bifidobacterium, resulting in an artificial increase.

Difference in β‐diversity showed consistently that persons with sarcopenia, low muscle mass and low strength clustered significantly different based on GM compared with persons with preserved muscle status in respectively 6/10, 3/6, and 5/7 studies, indicating that GM‐composition could distinguish between low and preserved muscle status. Concerning difference in α‐diversity, persons with sarcopenia, low muscle mass or strength had less rich/diverse GM, in respectively 6/11, 4/7, and 2/8 studies. For the latter group, results became non‐significant after adjustment for confounders in one study. The F/B ratio was increased in persons with sarcopenia,^48^ but no statement of significance was made in the paper.

This review also investigated associations between GM taxa and sarcopenia or its defining parameters. Reported correlations mainly varied between weak (*r* < 0.3) and fair (0.3 ≥ *r* < 0.6), with heterogeneous directions. For *Lactobacillus*, association findings were somewhat in line with RA findings, since the genus was significantly positively correlated with sarcopenia.^49^ In contrast to our expectations, *Bifidobacterium* was negatively associated with respectively HGS^12^, ^42^ and ASMI.^42^ These results were in line with the RA findings for *Bifidobacterium*. Also, for *Roseburia*, findings from RA analyses remained in line with association analyses since different studies reported significant weak positive correlations between this genus and muscle mass and strength.^13^, ^32^ Contrarily, *Faecalibacterium prausnitzii* RA findings were not confirmed in association analyses. The *Faecalibacterium* genus was negatively associated with physical performance and muscle mass, becoming non‐significant after adjustment for confounders, while its RA was significantly lower in persons with sarcopenia, low muscle mass and low strength.

Previous studies identified specific GM enterotypes, such as the Bacteroides2 enterotype, which is prevalent in several diseases^59^, ^60^ and associated with increased systemic inflammation.^60^ In this review, two studies reported on enterotypes; however, no significant associations with sarcopenia (‐defining parameters) were investigated.

For studies reporting significant associations with different α‐diversity markers, findings were in line with the above stated differences in α‐diversity (RA findings) for muscle mass and strength, since significant positive associations between α‐diversity and these two sarcopenia‐defining parameters were reported in all studies. No significant associations between α‐ or β‐diversity and physical performance or sarcopenia as a construct were reported. However, confounders often linked with alpha diversity (e.g., inflammation and transit time) were often not controlled for.

Generally, results derived from GM diversity analyses were more uniform compared with associations with individual GM taxa, suggesting that persons suffering from sarcopenia or decreased muscle parameters may have a different GM‐composition (β‐diversity). Moreover, a trend towards less diverse and less rich GM is reported in these persons compared with those with preserved muscle status (α‐diversity). However, these diversity markers do not give information on decreases or increases of specific taxa in persons with sarcopenia, for which RA data are needed.

## Strengths and limitations

To our knowledge, this was the first systematic review to compare RA of GM at multiple levels in persons with and without low muscle status or sarcopenia, and to describe associations between sarcopenia or its defining parameters on the one hand and multiple levels of GM‐taxa and markers of GM diversity on the other hand. This review only included studies in humans aged ≥50 years, contrarily to previous reviews.^61^, ^62^

Some limitations need to be addressed. First, due to the large quantity of findings, this systematic review emphasizes significant results. To address this limitation, a complete overview of all non‐significant RA and association findings has been added to the appendices, which was insufficiently addressed in prior research.^18^ It is important to note that these data should be interpreted with caution. Indeed non‐significant differences in RA were often derived from graphs without statement of exact levels or *P*‐values. For non‐significant associations data were often derived from heath plots, without further specification of correlation coefficients or *P*‐values. No meta‐analysis was conducted for several reasons, the first one being heterogeneity of the results. Additionally, included studies often did not comprise similar comparators. Finally, this review aimed to elucidate a potential GM‐signature of sarcopenia comprising multiple GM‐taxa. As changes in multiple taxa would potentially be non‐linear, it could complicate meta‐analysis.

Second, only studies assessing GM at one timepoint were included. GM are highly susceptible to day‐day variability,^63^ thus considering one timepoint potentially causes bias.

Third, included studies used a pleiotropy of definitions (due to lack of a universal definition) and tools to assess respectively sarcopenia or its defining parameters.^64^ Preferred tools to assess sarcopenia‐defining parameters differ according to setting, implying some bias.^65^ Moreover, sarcopenia was assessed as a binary variable, although prior aging research indicated that a composite score for sarcopenia comprising muscle mass, strength and physical performance as continuous measures, might better capture aging phenotypes.^66^ However, it is currently also being debated by expert groups whether all these three should be included in the definition of sarcopenia.^67^ All these factors could potentially declare some of the contradictory findings regarding RA and association analyses of some GM, such as *Faecalibacterium prausnitzii* .

Fourth, 12/32 studies adjusted for at least one confounder, mostly age. Ethnicity, dietary intake, smoking, batch effects, and bowel habits (e.g., transit time) influence GM and should be corrected for in future studies.^68^, ^69^, ^70^ In this review, only two studies corrected for batch effects^44^, ^55^ and one study for stool consistency, a proxy for transit time.^55^ Prior research reported longer colonic transit times with increasing age, but data in persons with sarcopenia are lacking.^71^ Also medications (e.g., antibiotics) significantly impact GM^72^ and of 22 studies reporting antibiotics intake as an exclusion criterium, only two studies adjusted for ‘medications potentially affecting the gut’. One included study stated that all persons received antibiotics prophylaxis prior to planned surgical intervention.^44^, ^50^, ^55^ Furthermore, time windows between last antibiotics intake and study inclusion varied between ‘ad hoc’ and ‘six months preceding study participation’. Also non‐antibiotic medications, such as statins are associated with GM and sarcopenia.^60^, ^73^ Finally, only 13/32 studies corrected analyses for multiple testing, which is important when testing many taxa, since multiple testing increases the risk of a type I error. This implies that one could incorrectly rejects a null hypothesis more often.

Fifth, heterogeneity of our findings can partly be explained by differences in assessed GM‐taxonomic levels, sample sizes and faecal sample preservation and DNA‐extraction procedures.^14^, ^74^ Sample preservation at −80° ensures optimal stability of GM up to years. However, 4/32 included studies did not state preservation methods of faecal samples^30^, ^32^, ^34^, ^41^ or stated about temporarily preservation at ‘ambient’ temperatures before transfer to −80°C.^26^, ^27^, ^29^, ^51^ Thus, identification of sarcopenia‐related GM‐markers requires standardized faecal sample preservation and analyses procedures as well as development of a core outcome set (COS) specifically for GM‐assessment in persons with age‐related conditions.^75^, ^76^, ^77^ Also differences in person characteristics of included studies contributed to heterogeneity. To illustrate, in 4/13 studies defining sarcopenia as a construct, persons suffered from co‐morbidity (e.g., liver cirrhosis) influencing muscle, and therefore, sarcopenia could be considered as secondary. One study stated persons to have primary sarcopenia,^39^ but remaining studies insufficiently specified the nature of sarcopenia. As co‐morbidity might influence GM, this should be accounted for in future studies exploring the GM‐muscle axis.

## Conclusions and future perspectives

GM were associated with sarcopenia and estimates of its defining parameters, but directions of associations were heterogeneous, and strength of reported correlations was often only ‘fair’. However, results from analyses of α‐ and β‐diversity were more uniform. GM of persons with sarcopenia, low muscle mass and low strength clustered differently from persons with preserved muscle status (β‐diversity). GM of persons with sarcopenia, low muscle mass, and strength are less diverse/rich (α‐diversity) compared with persons with preserved muscle status although not all known affecting confounders were controlled for. RA of specific GM‐taxa should be further explored in order to identify specific sarcopenia‐related GM‐markers. In our review, findings from RA of GM‐taxa at multiple levels were highly heterogeneous and sometimes counterintuitive, potentially due to differences in study set‐up, population characteristics and faecal sample processing, hindering qualitative analysis and warranting caution in extrapolating findings. The cross‐sectional character of included studies hampers to draw causal conclusions, underscoring the need for more longitudinal trials with sufficiently large sample sizes, well‐phenotyped clinical data, confounder control (e.g., dietary intake, bowel habits, and drug intake), clearly defined outcomes and uniform faecal sampling procedures in order to reduce heterogeneity and to identify a GM markers of sarcopenia. Finally, future research should focus on analyses of sequencing data of included studies, to gain in depth insight in GM‐composition of persons with sarcopenia.

## Conflict of interest

Laurence Lapauw declares that she has no conflict of interest. Aurélie Rutten declares that she has no conflict of interest. Jolan Dupont declares that he has no conflict of interest. Nadjia Amini declares that she has no conflict of interest. Laura Vercauteren declares that she has no conflict of interest. Muriel Derrien declares that she has no conflict of interest. Jeroen Raes declares that he has no conflict of interest. Evelien Gielen declares she has no conflict of interest.

## Supporting information


**Appendix S1.** Search Strategy
**Appendix S2.** Synopsis of the results
**Appendix S3.** (a) Abundance of GM taxa and markers of GM diversity in persons with sarcopenia: significant findings. (b) Abundance of GM taxa and markers of GM diversity in persons with sarcopenia: non‐significant findings
**Appendix S4.** (a) Abundance of GM taxa and markers of GM diversity in persons with low muscle mass: signficant findings. (b) Abundance of GM taxa and GM diversity markers in persons with low muscle mass: non‐significant findings
**Appendix S5.** (a) Abundance of GM taxa and GM diversity markers in persons with low muscle strength: significant findings. (b) Abundance of GM taxa and GM diversity markers in persons with low muscle strength: non‐significant findings
**Appendix S6.** (a) Abundance of GM taxa and GM diversity markers in persons with low physical performance: significant findings. (b) Abundance of GM taxa and GM diversity markers in persons with low physical performance: non‐significant findings
**Appendix S7.** Quality assessment of included studies according to the Newcastle‐Ottawa Scale (NOS)
**Table S1.** Associations between GM taxa and sarcopenia as a construct: non‐significant findings
**Table S2.** Associations between GM taxa and muscle mass: non‐significant findings
**Table S3.** Associations between GM taxa and muscle strength: non‐significant findings
**Table S4.** Associations between GM taxa and physical performance: non‐significant findings
