# Supplementary material for: Associations between gut microbiota and sarcopenia or its defining parameters in older adults: A systematic review
Source: J Cachexia Sarcopenia Muscle. 2024 Aug 27;15(6):2190–207. doi: 10.1002/jcsm.13569 (PMC11634501; doi:10.1002/jcsm.13569)
Supplement: Supplementary file 1 — Appendix S1. Search Strategy Appendix S2. Synopsis of the results Appendix S3. (a) Abundance of GM taxa and markers of GM diversity in persons with sarcopenia: significant findings. (b) Abundance of GM taxa and markers of GM diversity in persons with sarcopenia: non‐significant findings Appendix S4. (a) Abundance of GM taxa and markers of GM diversity in persons with low muscle mass: signficant findings. (b) Abundance of GM taxa and GM diversity markers in persons with low muscle mass: non‐significant findings Appendix S5. (a) Abundance of GM taxa and GM diversity markers in persons with low muscle strength: significant findings. (b) Abundance of GM taxa and GM diversity markers in persons with low muscle strength: non‐significant findings Appendix S6. (a) Abundance of GM taxa and GM diversity markers in persons with low physical performance: significant findings. (b) Abundance of GM taxa and GM diversity markers in persons with low physical performance: non‐significant findings Appendix S7. Quality assessment of included studies according to the Newcastle‐Ottawa Scale (NOS) Table S1. Associations between GM taxa and sarcopenia as a construct: non‐significant findings Table S2. Associations between GM taxa and muscle mass: non‐significant findings Table S3. Associations between GM taxa and muscle strength: non‐significant findings Table S4. Associations between GM taxa and physical performance: non‐significant findings [file JCSM-15-2190-s001.docx]

Supplementary materials: Contents

[Appendix I Search Strategy 2](#_Toc167197798)

[Appendix II Synopsis of the results 7](#_Toc167197799)

[Appendix III Abundance of GM taxa and markers of GM diversity in persons with sarcopenia: significant findings 14](#_Toc167197800)

[Appendix IIIb Abundance of GM taxa and markers of GM diversity in persons with sarcopenia: non-significant findings 18](#_Toc167197801)

[Appendix IV Abundance of GM taxa and markers of GM diversity in persons with low muscle mass: signficant findings 22](#_Toc167197802)

[Appendix IVb: Abundance of GM taxa and GM diversity markers in persons with low muscle mass: non-significant findings 24](#_Toc167197803)

[Appendix V Abundance of GM taxa and GM diversity markers in persons with low muscle strength: significant findings 27](#_Toc167197804)

[Appendix Vb: Abundance of GM taxa and GM diversity markers in persons with low muscle strength: non-significant findings 29](#_Toc167197805)

[Appendix VI Abundance of GM taxa and GM diversity markers in persons with low physical performance: significant findings 32](#_Toc167197806)

[Appendix VIb Abundance of GM taxa and GM diversity markers in persons with low physical performance: non-significant findings 33](#_Toc167197807)

[Appendix VII Quality assessment of included studies according to the Newcastle-Ottawa Scale (NOS) 34](#_Toc167197812)

Table S1: Associations between GM taxa and sarcopenia as a construct: non-significant findings 36

Table S2: Associations between GM taxa and muscle mass: non-significant findings 38

[Table S3: Associations between GM taxa and muscle strength: non-significant findings 48](#_Toc167197808)

[Table S4: Associations between GM taxa and physical performance: non-significant findings 56](#_Toc167197810)

[PRISMA checklist 62](#_Toc167197813)

## Appendix I Search Strategy

**MEDLINE via PubMed**

"Muscular Atrophy"[Mesh] OR "muscular atroph*"[tiab] OR “muscle atroph*”[tiab] OR sarcopenia*[tiab] OR “Muscle Weakness”[Mesh] OR “muscle weakness*”[tiab] OR “muscular weakness*”[tiab] OR “Muscle Strength”[Mesh] OR “muscle strength”[tiab] OR “muscular strength”[tiab] OR “Physical Functional Performance”[Mesh] OR “functional performan*”[tiab] OR “physical performan*”[tiab] OR “anabolic resistan*”[tiab] OR "Frailty"[Mesh] OR frail*[tiab] OR “muscle mass”[tiab] OR “muscular mass”[tiab] OR “muscle aging*”[tiab] OR “muscular aging*”[tiab] OR “muscle qualit*”[tiab] OR “muscle gut”[tiab] OR “gut muscle axis”[tiab] OR “muscle cell degeneration*”[tiab] OR “muscle degeneration*”[tiab] OR “muscular degeneration*”[tiab] OR “muscle fiber atroph*”[tiab] OR “muscle fiber degeneration*”[tiab] OR “muscle recession*”[tiab] OR “muscular recession*”[tiab] OR “muscle wasting*”[tiab] OR “myoatroph*”[tiab] OR “myodegeneration*”[tiab] OR “myofibrillar degeneration*”[tiab] OR “muscle insufficien*”[tiab] OR “muscular insufficien*”[tiab] OR “muscle volume*”[tiab] OR “muscular volume*”[tiab] OR “muscle force*”[tiab] OR “muscle power*”[tiab] OR “muscular force*”[tiab] OR “muscular power*”[tiab] OR (muscle[tiab] AND “gut axis”[tiab]) OR (muscle[tiab] AND “dynamic strength”[tiab]) OR (muscular[tiab] AND “dynamic strength”[tiab])

AND

"Gastrointestinal Microbiome"[Mesh] OR “Microbiome*"[tiab] OR “gut microflora”[tiab] OR “gut

microorganism*”[tiab] OR “gut microbiota*”[tiab] OR “gut flora”[tiab] OR “gastrointestinal

flora”[tiab] OR “gastrointestinal microorganism*”[tiab] OR “gastrointestinal microbiota*”[tiab] OR

“gastrointestinal microbial communit*”[tiab] OR “gastrointestinal microflora”[tiab] OR “intestinal

microbiota*”[tiab] OR “intestinal microflora”[tiab] OR “intestinal flora”[tiab] OR “intestinal

microorganism*”[tiab] OR “intestine flora”[tiab] OR “intestine microbiota*”[tiab] OR “intestine

microflora”[tiab] OR “intestine microorganism*”[tiab] OR “enteric bacteri*”[tiab] OR “intestine

bacteri*”[tiab] OR “intestinal bacteri*”[tiab] OR "Dysbiosis"[Mesh] OR “Dysbios*”[tiab] OR

“disbios*”[tiab] OR “dysbacterios*”[tiab] OR “disbacterios*”[tiab] OR "Microbiota"[Mesh] OR “faecal

microbiota*”[tiab] OR “fecal microbiota*”[tiab] OR “faecal microflora”[tiab] OR “fecal

microflora”[tiab] OR “faecal flora”[tiab] OR “fecal flora”[tiab] OR “stool microbiota*”[tiab] OR “stool

microflora”[tiab] OR “stool flora”[tiab] OR “Prebiotics”[Mesh] OR “Prebiotic*”[tiab] OR

“Probiotics”[Mesh] OR “Probiotic*”[tiab] OR “Synbiotics”[Mesh] OR “Synbiotic*”[tiab] OR “colon

flora”[tiab] OR “bowel flora”[tiab] OR “bowel microflora”[tiab] OR “bowel microbiota*”[tiab] OR

“digestive tract flora”[tiab] OR “enteric flora”[tiab] OR “enteric microflora”[tiab] OR “enteric

microorganism*”[tiab] OR “enteric microbiota*”[tiab] OR “gastro intestinal flora”[tiab] OR

“gastrointestinal tract flora”[tiab] OR “gut bacteri*”[tiab] OR “intestinal microb*”[tiab] OR “intestine

microb*”[tiab] OR “gut microb*”[tiab] OR “colon microb*”[tiab] OR “colonic microb*”[tiab] OR

“gastrointestinal microb*”[tiab] OR “intestinal tract flora”[tiab] OR “colon bacteri*”[tiab] OR “colon

microbiota*”[tiab] OR “colon microflora”[tiab] OR “colon microorganism*”[tiab] OR “colonic

bacteri*”[tiab] OR “colonic flora”[tiab] OR “colonic microbiota*”[tiab] OR “colonic microflora”[tiab]

OR “colonic microorganism*”[tiab] OR (dys[tiab] AND symbios*[tiab]) OR (“alimentary canal”[tiab]

AND flora[tiab]) OR (“alimentary tract”[tiab] AND flora[tiab]) OR (bowel[tiab] AND

microorganism*[tiab]) OR (“digestive canal”[tiab] AND flora[tiab]) OR (“gastrointestinal canal”[tiab]

AND flora[tiab]) OR (“intestinal canal”[tiab] AND flora[tiab])

**EMBASE**

'muscle atrophy'/exp OR ‘muscular atroph*’:ti,ab,kw OR ‘muscle atroph*’:ti,ab,kw OR sarcopenia*:ti,ab,kw OR ‘Muscle Weakness’/exp OR ‘muscle weakness*’:ti,ab,kw OR ‘muscular weakness*’:ti,ab,kw OR ‘Muscle Strength’/exp OR ‘muscle strength’:ti,ab,kw OR ‘muscular strength’:ti,ab,kw OR ‘Physical performance’/exp OR ‘functional performan*’:ti,ab,kw OR ‘physical performan*’:ti,ab,kw OR ‘anabolic resistan*’:ti,ab,kw OR ‘Frailty’/exp OR frail*:ti,ab,kw OR ‘muscle mass’/exp OR ‘muscle mass’:ti,ab,kw OR ‘muscular mass’:ti,ab,kw OR ‘muscle aging*’:ti,ab,kw OR ‘muscular aging*’:ti,ab,kw OR ‘muscle qualit*’:ti,ab,kw OR ‘muscle gut’:ti,ab,kw OR ‘gut muscle axis’:ti,ab,kw OR ‘muscle cell degeneration*’:ti,ab,kw OR ‘muscle degeneration*’:ti,ab,kw OR ‘muscular degeneration*’:ti,ab,kw OR ‘muscle fiber atroph*’:ti,ab,kw OR ‘muscle fiber degeneration*’:ti,ab,kw OR ‘muscle recession*’:ti,ab,kw OR ‘muscular recession*’:ti,ab,kw OR ‘muscle wasting*’:ti,ab,kw OR ‘myoatroph*’:ti,ab,kw OR ‘myodegeneration*’:ti,ab,kw OR ‘myofibrillar degeneration*’:ti,ab,kw OR ‘muscle insufficien*’:ti,ab,kw OR ‘muscular insufficien*’:ti,ab,kw OR ‘muscle volume*’:ti,ab,kw OR ‘muscular volume*’:ti,ab,kw OR ‘muscle dynamic strength’:ti,ab,kw OR ‘muscle force*’:ti,ab,kw OR ‘muscle power*’:ti,ab,kw OR ‘muscular dynamic strength’:ti,ab,kw OR ‘muscular force*’:ti,ab,kw OR ‘muscular power*’:ti,ab,kw

AND

‘Gastrointestinal Microbiome’:ti,ab,kw OR ‘Microbiome*’:ti,ab,kw OR ‘gut microflora’:ti,ab,kw OR ‘gut microorganism*’:ti,ab,kw OR ‘gut microbiota*’:ti,ab,kw OR ‘gut flora’:ti,ab,kw OR ‘gut microbiome’:ti,ab,kw OR ‘gastrointestinal flora’:ti,ab,kw OR ‘gastrointestinal microorganism*’:ti,ab,kw OR ‘gastrointestinal microbiota*’:ti,ab,kw OR ‘gastrointestinal microbial communit*’:ti,ab,kw OR ‘gastrointestinal microflora’:ti,ab,kw OR ‘intestinal microbiota*’:ti,ab,kw OR ‘intestinal microflora’:ti,ab,kw OR ‘intestinal flora’:ti,ab,kw OR ‘intestinal microorganism*’:ti,ab,kw OR ‘intestinal microbiome’:ti,ab,kw OR ‘intestine flora’/exp OR ‘intestine microbiota*’:ti,ab,kw OR ‘intestine microflora’:ti,ab,kw OR ‘intestine microorganism*’:ti,ab,kw OR ‘intestine microbiome’:ti,ab,kw OR ‘enteric bacteri*’:ti,ab,kw OR ‘intestine bacteri*’:ti,ab,kw OR ‘intestinal bacteri*’:ti,ab,kw OR ‘Dysbiosis’/exp OR ‘Dysbios*’:ti,ab,kw OR ‘disbios*’:ti,ab,kw OR ‘dys symbios*’:ti,ab,kw OR ‘dysbacterios*’:ti,ab,kw OR ‘disbacterios*’:ti,ab,kw OR ‘Microbiota’/exp OR ‘faecal microbiota*’:ti,ab,kw OR ‘fecal microbiota*’:ti,ab,kw OR ‘faecal microflora’:ti,ab,kw OR ‘fecal microflora’:ti,ab,kw OR ‘faecal flora’:ti,ab,kw OR ‘fecal flora’:ti,ab,kw OR ‘stool microbiota*’:ti,ab,kw OR ‘stool microflora’:ti,ab,kw OR ‘stool flora’:ti,ab,kw OR ‘Prebiotics’/exp OR ‘Prebiotic*’:ti,ab,kw OR ‘Probiotics’/exp OR ‘Probiotic*’:ti,ab,kw OR ‘Synbiotics’/exp OR ‘Synbiotic*’:ti,ab,kw OR ‘colon flora’:ti,ab,kw OR ‘alimentary canal flora’:ti,ab,kw OR ‘alimentary tract flora’:ti,ab,kw OR ‘bowel flora’:ti,ab,kw OR ‘bowel microflora’:ti,ab,kw OR ‘bowel microorganism*’:ti,ab,kw OR ‘bowel microbiota*’:ti,ab,kw OR ‘bowel microbiome’:ti,ab,kw OR ‘digestive canal flora’:ti,ab,kw OR ‘digestive tract flora’:ti,ab,kw OR ‘enteric flora’:ti,ab,kw OR ‘enteric microflora’:ti,ab,kw OR ‘enteric microorganism*’:ti,ab,kw OR ‘enteric microbiota*’:ti,ab,kw OR ‘enteric microbiome’:ti,ab,kw OR ‘gastro intestinal flora’:ti,ab,kw OR ‘gastrointestinal canal flora’:ti,ab,kw OR ‘gastrointestinal tract flora’:ti,ab,kw OR ‘gastrointestine flora’:ti,ab,kw OR ‘gastrointestine tract flora’:ti,ab,kw OR ‘gastrointestine microbiome*’:ti,ab,kw OR ‘gastrointestine microorganism*’:ti,ab,kw OR ‘gastrointestine microbiota*’:ti,ab,kw OR ‘gastrointestine microbial communit*’:ti,ab,kw OR ‘gastrointestine microflora’:ti,ab,kw OR ‘gut bacteri*’:ti,ab,kw OR ‘intestinal canal flora’:ti,ab,kw OR ‘intestinal microb*’:ti,ab,kw OR ‘intestine microb*’:ti,ab,kw OR ‘gut microb*’:ti,ab,kw OR ‘colon microb*’:ti,ab,kw OR ‘colonic microb*’:ti,ab,kw OR ‘gastrointestinal microb*’:ti,ab,kw OR ‘gastrointestin microb*’:ti,ab,kw OR ‘intestinal tract flora’:ti,ab,kw OR ‘intestine microbial flora’:ti,ab,kw OR ‘colon bacteri*’:ti,ab,kw OR ‘colon microbiota*’:ti,ab,kw OR ‘colon microflora’:ti,ab,kw OR ‘colon microorganism*’:ti,ab,kw OR ‘colonic bacteri*’:ti,ab,kw OR ‘colonic flora’:ti,ab,kw OR ‘colonic microbiota*’:ti,ab,kw OR ‘colonic microflora’:ti,ab,kw OR ‘colonic microorganism*’:ti,ab,kw

**Web of Science Core Collection**

TS=(“muscular atroph*” OR “muscle atroph*” OR sarcopenia* OR “muscle weakness*” OR “muscular weakness*” OR “muscle strength” OR “muscular strength” OR “functional performanc*” OR “physical performanc*” OR “anabolic resistan*” OR frail* OR “muscle mass” OR “muscular mass” OR “muscle aging*” OR “muscular aging*” OR “muscle qualit*” OR “muscle gut” OR “gut muscle axis” OR “muscle cell degeneration*” OR “muscle degeneration*” OR “muscular degeneration*” OR “muscle fiber atroph*” OR “muscle fiber degeneration*” OR “muscle recession*” OR “muscular recession*” OR “muscle wasting*” OR “myoatroph*” OR “myodegeneration*” OR “myofibrillar degeneration*” OR “muscle insufficien*” OR “muscular insufficien*” OR “muscle volume*” OR “muscular volume*” OR “muscle dynamic strength” OR “muscle force*” OR “muscle power*” OR “muscular dynamic strength” OR “muscular force*” OR “muscular power*”)

AND

TS=( “Microbiome*” OR “gut microflora” OR “gut microorganism*” OR “gut microbiota*” OR “gut flora” OR “gastrointestinal flora” OR “gastrointestinal microorganism*” OR “gastrointestinal microbiota*” OR “gastrointestinal microbial communit*” OR “gastrointestinal microflora” OR “intestinal microbiota*” OR “intestinal microflora” OR “intestinal flora” OR “intestinal microorganism*” OR “intestine microbiota*” OR “intestine microflora” OR “intestine microorganism*” OR “enteric bacteri*” OR “intestine bacteri*” OR “intestinal bacteri*” OR “Dysbios*” OR “disbios*” OR “dys symbios*” OR “dysbacterios*” OR “disbacterios*” OR “faecal microbiota*” OR “fecal microbiota*” OR “faecal microflora” OR “fecal microflora” OR “faecal flora” OR “fecal flora” OR “stool microbiota*” OR “stool microflora” OR “stool flora” OR “Prebiotic*” OR “Probiotic*” OR “Synbiotic*” OR “colon flora” OR “alimentary canal flora” OR “alimentary tract flora” OR “bowel flora” OR “bowel microflora” OR “bowel microorganism*” OR “bowel microbiota*” OR “digestive canal flora” OR “digestive tract flora” OR “enteric flora” OR “enteric microflora” OR “enteric microorganism*” OR “enteric microbiota*” OR “gastro intestinal flora” OR “gastrointestinal canal flora” OR “gastrointestinal tract flora” OR “gastrointestine flora” OR “gastrointestine tract flora” OR “gastrointestine microorganism*” OR “gastrointestine microbiota*” OR “gastrointestine microbial communit*” OR “gastrointestine microflora” OR “gut bacteri*” OR “intestinal canal flora” OR “intestinal microb*” OR “intestine microb*” OR “gut microb*” OR “colon microb*” OR “colonic microb*” OR “gastrointestinal microb*” OR “gastrointestin microb*” OR “intestinal tract flora” OR “intestine microbial flora” OR “colon bacteri*” OR “colon microbiota*” OR “colon microflora” OR “colon microorganism*” OR “colonic bacteri*” OR “colonic flora” OR “colonic microbiota*” OR “colonic microflora” OR “colonic microorganism*”)

**Cochrane library**

*Concept one:

#1: ([mh “Muscular Atrophy”] OR [mh “Muscle Weakness”] OR [mh “Muscle Strength”] OR [mh “Physical Functional Performance”] OR [mh “Frailty”])

#2: ((muscular NEXT atroph*) OR (muscle NEXT atroph*) OR sarcopenia* OR (muscle NEXT weakness*) OR (muscular NEXT weakness*) OR “muscle strength” OR “muscular strength” OR (functional NEXT performan*) OR (physical NEXT performan*) OR (anabolic NEXT resistan*) OR frail* OR “muscle mass” OR “muscular mass” OR (muscle NEXT aging*) OR (muscular NEXT aging*) OR (muscle NEXT qualit*) OR “muscle gut” OR “gut muscle axis” OR (muscle NEXT cell NEXT degeneration*) OR (muscle NEXT degeneration*) OR (muscular NEXT degeneration*) OR (muscle NEXT fiber NEXT atroph*) OR (muscle NEXT fiber NEXT degeneration*) OR (muscle NEXT recession*) OR (muscular NEXT recession*) OR (muscle NEXT wasting*) OR myoatroph* OR myodegeneration* OR (myofibrillar NEXT degeneration*) OR (muscle NEXT insufficien*) OR (muscular NEXT insufficien*) OR (muscle NEXT volume*) OR (muscular NEXT volume*) OR (muscle NEXT force*) OR (muscle NEXT power*) OR (muscular NEXT force*) OR (muscular NEXT power*) OR “muscle gut axis” OR “muscle dynamic strength” OR “muscular dynamic strength”):ti,ab,kw

#3: #1 OR #2

*Concept two:

#4: ([mh "Gastrointestinal Microbiome"] OR [mh "Dysbiosis"] OR [mh “Probiotics”] OR [mh “Prebiotics”] OR [mh “Synbiotics”] OR [mh “Microbiota”])

#5: (Microbiome* OR “gut microflora” OR (gut NEXT microorganism*) OR (gut NEXT microbiota*) OR “gut flora” OR “gastrointestinal flora” OR (gastrointestinal NEXT microorganism*) OR (gastrointestinal NEXT microbiota*) OR (gastrointestinal NEXT microbial NEXT communit*) OR “gastrointestinal microflora” OR (intestinal NEXT microbiota*) OR “intestinal microflora” OR “intestinal flora” OR (intestinal NEXT microorganism*) OR “intestine flora” OR (intestine NEXT microbiota*) OR “intestine microflora” OR (intestine NEXT microorganism*) OR (enteric NEXT bacteri*) OR (intestine NEXT bacteri*) OR (intestinal NEXT bacteri*) OR Dysbios* OR disbios* OR dysbacterios* OR disbacterios* OR (faecal NEXT microbiota*) OR (fecal NEXT microbiota*) OR “faecal microflora” OR “fecal microflora” OR “faecal flora” OR “fecal flora” OR (stool NEXT microbiota*) OR “stool microflora” OR “stool flora” OR Prebiotic* OR Probiotic* OR Synbiotic* OR “colon flora” OR “bowel flora” OR “bowel microflora” OR (bowel NEXT microbiota*) OR “digestive tract flora” OR “enteric flora” OR “enteric microflora” OR “enteric microorganism” OR (enteric NEXT microbiota*) OR “gastrointestinal flora” OR “gastrointestinal tract flora” OR (gut NEXT bacteri*) OR (intestinal NEXT microb*) OR (intestine NEXT microb*) OR (gut NEXT microb*) OR (colon NEXT microb*) OR (colonic NEXT microb*) OR (gastrointestinal NEXT microb*) OR “intestinal tract flora” OR (colon NEXT bacteri*) OR (colon NEXT microbiota*) OR “colon microflora” OR (colon NEXT microorganism*) OR (colonic NEXT bacteri*) OR “colonic flora” OR (colonic NEXT microbiota*) OR “colonic microflora” OR (colonic NEXT microorganism*) OR (dys NEXT symbios*) OR “alimentary canal flora” OR “alimentary tract flora” OR (bowel NEXT microorganism*) OR “digestive canal flora” OR “gastrointestinal canal flora” OR “intestinal canal flora”):ti,ab,kw

#6: #4 OR #5

*Combination concept one and concept two:

#7: #3 AND #6

**ClinicalTrials.gov**

Condition or disease: Sarcopenia; Study type: Observational; Study Results: Studies with Results;

Status: Completed, Eligibility: Adult (18–64), Older adults (+65)

## Appendix II Synopsis of the results

| Authors,  Country, Year | Study design, population | Comorbidities? | Assessment of gut microbiota | Assessment of sarcopenia (-defining parameter) | Sample size | Adjustment for confounders; intake of anti-, pre- or probiotics | Main results |
| --- | --- | --- | --- | --- | --- | --- | --- |
| Akashi et al., Japan, 2019 | Cohort, hospitalized persons  Age range [59.6 – 72.5]; mean age 66.9 | **Cancer (bile duct, pancreatic, esophagal)** | 16S rRNA gene sequencing, no info on regions sequenced | Standardized total psoas area (stTPA) via CT | N = 127; ♀ = 32 | No adjustment for confounders  Antibiotics administered 30 min before surgery | Fecal levels of predominant obligate anaerobes were positively correlated with stTPA, but not significantly.  Facultative negative anaerobes (e.g. Enterobacteriaceae and Enterococcus) were negatively correlated with stTPA, but not significantly. Only fecal counts of Clostridium perfringens (pathogenic microorganism) were negatively correlated with stTPA (p = 0.30). |
| Aoyagi et al., Japan, 2019 | Cross-sectional, community-dwelling persons  Age range [65-92] | **NA** | 16S rRNA gene sequencing, V1 - V2 regions | Muscle mass, fat-free mass via BIA | N = 338; ♀ = 198 | Age, sex, BMI, smoking status and alcohol intake  No statement about -biotics intake | No significant associations were found between muscle mass, fat free mass and gut microbiota. |
| Barger et al., United States, 2020 | Cross-sectional, community-dwelling persons  Mean age: 85.0 | **Osteoporosis** | 16S rRNA gene sequencing, V4 region | Whole-body lean mass via DXA  Physical performance via SPPB  HGS via hand dynamometry | N = 111; ♀ = 0 | Age, physical activity score, smoking status and medications intake  No anti- and probiotics intake in the preceding month | Alpha-diversity indices were not, but β-diversity (unweighted Unifrac (p = 0.006), Bray-Curtis (p = 0.05)) were significantly different comparing persons in the highest tertile of fiber intake and muscle mass with those in the lowest tertile. Butyrate-producing bacteria, Ruminococcus (p = 0.01), Lachnospira, (p = 0.04) and Clostridia, (p = 0.002) were ↑ in persons in the highest tertile of fiber intake and muscle mass compare to those in the lowest tertile. |
| Bjørkhaug et al., Norway, 2019 | Cross-sectional, community-dwelling and hospitalized persons  Alcohol use disorder group: Age range [43 – 85]; mean age 64.8  Controls age range [34 – 78]; mean age 58.2 | **Alcohol use disorder (AUD) group versus controls without AUD** | 16S rRNA gene sequencing, V3 - V4 regions | Muscle mass via BIA  HGS via hand dynamometry | N = 42; ♀ = 10 | No adjustment for confounders  No statement about -biotics intake | HGS was significantly lower in persons with alcohol intake disorder (p = 0.019). Persons overconsuming alcohol had ↑ Proteobacteria (p =0.013), ↓ Faecalibacterium (p = 0.004), ↑ Clostridium (p < 0.001), ↑ Sutterella (p = 0.02) and ↑ Holdemania (p = 0.001) compared to controls. No associations between GM and muscle mass, nor HGS were reported. |
| Castro-Mejía et al., Denmark 2020 | Cross-sectional, community-dwelling persons  Mean age 70.2 ± 3.9 | **Not reported** | 16S rRNA gene sequencing, V3 region | Leg soft tissue fat free mass via DXA  Physical performance via 30 s chair stand test and 400 m gait speed test | N = 207; ♀ = 98 | No adjustment for confounders  No antibiotics intake in preceding three months | High fitness group performed significantly more chair stand reps in 30 seconds (p < 0.001 for both genders) and HGS compared to low fitness groups genders. Alpha-diversity did not differ between groups, but β-diversity (weighted Bray-Curtis) was different between groups (p = 0.01). No associations between GM and sarcopenia-defining parameters were reported. |
| Claesson et al., Ireland, 2012 | Cross-sectional, persons living in the community, residing in residential care, in short-term rehabilitation care and in long-term rehabilitation care  Older adults age range [64 – 102]; mean age 78 ± 8  Controls mean age 36 ± 6 | **Long-stay participants had significantly higher CCI** | 16S rRNA gene sequencing, V3 - V4 regions | Calf circumference | N = 178; ♀ = 107 | Age, sex and community-setting  No antibiotics intake in the preceding month | Loss of community-associated microflora were correlated with calf circumference of taken persons together in all four residence locations (p = 0.022 for PC1 and p = 0.047 for PC3) and specifically in persons in a long-stay setting (p = 0.0016). |
| Davis et al., Australia, 2021 | Cross-sectional, community-dwelling persons  Age range [33 – 96]; mean age 64.4 ± 13.5 | **Unspecified multiple ‘gut’ and ‘muscle comorbidities’** | 16S rRNA gene sequencing, V3 – V4 regions | Appendicular lean mass/height ^2^ via DXA  HGS via hand dynamometry  Physical performance via TUG | N = 490 ; ♀ = 490 | Age, smoking, physical activity, Australian Recommended Food Score, intestinal symptoms, potential batch effects and medications that might affect the gut  No statement about -biotics intake | Alpha-diversity was not significantly associated with muscle mass, HGS or TUG-test. Bèta-diversity was not significantly different between persons with low compared to high skeletal muscle index or TUG-test, but approximated significance comparing between persons with low and high HGS (p = 0.081). Butyrate-producing bacteria were associated with higher SMI (β = 0.02,p <0.001)and HGS (β = 0.01, p <0.001) and TUG-test (β = - 0.01, p < 0.01), but association became unsignificant afer adjustment. |
| Dillon et al., United States, 2021 | Case-control, community-dwelling persons  Cases mean age 60.79 ± 7.71  Controls mean age 59.50 ± 6.62  Overall mean age 71.0 | **Persons with HIV versus controls without HIV** | 16S rRNA gene sequencing, V3 – V4 regions | Lean body mass and appendicular lean mass via DXA  HGS via hand dynamometry; leg press, bench press and lateral press lower limb strength  Physical performance via 400 m waling test 10 chair rises and SPPB | N = 36; ♀ = 1 | No adjustment for confounders  No antibiotics intake in the two preceding weeks | Many significant associations were reported between multiple levels of GM-taxa and sarcopenia defining parameters both in controls as in persons with HIV. Directions of reported associations were highly heterogeneous. |
| Grahnemo et al., Norway, 2023 | Cohort, community-dwelling persons  Discovery cohort mean age 60.3 ± 13.9  Replication cohort mean age 53.8 ± 14.0 | **Prevalent Atrial fibrilation, stroke, thyroid disorder, cancer, diabetes (type not specified), asthma, COPD** | Precision Microbiome Profiling | Appendicular and total lean mass via BIA | N = 5196; ♀ = 1879 | Age, gender, height, body fat mass, chronic disease, medication, smoking status and stool consistency  No statement about – biotics intake | Persons that carried the species *Dorea longicatena,* *Coprococcus comes* and *Eubacterium ventriosum* had ↑ appendicular lean mass (p = 5.6 * 10 ^-13^ ) and total lean mass (p = 2.2 * 10 ^-12^) compared to those without any of the three species present. |
| Han et al., Taiwan, 2022 | Cross-sectional, community-dwelling persons  Persons with LM mean age 72.3± 5.4  Persons with NM group mean age 70.0 ± 4.2 | **Prevalent metabolic disorder in both LM and NM groups** | 16S rRNA gene sequencing, V3 – V4 regions | EWGSOP1 | N = 88; ♀ = 60 | Age, BMI, MNA-score, physical activity level  No statement about -biotics intake | No association with sarcopenia was reported. Alpha-diversity was ↓ in persons with LM compared to NM (multiple indices with p < 0.01). GM clustered different comparing between NM and LM (p = 0.037). F/B ratio was ↑ in persons with NM compared to LM (p = 0.043). HGS, scaled SMI and gait speed were correlated (p < 0.05) with multiple GM species. Both positive and negative correlations were reported. |
| Houttu et al., 2021, The Netherlands | Cross-sectional, community-dwelling persons  Overall 51.9 ± 10.7 | **Prevalent diabetes (type not specified) and obesity** | 16S rRNA gene sequencing, V4 region | Calf circumference , thigh circumference  HGS via hand dynamometry | N = 1334; ♀ = 673 | Age, sex, BMI, ethnicity, average intake of fat, grains and carbohydrates  No antibiotics intake in the three preceding months | No significant difference in α- nor β-diversity between persons with and without sarcopenia. Persons with sarcopenia had ↓ *Faecalibacterium prausnitzii* (p = 0.019), *Roseburia inulinivorans* (p = 0.006), *Alistipes shahii* (p = 0.019) compared to persons without sarcopenia. No associations with sarcopenia (-defining) parameters were reported. |
| Hu et al., China, 2022 | Cross-sectional, community-setting not clearly stated  Persons on PD mean age 57.7 ± 8.52  Persons on HD mean age 59.53 ± 10.57 | **ESDR** | 16S rRNA gene sequencing, no info on regions sequenced | Mid-upperarm circumference (MAC) and mid-upperarm muscle circumference (MAMC)  HGS via hand dynamometry | N = 102; ♀ = 45 | No adjustment for confounders  No anti- ,pre-, pro- or synbiotics intake in the three preceding months | In patients with End-Stage Renal Disease, different dialysis treatments (hemodialysis versus peritoneal dialysis) affected the abundance of butyric acid-producing bacteria (*Rosella and Phascorlarctobacterium*) and conditional pathogens (*Escherichia* spp.). Butyric acid producing bacteria were positively correlated with protein energy wasting and negatively with *Escherichia*. |
| Hung et al., Taiwan, 2021 | Cross-sectional, community-dwelling persons  Mean age 63.2 ± 10.2 | **Type II diabetes** | 16S rRNA gene sequencing, no info on regions sequenced | Lean mass/height ^2^ via BIA | N = 179; ♀ = 80 | Age, sex and BMI  No antibiotics intake in the preceding month | In persons with DM type II, higher lean tissue index correlated positively (Spearman’s rho = 0.213; p = 0.004) with the phylum *Firmicutes* and higher F/B ratio (Spearman’s rho = 0.239, p = 0.01). |
| Kang et al., China, 2021 | Case-control, community-dwelling persons  Controls mean age 8.38 ± 5.79  Persons with possible sarcopenia mean age 74.00 ± 6.94  Persons with sarcopenia mean age 76.45 ± 8.58 | **Not stated** | 16S rRNA gene amplicon sequencing, V3 – V4 regions | AWGS2 | N = 87; ♀ = 81 | No adjustment for confounders  No statement about -biotics intake | Microbial diversity was decreased in persons with possible sarcopenia (p <0.05) and sarcopenia (p<0.05) compared to controls. Butyrate producing bacteria *(Lachnospira, Fusicantenibacter, Roseburia, Eubacterium and Lachnoslcostridium)* were decreased in persons with possible sarcopenia and sarcopenia compared to controls (p < 0.05). Lactobacillus was increased in persons with possible sarcopenia and sarcopenia (p < 0.05). |
| Lee et al., South-Korea, 2022 | Case-control, community-dwelling persons  Controls mean age 64.8 ± 3.4  Persons with sarcopenia mean age 66.5 ± 4.6 | **Prevalent diabetes (type not specified), hypertension, dyslipidemia and thyroid disorder in case and control groups. Autoimmune disease was only prevalent in the case group** | 16S rRNA gene amplicon sequencing, V3 – V4 regions | AWGS2 | N = 60, ♀ = 45 | No adjustment for confounders  No statement about -biotics intake | Persons with sarcopenia had increased levels of *Parabacteroides* spp. (p = 0.010) and lower levels of *Prevotella* and *Prevotella copri* (respectively p = 0.021 and 0.018). Persons with and without sarcopenia did not differ significantly regarding α-diversity. |
| Lee et al., Taiwan, 2023 | Cohort, community-dwelling persons  Age range [44.8 – 70.4] | **Liver cirrhosis and sarcopenia versus liver cirrhosis without sarcopenia versus non-cirrhotic, non-sarcopenic controls** | 16S rRNA gene sequencing , V3 – V4 hypervariable region | AWGS2 | N = 89, ♀ = 66 | No adjustment for confounders  No anti-, pre or probiotics intake in the preceding month | Alpha-diversity was not significantly different comparing between controls and persons with cirrhosis and sarcopenia, whereas β-diversity did (p = 0.035). In logistic regression analyses *Dialsiter* (OR = 6.283; p = 0.002), *Ruminococcus* 2 (OR = 4.590; p = 0.031) and *Anaerostipes* (OR = 4.640; p = 0.012) predicted the risk of cirrhotic sarcopenia. |
| Lim et al., South-Korea, 2021 | Cross-sectional, community-dwelling persons  Age range [70 - 90]  Mean age 74.7 ± 4.4 | **Prevalent frailty** | 16S rRNA gene amplicon sequencing, V3 – V4 regions | HGS via a digital dynamometer | N = 176, ♀ = 122 | Age and sex  No antibiotics intake in the three preceding months | No α-diversity markers were associated with HGS. Shannon’s diversity (p = 0.012) and Pielou’s evenness indices associated negatively (p = 0.043) with frailty index score. The latter diversity index was negatively associated with GDS score (p = 0.031)The enterotype *Bacteroides* was prevalent in frail persons (p = 0.0088). |
| Margiotta et al., Italy, 2020 | Cross-sectional, community-dwelling persons  Controls mean age 73.7±7.6  Persons with CKD mean age 80.7±6.2 | **CKD, prevalent frailty** | 16S rRNA gene amplicon sequencing, V3 – V4 regions | Lean tissue mass via BIA | N = 79; ♀ = 32 | No adjustment for confounders  No pro- or antibiotics in the three preceding months | In persons with CKD, lean tissue index was borderline decreased in those with frailty compared to those without (p = 0.05). Indices of α-diversity were not significantly different between persons with or without frailty. *Roseburia* spp. (p < 0.05), *Faecalbacterium* spp. (p < 0.01) and *Prevotella* spp. (p < 0.05) were decreased, whereas Citrobacter (p < 0.01) and *Coprococcus* spp. (p < 0.05)were increased in persons with CKD.  No associations between lean tissue index and GM were reported. |
| Margiotta et al., Italy, 2021 | Cross-sectional, community-setting not clearly stated  Mean age 80.7 ± 6.2 | **CKD** | 16S rRNA gene amplicon sequencing, V3 – V4 regions | EWGSOP2 | N = 64; ♀ = 25 | No adjustment for confounders  No antibiotics in the six preceding months | Persons with sarcopenia had increased levels of *Micrococcaceae, Verrucomicrobiaceae* families (both FDR: 0.012) , and of *Megasphaera* (FDR < 0.001), *Rothia* (FDR = 0.004), *Veillonella* (FDR <0.001), Akkermansia (FDR = 0.008) and *Coprobacillus* genera (FDR = 0.01), but decreased levels of *Acidaminococcus* (FDR < 0.001) and *Gemella* (FDR = 0.03). |
| Palmas et al., Italy, 2021 | Cross-sectional, community-dwelling persons  Controls mean age 49 ± 11  Cases mean age 50 ± 12 | **Obese cases versus non-obese controls. Prevalent hypertension and dyslipidemia in obese persons** | 16S rRNA gene amplicon sequencing, V3 – V4 regions | Muscle mass via DXA | N = 92; ♀ = 80 | Age, sex and smoking status  No antibiotics intake in the three preceding months | Taxa abundant in persons with obesity, *Thermicanaceae* (p = 0.027, Spearman’s rho = - 0.325), *Thermicanus* (p = 0.027, Spearman’s rho = -0.325), *Desulfibrio piger* (p = 0.010, Spearman’s rho = ), were negatively correlated with muscle mass |
| Peng et al., China, 2023 | Cross-sectional, community-setting not clearly stated  Persons with HF mean age 71.76 ± 7.93  Persons with HF and sarcopenia mean age 74.14 ± 8.18; Controls mean age 67.67 ± 9.76 | **HFS cases versus HF only controls versus non-HFS controls** | 16S rRNA gene amplicon sequencing, V3 – V4 regions | AWGS2 | N = 77; ♀= 45 | No adjustment for confounders  No anti- and probiotics intake in the preceding month | Multiple α-diversity indices differed comparing the controls to respectively persons with HF with and without sarcopenia (p < 0.05), however, none of these indices differed comparing between persons with HF with our without sarcopenia. No different clustering (β-diversity) of GM was found comparing persons with HF with and without sarcopenia. The *Synergestetes* phylum was increased in persons with HF and sarcopenia compared to those with HF only (LDA > 2). |
| Picca et al., Italy, 2019 | Cross-sectional, community-dwelling persons  Non-PF&S mean age 73.9 ± 3.2  PF&S mean age 75.5 ± 3.9 | **PF&S** | 16S rRNA gene amplicon sequencing, V3 – V4 regions | SPRINTT operational definition | N = 35; ♀ = 15 | No adjustment for confounders  No statement about -biotics intake | Alpha-diversity did not differ between persons with and without PF&S. *Peptidococcaceae* (p = 0.008), and *Bifidobacteriaceae* (p = 0.013), *Pyramidobacter* (p = 0.043) and *Dialister* (p = 0.028) ↑ , whereas *Slackia* (p < 0.001) and *Eubacterium* ( p = 0.028) ↓ compared to controls. |
| Ponziani et al., Italy, 2021 | Case-control, hospitalized persons  Age range [58.5 – 77.24] | **Cases with liver cirrhosis versus controls without cirrhosis** | 16S rRNA gene amplicon sequencing, V3 – V4 regions | FNIH | N = 100; ♀ = 28 | No adjustment for confounders  No ‘ad hoc’ antibiotics intake | In persons with cirrhotic sarcopenia, ALM/BMI correlated positively with *Slackia* (p = 0.042, correlated coefficient = 0.29). In persons with cirrhotic sarcopenia, *Prevotella* (p < 0.001)*,* *Methanobrevibacter*, (p = 0.01) and *Akkermansia* (p = 0.04) were ↓, and *Eggerthella* ↑ (p = 0.001) compared to persons with non-sarcopenic cirrhosis. Alpha-diversity was ↓ in persons with cirrhotic sarcopenia compared to respectively persons with non-sarcopenic cirrhosis (p = 0.04) and persons with non-cirrhotic sarcopenia (p = 0.002). |
| Soltys et al., Slovakia, 2021 | Cohort, community-dwelling persons  Age range [62.1 – 67.7] | **Not stated** | 16S rRNA gene amplicon sequencing, no info on regions sequenced | Muscle mass %  HGS via hand dynamometry ; knee extension isometric lower limb strength via dynamometry | N = 22; ♀ = 0 | No adjustment for confounders  No antibiotics intake in the two preceding months | Life long athletes (LA) had ↑ HGS compared to controls (p = 0.02). Alpha-diversity did not differ comparing between the LA -group and controls. Both groups clustered different according to GM (p = 0.006). No associations between GM and clinical biomarkers were reported. The Bacteroides/Prevotella ratio was ↓ in the LA group compared to controls (p = 0.03). *Prevotella, Bacteroides, Subdoligranulum, Intestimonas* resulted in an AUC of 0.94 to discriminate between LA and controls. |
| Tavella et al., Italy, 2021 | Cohort, community-dwelling persons  Age range [65 – 79] | **Prevalent Fried defined pre-frailty** | 16S rRNA gene amplicon sequencing, V3 – V4 regions | Skeletal mass index (appendicular lean mass/total body lean mass ratio) via DXA | N = 201; ♀ = 101 | No adjustment for confounders  No statement about -biotics intake | SMI correlated positively with the Christenellaceae R7 group and three Ruminococcus genera and the Eubacterium rectale group, but negatively with Subdoligranulum, Ruminococcus 2 group, Fusicatenibacter and Blautia, but all correlations were non-significant. Based on β-diversity, GM clustered into three distinct groups. (G1 – G3 (p = 0.001). Alpha-diversity was |
| Ticinesi et al., Italy, 2020 | Cross-sectional, community-dwelling persons  Age range [70 - 86] | **Not stated** | Shallow shotgun whole metagenome sequencing | EWGSOP1 | N = 17; ♀ = 14 | No adjustment for confounders  No antibiotics in the preceding month | Alpha-, nor β-diversity differed between persons with and without sarcopenia. In persons with sarcopenia the level of *Faecalibacterium prausnitzii (p = 0.019)*, *Roseburia inuliniovrans* (p = 0.006) and *Alistipes shahii (p = 0.019)* was ↓. No associations between sarcopenia(-defining parameters) or GM were reported. |
| Wang et al., China, 2022 | Cohort, community-dwelling persons  Persons without sarcopenia mean age 62.3 ± 8.5  Persons with sarcopenia mean age 72.2 ± 8.5 | **Not stated** | Shotgun whole metagenome sequencing | AWGS2 | N = 1417; ♀ = 835 | Age, sex, BMI, smoking status, alcoholintake, fracture history, physical activity, frequency of dietary intake of meat/eggs, dairy products  No antibiotics intake in the preceding month | Bèta-diversity was associated with sarcopenia (p = 0.043), α-diversity was not. Persons with sarcopenia had ↑ of *Desulfoviobrio piger* (p = 0.003), *Clostridium sybiosum* (p < 0.001), *Hungatella efflucii* (p = 0.003), *Bacteroides fluxus* ( p = 0.002), *Absiella innocuum* (p = 0.002), *Coprobacter secundus* (p = 0.002) and *Clostridium citroniae* (p = 0.001). |
| Wang et al., China, 2023 | Case-control, community-dwelling persons  Age range [65 – 75] | **Not stated** | Shotgun whole metagenome sequencing | AWGS2 | N = 100; ♀ = 100 | No adjustment for confounders  No anti- pre- or probiotics intake in the 48 preceding hours | Both positive and negative associations were reported between muscle mass and GM at multiple taxonomic levels. In an ROC analysis *Bifidobacterium longum* might be protective for sarcopenia in women ( AUC = 0.647, 95% Ci: 0.539-0.756) with a sensitivity of 53.1% and a specificity of 74.0% |
| Wu et al., Turkey, 2022 | Case-control, community-setting not clearly stated | **Not stated** | 16S rRNA gene amplicon sequencing, V3 - V4 regions | EWGSOP2 | N = 192; ♀ = 105 | No adjustment for confounders  No antibiotic intake in the six preceding months | One α-diversity index was ↓ in persons with sarcopenia compared to controls (p = 0.00586). Bèta-diversity was not reported. Negative associations between sarcopenia and *Lachnospiraceae* (p = 0.038) and *Bacteroides* (p < 0.05) were found. Positive associations between sarcopenia *Coprococcus* (p = 0.032), *Lactobacillus* (p < 0.05) *Enterobacteriaceae* (p < 0.05) and *Clostridiaceae* (p < 0.05). In an ROC curve including *Coprococcus,* *Lachnospiraceae* and P/B ratio resulted in an AUC of 0.61. |
| Xu et al., China, 2021 | Cross-sectional, community-dwelling persons  Age range [70 – 92]  Mean age 80.72 ± 5.75 | **Frailty** | 16S rRNA gene amplicon sequencing, V3 - V4 regions | HGS with hand dynamometry | N = 94; ♀ = 50 | No adjustment for confounders  No antibiotic intake in the preceding month | Persons with frailty had lower HGS compared to persons without frailty (p < 0.001). No GM were significantly associated with HGS. Alpha-diversity was not different between persons with and without frailty, whereas β-diversity was (p = 0.001). At phylum and genus level, different GM were as well ↑ as ↓ in persons with frailty. |
| Yan et al., China, 2023 | Cross-sectional, community-dwelling persons  Age range [60 – 90] | **Prevalent hypertension, diabetes (type unspecified) and coronary heart disease** | 16S rRNA gene amplicon sequencing, no info on regions sequenced | AWGS2 | N = 276; ♀ = 276 | Age, BMI, height, weight, waistline, ALT, ALT/AST and total bilirubin  No intake of anti- or probiotics in the three preceding months | Two α-diversity indices were ↓ in persons with sarcopenia (p < 0.05). Bèta-diversity was different between persons with and without sarcopenia. The F/B ratio was ↓ in persons with sarcopenia (p < 0.05) HGS was negatively correlated with *Actinobacteria* (p < 0.05) and *Bifidobacterium* (p < 0.05), whereas *Agathobacte*r was positively correlated. ASMI was negatively correlated with *Bifidobacterium.* |
| Yamamoto et al., Japan, 2022 | Cross-sectional, community-setting not clearly stated  Age range [50 – 79] | **Chronic liver disease** | 16S rRNA gene amplicon sequencing, V3 – V4 region | Muscle mass area at L3 vertebrae/height ^2^ via CT | N = 69; ♀ = 24 | No adjustment for confounders  No antibiotic intake in the preceding month | Two α-diversity indices were ↓ in persons with low SMI (respectively p = 0.02936 and p = 0.029831). No difference in β-diversity was reported, whereas F/B ratio was ↓ in persons with low SMI (p = 0.0091). No associations with sarcopenia (-defining parameters were reported). |

*BMI: body mass index; ALT: alanine transaminase; AST: aspartate transaminase; LM: Low muscle mass; NM: Normal muscle mass; PD: peritoneal dialysis; HD: hemodialysis, CKD: Chronic Kidney Disease; HF: Heart failure; SHF: Sarcopenia and Heart failure; PF&S: Physical Frailty and Sarcopenia; CT: computed tomography; DXA: Dual X-ray absorptiometry; BIA: Bioelectrical Impedance Analysis; SPPB: Short Physical Performance Battery; HGS: hand grip strength; TUG: Timed Up and Go; GM: gut microbiota; AWGS: Asian Working Group on Sarcopenia 2; EWGSOP2: European Working Group on Sarcopenia in Older People 2; FNIH: Foundation of National Health institutes definition; SPRINTT: sarcopenia and physical frailty in older people: multicomponent treatment strategies; OR/ Odds Ratio; GDS: Geriatric Depression Scale; FDR: False Discovery Rate ; AUC: Area Under the Curve; ROC: Receiver Operating Curve;* ***AUD: Alcohol Use Disorder (AUD);*** ***CCI: Charlson Comorbidity Index; COPD: Chronic Obstructive Pulmonary Disease; ESDR: End Stage Renal Disease***

## Appendix III Abundance of GM taxa and markers of GM diversity in persons with sarcopenia: significant findings

| Ref. | Sarcopenia definition; Prevalence |  | |  | | Abundance of gut bacteria in persons with sarcopenia | | | | Indices of gut bacterial diversity in persons with sarocpenia | |
| --- | --- | --- | --- | --- | --- | --- | --- | --- | --- | --- | --- |
|  |  | Phylum | Class | | Order | | Family | Genus | Species | α-diversity and F/B ratio | β-diversity |
| Han et al. | EWGSOP1; 40.90% | *↓ Firmicutes* | NA | | NA | | *↑Bacteroidaceae, Fusobacteriaceae*  *↓Ruminococcaceae, Prevotellaceae, Akkermansiaceae* | *↑Flavonibacter, Bacteroides, Eggerthella*  ↓*Marvinbryantia, Subdoliranulum, Akkermansia, Ruminococcaceae* UCG-003, *Barnesiella, Odoribacter, Dorea, Alistipes, Peptococcus, Paraprevotella* | *↑Lachnoclostridium phocaeense,*  ↓ *Faecalbacterium prausnitzii, Gabonia massiliensis, Bacteroides eggerthii DSM 20697, Parabacteroides goldsteinii CL02T12C30, Parabacteroides johnsonii CL02T12C29* | ↓Chao1 index, Shannon index, ASV  ↓F/B ratio | Not significantly different between persons with and without sarcopenia or between persons with presarcopenia or confirmed sarcopenia. |
| Kang et al. | AWGS2; 29.88% | *↓Firmicutes* | No significant differences | | No significant differences | | *↑Porphyromonadaceae, Lactobacillaceae* | *↑Lactobacillus*  *↓Lachnospira, Eubacterium, Roseburia, Fusicatenibacter, Lachnoclostridium* | NA | ↓Chao1 index, observed species | Different between controls, older adults with presarcopenia and those with sarcopenia according to least squares discrimination analyses |
| Lee et al., 2023 | AWGS2; 32.58% | *↑Proteobacteria* | *↑*Fusobacteriia | | *↑Fusobacteriales* | | *↑Fusobacteriaceae, Micrococcaceae*  *↓Coriobacteriaceae, Leuconostocaceae,* | *↑Fusobacterium, Rothia*  *↓Lachnospiraceae FCS-020 group, Lachnospiraceae UCG-001, Erysipelotrichaceae UCG-003, Dialister, Ruminococcus 2, Collinsella, Megasphaera, Acidaminococcus, Dorea, Anaerostipes* | *↓ Eubacterium hallii, Eubacterium ventriosum* | No significant differences | Difference between controls and persons with sarcopenia & cirrhosis |
| Margiotta et al., 2021 | EWGSOP2;  28.57% | No significant differences | NA | | NA | | *↑Micrococcaceae, Verrucomicrobiaceae*  *↓Veillonellaceae, Gemellaceae* | ↑*Mehaspehaera, Veillonella, Rohtia, Coprobacillus, Akkermansia*  *↓Acidaminococcus, Gemella* | No significant differences | NA | NA |
| Peng et al., 2023 | AWGS2; 37.66% | *↑Synergistetes*  *↓ Fusobacteria* | ↑ *Synergistales*  ↓ *Fusobacteriales, Rhodobacterales, Methylophilales* | | ↑*Syngergistia* | | *↑ Barnesiellaceae*  ↓*Fusobacteriaceae, mIcrococcaceae, Helicobacteraceae, Rhodobacteraceae, Methylophilaceaea,* | *↓Megamonas, Barnesiella, Helicobacter, Paracoccus, Lactococcus* | NA | ↓Chao1 index, Observed species, Simpson index, Shannon index | Different between controls and respectively HF and SHF, but not comparing between HF and SHF |
| Picca et al., 2019 | Definition of the elaborated SPRINTT project; 51.42% | No significant differences | NA | | NA | | *↑Peptostreptococcaceae, Bifidobacteriaceae* | ↑*Dialister, Pyramidobacter,*  *↓Slakia, Eubacterium* | NA | No significant differences | NA |
| Ponziani et al., 2021 | FNIH; 33.0% | ↓ *Actinobacteria°,*  *Proteobacteria,*  *Euryarchaeota* | NA | | NA | | *↓Veillonellaceae, Methanobacteriaceae,* | ↑*Eggerthella*  *↓Dialister, Prevotella, Methanobrevibacter, Akkermania, Ruminococcus,* | NA | ↓Chao1 index | No significant  differences |
| Ticinesi et al. , 2020 | EWGSOP1;29.41% | NA | NA | | NA | | NA | NA | *↓Faecalibacterium prausnitzii, Roseburia inulinivorans, Alistipes shaii* | No significant differences | No significant differences |
| Wang et al. 2022 | AWGS2; 11.05% | No significant differences | No significant differences | | No significant difference | | No significant differences | ↑*Clostridium, Lawsonibacter* | *↑Desulfoviobrio piger, Clostridium sybiosum, Hungatella efflucii, Bacteroides fluxus, Absiella innocuum, Coprobacter secundus, Clostridium citroniae* | No significant differences | Different between controls and older adults with sarcopenia |
| Wang et al. 2023 | AWGS2; 50% | *↑ Firmicutes, Actinomycetes*  *↓ Bacteroidetes, Proteobacteria* | NA | | NA | | NA | NA | *↑ Lactobacillus fermentum, Eggerthella lenta, Catenibacterium mitsuokai, Fusobacterium periodonticum, Dialister succinatiphilus, Lachnospiraceae bacterium 2158FAA, Collinsella aerofaciens, Subdoligranulum variabile*  *↓Prevotella copri, Bifidobacterium longum, Bacteroides massiliensis, Bacteroides coprocola, Phasolarctobacterium succinatutens, Barnesiella intestinihominis, Clostridium spL250, Bacteroides fluxus, Bacteroidales bacterium ph8, Misuokella multacida, Parasutterella excrementihominis, Burkholderiales bacterium I 147* | ↑ F/B ratio** | NA |
| Wu et al., 2022 | EWGSOP2; 45.83% | *↑Firmicutes, Bacteroidetes*  *↓Proteobacteria* | NA | | *↓ Clostridiales* | | *↓Lachnospiraceae* | *↑Prevotella, Bacteroides, Coprococcus* | NA | ↓Chao1 index | No significant  differences |
| Yan et al., 2023 | AWGS2; 19.20% | *↑Bacteroidetes* | NA | | NA | | NA | *↑Shigella, Bacteroides*  *↓Agathobacter, Dorea* | NA | ↓Chao1 and Ace indices, F/B ratio | Different between older adults with and without sarcopenia |
| Lee et al., 2022 | AWGS2; 45.0% | No significant differences | NA | | NA | | NA | *↓Prevotella, Dialister* | *↑Parabacteroides sp., Anaerotruncus sp., Butyricimona sp., Phascolarctobacterium sp., Ruminococcaceae_*unclassified*,*  *↓Prevotella copri, Dialister* | No significant difference | Different between older adults with and without sarcopenia. |

** Increase of 30% reported in paper, level of significance not clearly stated

EWGSOP: European Working Group on Sarcopenia in Older People; AWGS: Asian Working Group on Sarcopenia; FNIH: Foundation of National Health Institutes the sarcopenia project; SPRINTT: Sarcopenia and Physical fRailty IN older people: multi-componenT Treatment strategies; LM: low muscle mass; NM: Normal Muscle mass; F/B ratio: *Firmicutes/Bacteroidetes* ratio; ASV: Amplicon Sequence Variants

## **Appendix IIIb Abundance of GM taxa and markers of GM diversity in persons with sarcopenia: non-significant findings**

| Ref. | Sarcopenia definition; Prevalence |  | |  | | Abundance of gut bacteria in persons with sarcopenia | | | | Indices of gut bacterial diversity in persons with sarocpenia | |
| --- | --- | --- | --- | --- | --- | --- | --- | --- | --- | --- | --- |
|  |  | Phylum | Class | | Order | | Family | Genus | Species | α-diversity and F/B ratio | β-diversity |
| *Han et al.* | *EWGSOP1; 40.90%* | **NA** | **NA** | | **NA** | | **NA** | **NA** | **NA** | **No non-significant findings specified** | **No non-significant findings specified** |
| Kang et al. | AWGS2; 29.88% | **NA** | **NA** | | **NA** | | **NA** | ***↓Bacteroides*, Faecalibacterium, “unidentified”, Megamonas, Blautia***  ***↑Lachnospira*, Ruminococcaceae UCG, Subdoligranulum*, Ruminococcus 2, Phascolarctobacterium*, Prevotella 9*, Escherichia/Shigella, Bifidobacterium*, Parabacteroides, Alistipes,*** | **NA** | **No non-significant findings specified** | **Non-significantly different according to Unweighted Unifrac analyses** |
| Lee et al., 2023 | AWGS2; 32.58% | **Multiple phyla were non-significantly different, specific levels not stated** | **NA** | | ***↑ Micrococcales*** | | ***↑Enterobacteriaceae, Streptococcaceae,***  ***↓Lachnospiraceae, Ruminococcaceae, Prevotellaceae,*** | ***↑Hungatella, Lachnospiraceae NK4A-136 group***  ***↓Allisonella*** | ***↓ Ruminococcus gavreauii group*** | **↓ Faith’s phylogenetic index, Shannon index** | **No non-significant findings specified** |
| Margiotta et al., 2021 | EWGSOP2;  28.57% | **NA** | **NA** | | **NA** | | ***Multiple family levels were non-significantly different , specific levels not stated.*** | ***Multiple genus levels were non-significantly different, specific levels not stated.*** | **NA** | **NA** | **NA** |
| ^a^Peng et al., 2023 | AWGS2; 37.66% | ***↑ Proteobacteria and Actinobacteria***  ***↓Firmicutes***  ***Bacteroidetes*** | **NA** | | **NA** | | **NA** | ***↑Lactobacillus, Bifidobacterium, Enterococcus***  ***↓Slackia, Clostridia, Faecalbacterium, Blautia, Prevotella (compared to controls), Shigella, Bacteroides, Streptococcus, Gemmiger, Akkermansia (compared to HF)*** | **NA** | **No non-significant findings specified** | **No non-significant findings specified** |
| Picca et al., 2019 | Definition of the elaborated SPRINTT project; 51.42% | ***↑ Synergistetes, Proteobacteria, Euryachaeota, Firmicutes***  ***↓ Bacteroidetes, Verrucomicrobia*** | **NA** | | **NA** | | ***↑ Dethisulfovibrionaceae,***  ***Lactobacillaceae, Clostridiaceae, Pasteruellaceae, Enterococcaceae, Rikenellaceae,***  ***Desulfovibrionaceae, Enterobacteriaceae, Veillonellaceae, Methanobacteriaceae, Lachnospiraceae, Barnesiellaceae,***  ***Dehalobacteriaceae,***  ***Porphyromonadaceae***  ***↓Alcaligenaceae, Carnobacteriaceae, EtOH8, Ruminococcaceae, Bacteroidaceae, Christensenellaceae, Coriobacteriaceae, Mogibacteriaceae, Streptococcaceae, Erysipelotrichaceae, Verrucomicrobiaceae, Paraprevotellaceae, Prevotellaceae, S24-7*** | ***↑ Lactobacillus, Veilonella, Bifidobactrium, Haemophilus, Atopobium, Enterococcus, Mehthanobrevibacter, Phascolarctobacterium, Bilophila, Ruminococcus, Ascillospira, Collinsella, Adlercreutzia, Coprococcsus, Granulicatella, Eggerthella***  ***↓Roseburia, Anerostipes, Parabacteroides, Blautia, Sutterella, Christensenella, Bacteroides, Streptococcus, Lachnospira, Dorea, Faecalibacterium, Anaerotruncus, Paraprevotella, Lachnobacterium, Akkermansia, Prevotella*** | **NA** | **↓Chao1 index** | **NA** |
| Ponziani et al., 2021 | FNIH; 33.0% | ***↓Firmicutes, Cyanobacteria, Bacteroidetes, Actinobacteria, TM7, Verrucomicrobia*** | **NA** | | **NA** | | ***↑ Enterobacteriaceae, Pasteurellaceae, Lactobacillaceae, Micrococcaceae, Rikenellaceae, Streptococcaceae, Carnobacteriaeae, Barnesiellaceae, Veillonellaceae, Lahnospiraceae***  ***↓Peptostreptococcaceae, Mogibacteriaceae, Coriobacteriaceae, Paraprevotellaceae, Ruminococcaceae, Clostridiaceae, Dehaiobacteriaceae, Christensenellaceae, Erysipelotrichaceae, Enterobacteriaceae, Desulfovirbionaceae, Turicibacteriaceae ,*** | ***↑ Phasolarctobacterium, Enterococcus, Lactobacillus, Lachnobacterium, Parabacteroides, Veilonella, Streoptococcus, Klebsiell, Granulicatella, Ruminococcus, Atopobium, Faecalbacterium, Blautia, Haemophilus, Adlercreuzia, Paraprevotella, Bilophila, Lachnospira, Anzerostipes, Rothia, Slackia***  ***↓Roseburia, Coprococcus, Osicllosira, Collinsella, Bacteroides, Dehalobacterium, Dorea, Eubacterium, Bifidobacterium, Ruminococcus, Christenella, Turicibacter, Catenibacterium, Dialister*** | **NA** | **No non -significant differences further specified** | **No non-significant differences were specified** |
| Ticinesi et al. , 2020 | EWGSOP1;29.41% | **NA** | **NA** | | **NA** | | **NA** | **NA** | ***↓ Alistipes onderdonkii, Bacteroides caccae, Bacteroides dorei, Bacteroides vulgatus, Barnesiella intestinihominis, Roseburia intestinalis, Subdoligranulum unknown species***  ***↑ Bacteroides fragilis, Bacteroides uniformis, Bifidobacterium longum, Escherichia coli, Flavonifractor plautii, Parabacteroides distasonis, Parabacteroides merdae, Ruminococcus bromii, Ruminococcus gnavus,*** | **No non-significant differences were specified** | **No non-significant difference further specified** |
| Wang et al. 2022 | AWGS2; 11.05% | **Multiple phyla levels were non-significantly different, specific levels not stated** | **Multiple class levels were non-significantly different, specific levels not stated** | | **Multiple order levels were non-significantly different, specific levels not stated** | | **Multiple family levels were non-significantly different, specific levels not stated.** | **NA** | **NA** | **↑ Shannon index** | **No non-significant differences specified** |
| Wang et al. 2023 | AWGS2; 50% | **NA** | **NA** | | **NA** | | **NA** | **NA** | **NA** | **No non-significant differences specified** | NA |
| Wu et al., 2022 | EWGSOP2; 45.83% | **Multiple phylum levels were non-significantly different, specific levels or directions not stated** | **NA** | | **NA** | | **Multiple family levels were non-significantly different, specific levels or directions not stated** | **Multiple genus levels were non-significantly different, specific levels or directions not stated** | **NA** | **No non-significant differences specified** | **No non-significant differences were specified** |
| Yan et al., 2023 | AWGS2; 19.20% | **NA** | **NA** | | **NA** | | **NA** | **Multiple genus levels were non-significantly different, specific levels or directions not stated** | **NA** | **No non-significant differences specified** | **No non-significant differences specified** |
| Lee et al., 2022 | AWGS2; 45.0% | ***↑Firmicutes***  ***↓Actinobacteria, Verrucomicrobia, Proteobacteria*** | **NA** | | **NA** | | **NA** | **Multiple genus levels were non -significantly different, specific levels or directions not stated** | **Multiple species levels were non-significantly different, specific levels or directions not stated** | **↑ Chao1 index, ↑ Shannon index, ↓ Simpson index** | **No non-significant differences specified** |

*lower in presarcopenic persons as compared to persons with confirmed sarcopenia or persons without sarcopenia

a: not clearly stated whether difference was significant

## Appendix IV Abundance of GM taxa and markers of GM diversity in persons with low muscle mass: signficant findings

| Ref. | Muscle mass measure | Abundance in persons with low muscle mass | | | | | | Diversity findings in persons with low muscle mass | |
| --- | --- | --- | --- | --- | --- | --- | --- | --- | --- |
|  |  | Phylum | Class | Order | Family | Genus | Species | α-diversity and F/B ratio | β-diversity |
| Aoyagi et al., Japan, 2019** | BIA-determined muscle mass and fat-free mass | NA | NA | NA | *↑Fusobacteriaceae*  ↓*Bacillaceae* | No significant differences | NA | NA | NA |
| Barger et al., USA, 2020 | DXA-determined lean mass | ↓*Tenericutes, Lentiphaerea* | ↑ *Betaproteobacteria*;  *↓*RF3, *Lentisphaeria* | ↑*Burkholderiales, Turicibacterales*  *↓*ML615J-28, *Victivallales* | ↑*Porphyromonadaceae*  *Streptococcaceae*  *Alcaligenacae*  *↓Victivallaceae*  *Synergistaceae*  *Dehalobacteriacea* | ↑*Coprobacillus*  *Parabacteroides*  *↓Lachnobacterium*  *Clostridium*  SMB53  *Ruminococcus*  *Odoribacter*  *Lachnospira* | NA | No significant differences | Different between T1T1 and T3T3 groups |
| Han et al., 2022, Taiwan | BIA-determined SMI | ↓*Firmicutes* | NA | NA | ↑*Fusobacteriaceae*  ↓*Ruminococcaceae,*  *Prevotellaceae*  *Akkermansiaceae* | ↑*Flavonifrator*  *Bacteroides*  *Alistipes*  *Eggerthella*  *Sellimonas*  ↓*Marvinbryantia*  *Subdoligranulum*  *Akkermansia*  *Barnesiella*  *Odoribacter*  *Terrisporobacter*  *Paraprevotella*  *Peptococcus*  *Dorea*  *Oxalobacter*  *Leuconostoc* | ↑ *Lachnoclostridium*  *phocaeense*  ↓*Faecalbacterium*  *prausnitzii*  *Parabacteroides*  *goldsteinii CL02T12C30,*  *Parabacteroides johnsonii*  *CL02T12C29*  *Gabonia massiliensis*  *Bacterides eggerthii DSM*  *20697* | ↓F/B ratio  Observed ASV  Shannon index  Chao1 index | Different between persons with low and preserved muscle mass. |
| Yamamoto et al., Japan, 2022 | CT-determined SMI | ↑*Bacteroidetes,*  *Proteobacteria*  *↓Firmicutes* | ↑ Bacteroidia  *↓Clostridia* | ↑*Bacteroidales*  *↓Clostridiales* | ↑ *Bacteroidaceae*  *↓Erysipeolotrichaceae* | ↑ *Bacteroides*  *↓Coprobacillus,*  *Prevotella*  *Catenibacterium*  *Clostridium* | NA | ↓Chao1 index and observed species in L-SMI group  ↓F/B ratio in L-SMI group | No significant differences |
| Hung et al., Taiwan, 2021 | BIA-determined LTI% | *↓Firmicutes* | NA | NA | NA | *↓Bacteroides* | *↑Akkermansia muciniphila* | ↓F/B ratio from highest to lowest LTI tertile. | NA |
| Margiotta et al., Italy, 2020** | BIA-determined LTI% | NA | NA | NA | *↑Coribacteriaceae*  *Mogibacteriaceae* | *↑Lactobacillus*  *Oscillaspira*  *Eggerthella*  *Erwinia*  *Anaerotruncus*  *Actinomyces*  *Coprococcus* | *↑Eubacterium cylindroides,*  *Doreo Unclassified species* | No significant differences | No significant differences |
| Houttu et al., the Netherlands, 2021 | CC  TC | No significant differences | NA | NA | *↑Enterobacteriaceae*  *↓ Lachnospiraceae* | *↑ Escherichia/Shigella*  *Klebsiella*  *↓* *Veillonella* | No significant differences | ↓Shannon’s diversity index,  Richness # | Different between physically active and sedentary persons # |
| Hu et al., China, 2022 | MAC  MAMC | NA | NA | NA | NA | *↑Escherichia*  *↓Roseburia* | NA | No significant differences | NA |
| Davis et al.,  Australia, 2021 | SMI | NA | NA | NA | NA | NA | NA | No significant differences | No significant differences |

DXA: dual X-ray absorptiometry; CT: Computed Tomography; N-SMI: normal skeletal muscle index; LTI: Lean Tissue Index; ALM: Appendicular Lean Mass; T1T1: lowest tertiles of fiber intake and muscle mass; TC: Thigh Circumference; CC: Calf Circumference; MAC: Mid-upperArm Circumference; MAMC: Mid-Upperarm Muscle Circumference

**Difference in muscle/lean mass was borderline not statistically significant between groups (p = 0.05)

# Initially significant difference between groups became unsignificant after multivariate adjustment

## **Appendix IVb: Abundance of GM taxa and GM diversity markers in persons with low muscle mass: non-significant findings**

| Ref. | Muscle mass measure | Abundance in persons with low muscle mass | | | | | | Diversity findings in persons with low muscle mass | |
| --- | --- | --- | --- | --- | --- | --- | --- | --- | --- |
|  |  | Phylum | Class | Order | Family | Genus | Species | α-diversity and F/B ratio | β-diversity |
| Aoyagi et al., Japan, 2019** | BIA-determined muscle mass and fat-free mass | ***NA*** | ***NA*** | ***NA*** | ***↑ Lachnospiraceae, Streptococcaceae, Lactobacillaceae, Clostridiales other, Mogibacteriaceae, Bacteroidaceae, Porphyromonadaceae , Rikenellaceae, Paraprevotellaceae, Barnesiellaceae, Odoribacteraceae, Enterobacteriaceae, Pasteurellaceae,***  ***↓ Ruminococcaceae, Veillonellaceae, Clostridiaceae, Clostridiales unclassified, Enterococcacea, Turicibacteraceae, Christensenellaceae, Bacillaceae, Prevotellaceae, Porphyromonadaceae, S24-7, Bifidobacteriaceae, Coriobacteriaceae, Alcaligenaceae, Verrucomicrobioaceae*** | ***↑Atopobium, Streptococcus, Enterococcus, Staphylococcus, Lactobacillus***  ***↓Bifidobacterium*** | ***↑ Clostridium leptum, Bacteroides fragilis, Clostridium perfringens, Clostridium difficile, Lactobacillus casei, Lactobacillus plantarum, Lactobacillus reuteri, Lactobacillus ruminis, Lactobacllus sakei, Lactobacillus fermentum*** | ***NA*** | ***NA*** |
| Barger et al., USA, 2020 | DXA-determined lean mass | **No non-significant differences specified** | **No non-significant differences specified** | **No non-significant differences specified** | **No non-significant differences specified** | **Multiple genus levels were non-significantly different, specific levels were not stated** | NA | **↓Chao1, ↓ACE, ↓ Shannon, ↓ Simpson, ↓ Fisher** | **No non-significant differences specified.** |
| Han et al., 2022, Taiwan | BIA-determined SMI | **Multiple phyla levels were non-significantly different, specific levels or directions not stated** | **NA** | **NA** | **Multiple family levels were non-significantly different, specific levels not stated.** | **Multiple genus levels were non-significantly different, specific levels not stated** | **Multiple species levels were non-significantly different, specific levels not stated** | **No non-significant differences specified** | **No non-signficant differences specified** |
| Yamamoto et al., Japan, 2022 | CT-determined SMI | **NA** | **NA** | **↑Streptophyta**  **↓ Actinomycetales, Bacillales, Lactobacillales** | **↑ Rikenellaceae, Barnesiellaceae**  **↓Porphyromonadaceae, S24-7, Bacillaceae, Lactobacilleae** | **↑ Actinomyces *, Corynebacterium, Alloscardovia, Enterococcus, Macellibacteroides***  ***Olsenella, Vestibaculum, Barnesiella, Bacillus, Staphylococcus , Melissococcus,***  ***↓ Methanobrevibacter, Methanosphaera, Rothia, Scardovia, Atopobium, Collinsella, Paraeggerthella, Slackia, Eggerthella, Bifidobacterium, Cytophaga, Dysgonomonas Parabacteroides, Alistipes, Odoribacter, Paraprevotella, Lactobacillus, Christensenella*** | **NA** | **No non-significant differences specified** | **No non-significant differences specified** |
| Hung et al., Taiwan, 2021 | BIA-determined LTI% | ***↑ Bacteroidetes*** | **NA** | **NA** | **NA** | ***↓Bifidobacterium*** | ***↓ Clostridium leptum , Escherichia coli, Faecalibacterium prausnitzii*** | **No non-significant differences specified** | NA |
| Margiotta et al., Italy, 2020** | BIA-determined LTI% | **NA** | **NA** | **NA** | **Multiple family levels were non-significantly altered, specific levels or directions not stated** | **Multiple genus levels were non-significantly altered, specific levels or directions were not stated** | **Multiple species levels were non-significantly different, specific levels or directions were not stated** | **Non-significant differences not specified** | **Non-significant differences not specified** |
| Houttu et al., the Netherlands, 2021 | CC  TC | ***↑ Verrucomicrobia, Tenericutes, Firmicutes,***  ***↓ Bacteroidetes, Proteobacteria, Actinobacteria*** | **NA** | **NA** | ***↑Rikenellaceae, Barneseiellaceae, Tannerellaceae, Peptococcaceae, Peptostreptococcaceae, Akkermansiaceae, Desulfovibrioceae, Eggerthellaceae, Streptococcaceae, Ruminococcaceae,***  ***↓ Bifidobacteriaceae, Coriobacteriaceae, Veillonellaceae,*** | ***↑ Akkermansia, Desulfovibrio, Phascolarctobacterium, Streptococcus, Faecalibacterium, Ruminococcus 2, Ruminiclostridium 5, Intestinimonas,***  ***↓ Anaerostipes, Bilophila, Sutterella, Dorea*** | ***↑ Alistipes obesi, Coprococcus 2 eutactus, Akkermansia muciniphila, Slackia isoflavoniconvertens, Faecalibacterium CM04.06, Faecalibacterium prausnitzii, Ruminococcus bromii,***  ***↓Bacteroides caccae, Odoribacter splanchnicus, Bacteroides vulgatus, Parabacteroides merdae, Roseburia inulinivorans, Roseburia intestinalis, Fusicatenibacter saccarivorans, Coprococcus 3 comes, Dorea formicigenerans, Bilophila wadswortia, Collinsella aerofaciens, Rumincoccus 1 bicirculan,*** | **No non-significant differences specified** | **No non-significant differences specified** |
| Hu et al., China, 2022 | MAC  MAMC | NA | NA | NA | NA | **No non-significantly differences in genera levels specified** | NA | **Non- significant differences not specified** | NA |
| Davis et al.,  Australia, 2021 | SMI | **NA** | **NA** | **NA** | **NA** | **NA** | **NA** | **No non-significant differences specified** | **No non-significant differences specified** |

## Appendix V Abundance of GM taxa and GM diversity markers in persons with low muscle strength: significant findings

| Ref. | Muscle strength measure | Diversity findings in persons with low muscle strength | | | | | | | |
| --- | --- | --- | --- | --- | --- | --- | --- | --- | --- |
|  |  | Phylum | Class | Order | Family | Genus | Species | α-diversity and F/B ratio | β-diversity |
| Bjørkhaug et al., Norway, 2019 | HGS | *↑Proteobacteria*  *↓Actinobacteria, Coriobacteria,* | *↓ Clostridia* | *↑ Enterobacteriales, Desulfovibrionales, Actinomycetales*  *↓Coriobacteriales,* | *↑ Lachnospiraceae, Enterobacteriaceae, Alcaligenaceae, Micrococcaceae, Tissierellaceae*  *↓Staphococcacea, Coriobacterioceae, Prevotellaceae,* | *↑ Clostridium, Holdemania, Suterella, Escherichia, Klebsiella, Acidaminococcus, Morganella, Bulleidia, Ellin6513, Rothia, Epulopiscium, Megamonas,*  *↓Faecalbacterium, Odoribacter, Mitsuokella, RF3, Oribacterium, Succinatimonas, Adlercreutzia, Staphylococcus, Clostridia, Collinsella,* | NA | No significant differences | Different between controls and persons with alcohol use disorder |
| Castro-Mejia et al., Denmark, 2020 | 30 second CST | NA | NA | No significant difference | No significant difference | No significant difference | No significant difference | *↓ O*bserved species in low fitness phenotype group | Different between high and low fitness phenotype groups |
| Xu et al., China, 2021 | HGS | *↑Proteobacteria*  *Actinobacteria Verrucomicrobia*  *Synergistetes*  *↓Firmicutes* | *↑ Verrucomicrobia, Actinobacteria*  *↓ Negativicutes* | *↑Enterobacteriales, Bifidobacteriales, Synergistales,*  *↓Pasteurellales, Selenomonadales* | *↑ Rikenellaceae, Synergistaceae, Lactobacillaceae, Bifidobacteriaceae, Verrucomicrobiaceae, Prophyromonadaceae, Enterobacteriaceae*  *↓Veillonellaceae, Prevotellaceae, Acidamonacoccaceae, Pasteurellaceae* | *↑Escherichia/Shigella*  *Bifidobacterium*  *Lactobacillus*  *Parabacteroides*  *Akkermansia*  *Klebsiella*  *Alistipes*  *Enterobacteriaceae_unknown*  *↓Faecalibacterium*  *Prevotella*  *Blautia, Streptococcus, Clostridiales unknown* | NA | No significant differences | Different between frail and non-frail groups |
| Han et al. Taiwan, 2022 | HGS | No significant difference | NA | NA | NA | No significant difference | No significant difference | No significant differences | No significant differences |
| Houttu et al., The Netherlands, 2021 | HGS | No significant difference | NA | NA | ↑*Enterobacteriaceae*  *↓Lachnospiraceae* | *↑Escherichia/Shigella*  *Klebsiella, Prevotella 2 #, Erysipelatoclastridium#*  *↓Veillonella* | *↑Roseburia hominis#* | ↓Shannon’s index ##, Richness ## in sedentary group | Different between PA and sedentary groups ## |
| Soltys et al., Slovakia, 2021 | HGS | No significant difference | *↑ Cytophagia* | No significant difference | ↑*Bacteroidaceae*  *↓ Incerae Sedis XI,* | *↑ Bacteroides*  *Pseudobutyrivibrio*  *Anaerosporobacter*    *↓ Porphyromona,*  *Prevotella, Intestinimonas, Marvinbryantia, Vallitalea, Subdoligranulum* | NA | No significant differences | Different between LA and CON groups |
| Hu et al., China, 2022 | HGS | NA | NA | NA | NA | *↑ Escherichia*  *↓Roseburia* | NA | No significant differences | NA |
| Davis et al., Australia, 2021 | HGS | NA | NA | NA | NA | NA | NA | No significant differences | No significant differences |

HGS: Hand grip Strength; CON: controls; CST: Chair Stand Test; PA: physically active; LA: Life-long athletes

# became non-significant after correction for multiple testing

##became non-significant after adjustment for confounders

## **Appendix Vb: Abundance of GM taxa and GM diversity markers in persons with low muscle strength: non-significant findings**

| Ref. | Muscle strength measure | Diversity findings in persons with low muscle strength | | | | | | | |
| --- | --- | --- | --- | --- | --- | --- | --- | --- | --- |
|  |  | Phylum | Class | Order | Family | Genus | Species | α-diversity and F/B ratio | β-diversity |
| Bjørkhaug et al., Norway, 2019 | HGS | **Non non-significant alterations were specified** | **Non non-significant alterations were specified** | **Non non-significant alterations were specified** | **Non non-significant alterations were specified** | **Non non-significant alterations were specified** | **NA** | **Non-signficant findings not specified** | **No non-signficant differences specified** |
| Castro-Mejia et al., Denmark, 2020 | 30 second CST | **NA** | **NA** | **NA** | **NA** | **NA** | ***Non-significant differences without stating specific levels or directions: in Mogibacterium PAC 001609 PAC001611, Ruminococcaceae PAC 000748 PAC002351, Turicibacter CP013476, Barnesiella intestinihominis, Anerotruncus colihominis, Christensenellaca minuta, Caproiciproducens PAC 001616, Caproiciproducens PAC001617, Coprobacter_other, Ruminococcaceae PAC000748 PAC001145, Oscillibacter_Other, Christensenellaceae PAC00115 PAC001444, Saccharimonas PAC001343, Mollicutes PAC001057 PAC001057 PAC001108 Other, Mollicutes PAC001057 PAC001057 PAC001274 , Escherichia Other, Harryflintia acetispora, Caproiciproducens other, Bifidobacterium aolescentis, Ruminococcaceae PAC000661 Other between groups*** |  |  |
| Xu et al., China, 2021 | HGS | **Multiple phyla levels were non-significantly altered, specific levels or directions not stated** | **Multiple class levels were non-significantly altered, specific levels or directions not stated** | **Multiple order levels were non-significantly altered, specific levels or directions not stated** | ***↑ Ruminococcaceae_unclassified*** | ***↓ Ruminoccus, Lachnospiraceae_unclassified, Lachnospiraceae incerae sedis*** | ***NA*** | **Multiple α-diversity indices were non-significantly altered, but no specific exact values or directions were stated** | **No non-significant differences specified** |
| Han et al. Taiwan, 2022 | HGS | No significant difference | NA | NA | NA | **No non-significant differences were specified** | **No non-significant differences were specified** | **Multiple α-diversity indices were non-significantly altered, but no specific exact values or directions were stated** **specified** | **Non-significant β-diversity differences, but no exact values were stated** |
| Houttu et al., The Netherlands, 2021 | HGS | ***↑ Proteobacteria, Actinobacteria,***  ***↓ Tenericutes , Firmicutes, Verrucomicrobia,*** | **NA** | **NA** | ***↑ Bifidobacteriaceae, Coriobacteriaceae, Veillonellaceae, Clostridiaceae,***  ***↓Rikenellaceae, Barnesiellaceae, Tannerellaceae, Peptococcaceae, Peptostreptococcaceae, Akkermansiaceae, Desulfovirbionaceae, Eggerthellaceae, Streptococcaceae, Christensenellaceae, Ruminococcaceae,*** | ***↑ Anaerostipes, Bilophila, Sutterella, Bifidobacterium , Collinsella, Dialister, Dorea***  ***↓ Akkermansia, Desulfovibrio, Phascolarctobacterium, Streptococcus, Faecalibacterium, Ruminococcus, Ruminiclostridium, Intestinimonas,*** | ***↑Bacteroides caccae, Odoribacter splanchnicus, Roseburia inulinivorans, Roseburia intestinalis , Fusicatenibacter saccharivorans, Coprococcus 3 comes, Dorea formicigenerans, Bilophila wadsworthia, Collinsella aerofaciens, Ruminococcus bicirculans***  ***↓Alistipes obesi, Bacteroides vulgatus, Parabacteroides merdae, Coprococcus 2 eutactus Akkermansia muciniphila, Slackia isoflavoniconvertens, Faecalibacterium CM04.06, Faecalibacterium prausnitzii,*** | **No non- significant differences specified** | **No non-signficant differences specified** |
| Soltys et al., Slovakia, 2021 | HGS | **Phylum level bacterial taxa were determined, but not specified** | **Class level bacterial taxa were determined, but not specified** | **Multiple order levels were non-significantly altered, specific levels or directions not stated** | ***↓ Ruminococcaceae*** | ***↑ Anaerovorax*** | **NA** | **No non- significant differences specified** | **No non-significant differences specified** |
| Hu et al., China, 2022 | HGS | NA | NA | NA | NA | **No other changes in genus level bacteria were specified** | NA | **Non-significant differences were not further specified** | NA |
| Davis et al., Australia, 2021 | HGS | NA | NA | NA | NA | NA | NA | **No non--significant differences specified** | **No non- significant differences specified** |

## Appendix VI Abundance of GM taxa and GM diversity markers in persons with low physical performance: significant findings

| Ref. | Physical performance measures | Abundance of bacterial taxa in persons low physical performance | | | | | Diversity findings in persons with low physical performance | |
| --- | --- | --- | --- | --- | --- | --- | --- | --- |
|  |  | Phylum | Class | Order | Family | Genus | α-diversity and F/B ratio | β-diversity |
| Barger et al., United States, 2020 | SPPB | *↓Tenericutes, Lentisphaerae* | *↑Betaproteobacteria*  *↓*RF3, *Lentisphaeria* | ↑*Burkholderiales, Turicibacterales*  *↓*ML615J-28, *Victivallales* | *↑Porphyromonadaceae, Streptococcaceae, Alcaligenaceae*  *↓Victivallaceae, Synergistaceae, Dehalobacteriacea* | *↑Coprobacillus, Parabacteroides*  *↓Lachnobacterium, Clostridium, SMB53, Ruminococcus, Odoribacter, Lachnospira* | No significant differences | Different between T1T1 and T3T3 |
| Dillon et al. United States, 2021** | 400 m walking test | No significant difference | NA | NA | ↓*Lachnospiraceae* | ↓*Alistipes* | No significant differences | NA |
| Picca et al., Italy 2019 | SPPB | No significant difference | NA | NA | ↑*Peptostreptococcaceae, Bifidobacteriaceae* | ↑ *Dialister, Pyramidobacter, Eggerthella*  ↓*Eubacterium, Slackia* | No significant differences | NA |
| Davis et al. Australia, 2021 | TUG-test | NA | NA | NA | NA | NA | No significant differences | Different between persons with low and high TUG test scores# |

Short physical performance battery (SPPB); Timed Up and Go (TUG)

** Difference in physical performance between groups was borderline not statistically significant (p = 0.05)

# Result became non-significant after adjustment for multiple confounders.

## **Appendix VIb Abundance of GM taxa and GM diversity markers in persons with low physical performance: non-significant findings**

| Ref. | Physical performance measures | Abundance of bacterial taxa in persons low physical performance | | | | | Diversity findings in persons with low physical performance | |
| --- | --- | --- | --- | --- | --- | --- | --- | --- |
|  |  | Phylum | Class | Order | Family | Genus | α-diversity and F/B ratio | β-diversity |
| Barger et al., United States, 2020 | SPPB | **No non-significant differences were specified** | **No non-significant differences were specified** | **No non-significant differences were specified** | **No non-significant differences were specified** | **Multiple genera were non-significantly different, specific values not stated.** | **Multiple α-diversity indices were non-significantly different, specific levels were not stated** | **No non-significant differences were specified** |
| Dillon et al. United States, 2021** | 400 m walking test | **No non-significant differences specified** | NA | NA | **No non-significant differences specified** | **No non-significant differences specified** | **No non-significant differences were further specified** | NA |
| Picca et al., Italy 2019 | SPPB | ***↑ Synergistetes, Proteobacteria, Euryachaeota, Firmicutes***  ***↓ Bacteroidetes, Verrucomicrobia*** | NA | NA | ***↑ Dethisulfovibrionaceae,***  ***Lactobacillaceae, Clostridiaceae, Pasteruellaceae, Enterococcaceae, Rikenellaceae,***  ***Desulfovibrionaceae, Enterobacteriaceae, Veillonellaceae, Methanobacteriaceae, Lachnospiraceae, Barnesiellaceae,***  ***Dehalobacteriaceae,***  ***Porphyromonadaceae***  ***↓Alcaligenaceae, Carnobacteriaceae, EtOH8, Ruminococcaceae, Bacteroidaceae, Christensenellaceae, Coriobacteriaceae, Mogibacteriaceae, Streptococcaceae, Erysipelotrichaceae, Verrucomicrobiaceae, Paraprevotellaceae, Prevotellaceae, S24-7*** | ***↑ Lactobacillus, Veilonella, Bifidobactrium, Haemophilus, Atopobium, Enterococcus, Mehthanobrevibacter, Phascolarctobacterium, Bilophila, Ruminococcus, Ascillospira, Collinsella, Adlercreutzia, Coprococcsus, Granulicatella, Eggerthella***  ***↓Roseburia, Anerostipes, Parabacteroides, Blautia, Sutterella, Christensenella, Bacteroides, Streptococcus, Lachnospira, Dorea, Faecalibacterium, Anaerotruncus, Paraprevotella, Lachnobacterium, Akkermansia, Prevotella*** | ***↓Chao1 index*** | NA |
| Davis et al. Australia, 2021 | TUG-test | NA | NA | NA | NA | NA | **No non-significant differences specified** | **No non-significant differences specified** |

## Appendix VII Quality assessment of included studies according to the Newcastle-Ottawa Scale (NOS)

| Study design | Selection  Maximum score: 4 | Comparability  Maximum score: 2 | Outcome ascertainment  Maximum score: | Total score |
| --- | --- | --- | --- | --- |
| Cross-sectional studies | | | | |
| Aoyagi et al. 2019 | 🟊🟊🟊 |  | 🟊 | 🟊🟊🟊🟊 |
| Barger et al. 2020 | 🟊🟊 |  | 🟊 | 🟊🟊🟊 |
| Bjorkhaug et al. 2019 | 🟊🟊 | 🟊🟊 | 🟊 | 🟊🟊🟊🟊🟊 |
| Castro-Mejia et al. 2019 | 🟊🟊🟊 |  | 🟊 | 🟊🟊🟊🟊 |
| Claesson et al. 2012 |  |  | 🟊 | 🟊 |
| Han et al. 2022 | 🟊🟊 | 🟊🟊 |  | 🟊🟊🟊🟊 |
| Hung et al. 2021 |  |  | 🟊 | 🟊 |
| Hu et al. 2022 | 🟊🟊 |  | 🟊 | 🟊🟊🟊 |
| Houttu et al. 2021 | 🟊 |  | 🟊 | 🟊🟊 |
| Lim et al. 2020 | 🟊 | 🟊🟊 | 🟊 | 🟊🟊🟊🟊 |
| Margiotta et al. 2021 | 🟊 | 🟊🟊 | 🟊 | 🟊🟊🟊🟊 |
| Margiotta et al. 2020 | 🟊 | 🟊🟊 | 🟊 | 🟊🟊🟊🟊 |
| Palmas et al. 2021 | 🟊 | 🟊🟊 | 🟊 | 🟊🟊🟊🟊 |
| Picca et al. 2019 | 🟊🟊 | 🟊🟊 | 🟊 | 🟊🟊🟊🟊🟊 |
| Ticinesi et al. 2020 | 🟊 | 🟊🟊 | 🟊 | 🟊🟊🟊🟊 |
| Xu et al. 2021 | 🟊🟊 | 🟊🟊 | 🟊 | 🟊🟊 |
| Yamamoto et al. 2022 | 🟊 | 🟊🟊 | 🟊 | 🟊🟊🟊🟊 |
| Yan et al. 2023 | 🟊🟊 | 🟊🟊 | 🟊🟊 | 🟊🟊🟊🟊🟊🟊 |
| Peng et al. 2023 | 🟊 |  | 🟊 | 🟊🟊 |
| Davis et al. 2023 | 🟊 | 🟊🟊 | 🟊🟊 | 🟊🟊🟊🟊🟊 |
| Case-Control studies | | | | |
| Dillon et al. 2019 | 🟊🟊 | 🟊🟊 | 🟊 | 🟊🟊🟊🟊🟊 |
| Kang et al. 2021 | 🟊🟊 | 🟊 | 🟊 | 🟊🟊🟊🟊 |
| Lee et al. 2022 | 🟊 |  | 🟊🟊 | 🟊🟊🟊 |
| Ponziani et al. 2021 | 🟊 | 🟊🟊 | 🟊🟊 | 🟊🟊🟊🟊🟊 |
| Wu et al. 2022 |  | 🟊 | 🟊 | 🟊🟊🟊 |
| Wang et al. 2023 | 🟊🟊🟊🟊 |  | 🟊 | 🟊🟊🟊🟊🟊 |
| Cohort studies | | | | |
| Akashi et al. 2019 | 🟊 |  | 🟊 | 🟊🟊 |
| Soltys et al. 2021 |  | 🟊🟊 |  | 🟊🟊 |
| Tavella et al. 2021 | 🟊 |  | 🟊 | 🟊🟊 |
| Wang et al. 2022 | 🟊🟊🟊 | 🟊🟊 |  | 🟊🟊🟊🟊🟊 |
| Grahnemo et al. 2022 | 🟊🟊🟊 | 🟊🟊 | 🟊 | 🟊🟊🟊🟊🟊🟊 |
| Lee et al. 2023 | 🟊🟊 |  | 🟊🟊 | 🟊🟊🟊🟊 |

## **Table S1: Associations between GM taxa and sarcopenia as a construct: non-significant findings**

| Author, Country, Year | Sample size (N), sarcopenia definition, prevalence | Positive associations | Negative associations | Adjustment for confounders |
| --- | --- | --- | --- | --- |
| *Margiotta et al., Italy, 2021* | ***N = 64, EWGSOP2, 28.57%*** | Family: ***Alcaligenaceae (r : 0.032)***  ***Bacteroidaceae (r : 0.034)***  ***Bifidobacteriaceae (r : 0.069)***  ***Christensenellaceae (r : 0.090)***  ***Clostridiaceae (r : 0.044)***  ***Enterobacteriaceae (r : 0.0191)***  ***Micrococcaceae (r : 0.075)***  ***Paraprevotellaceae (r : 0.042)***  ***Pasteurellaceae (r : 0.099)***  ***Peptostreptococcaceae***  ***( r : 0.076)***  ***Porphyromonadaceae (r : 0.009)***  ***Rikenellaceae (r : 0.073)***  ***Staphylococcaceae (r: 0.072)***  ***Verrucomicrobiaceae (r : 0.230)***  **Genus: *Akkermansia (r : 0.230)***  ***Alistipes (r : 0.139)***  ***Blautia (r : 0.004)***  ***Clostridium (r : 0.062)***  ***Coprobacillus (r : 0.108)***  ***Dialister (r : 0.017)***  ***Eggerthella (r : 0.177)***  ***Oscillspira (r : 0.004)***  ***Paraprevotella (r : 0.042)***  ***Ruminococcus (r : 0.091)***  ***Suterella (r : 0.032)*** | **Family: *Actinomycetaceae***  ***(r: -0.062)***  ***Barnesiellaceae***  ***(r: -0.194)***  ***Coriobacteriaceae***  ***(r: -0.012)***  ***Enterococcaceaee***  ***(r: -0.050)***  ***Lactobacillaceae***  ***(r: -0.133)***  ***Prevotellaceae (r: -0.060)***  ***Streptococcaceae***  ***(r: -0.008)***  ***Turicibacteraceae***  ***(r: -0.080)***  ***Veillonellaceae (r: -0.208)***  **Genus: *Actinomyces (r : -0.062)***  ***Catenibacterium (r : -0.152)***  ***Collinsella (r : -0.141)***  ***Desulfovibrio (r : -0.024)***  ***Dorea (r : -0.089)***  ***Enterococcus (r : -0.050)***  ***Faecalibacterium (r: -0.026)***  ***Lachnobacterium r: -0.078)***  ***Lactobacillus r : -0.133)***  ***Prevotella (r : -0.060)***  ***SMB53 (r : -0.016)***  ***Streptococcus (r : -0.008)***  ***Turicibacter (r : -0.080)*** | Not adjusted |
| Lee et al., South-Korea, 2022 | N : 60, AWGS2, 45.00% | Genus: ***Ruminococcaceae***  ***_unclassified***  ***Butyricimonas***  ***Parabacteroides***  ***Phascolarctobacterium*** | Genus: ***Dialister*** | Not adjusted |
| ^*^Wu et al., Turkey, 2022 | N : 192, EWGSOP2, 45.83% | **Genus: *Coprococcus*** | **Family: *Lachnospiraceae*** | Not adjusted |
| Wang et al., China, 2022 | N : 1417, AWGS2, 11.05% | **No non-significant associations specified** | **No non-significant associations specified** | Adjusted for age, sex, BMI, smoking status and alcohol intake, fracture history, physical activity, frequency of dietary intake of meat/eggs, dairy products and vegetables. |
| Lee et al., Taiwan, 2023 | N : 89, AWGS2, 32.58% | **Genus : *Fusobacterium (OR : 1.625 ;***  ***CI [0.656 – 4.027])***  ***Rothia (OR : 0.164 ; CI***  ***[0.762 – 4.943])***  **Species : *Eubacterium hallii (OR : 2.403;***  ***CI [0.891 – 6.841])*** |  | Not adjusted |
| Wang et al., China, 2023 | N : 100, EWGSOP2, 50% | **No non-significant associations specified** | **No non-significant associations specified** | Not adjusted |

## **Table S2: Associations between GM taxa and muscle mass: non-significant findings**

| Authors, Country, Year | Sample size (N), Muscle mass measure | GM-taxa | | | Diversity markers | Adjustment for confounders |
| --- | --- | --- | --- | --- | --- | --- |
|  |  | Positive association | | Negative association |  |  |
| Claesson et al., Ireland, 2012 (30) | N = 178,  CC | NA | NA | | **α -diversity**  NA  **β-diversity**  ***No non-significant associations specified*** | Age, sex and community-setting |
| Akashi et al., Japan, 2019 (50) | N = 127,  stTPA | **Genus: *Atopobium***  ***Bifidobacterium***  ***Prevotella***  ***Staphylococcus***  ***Pseudomonas***  **Species: *Clostridium coccoides***  ***Clostridium leptum***  ***Bacteroides fragilis*** | **Family: *Enterobacteriaceae***  **Genus: *Lactobacillus***  ***Enterococcus*** | | **α-diversity**  NA  **β-diversity**  NA | Not adjusted |
| Dillon et al., USA, 2020 (47) | N = 36,  ALM, LBM | **Phylum: *Firmicutes (β_ALM HIV+_:***  ***0.0056; β _ALM HIV -_: 0.0089;***  ***β_LBM HIV+_: 0.0090; β _LBM HIV-_***  ***: 0.0210)***  ***Bacteroidetes***  ***(β_ALM HIV+_: 0.001)***  ***Proteobacteria (β _ALM HIV -_:***  ***0.0846; β _LBM HIV-_: 0.1403)***  ***Actinobacteria (β _ALM HIV +_:***  ***0.0082; β _LBM HIV+_: 0.0059;***  ***β_LBM HIV-_: 0.0810)***  ***Fusobacteria (β _ALM HIV+_:***  ***0.0604; β _LBM HIV+_: 0.1274;***  ***β _ALM HIV-_: 0.0466; β_LBM HIV-_ :***  ***0.0727)***  **Family: *Lachnospiraceae (β_ALM HIV+_:***  ***0.0328, β _LBM HIV+_: 0.0748)***  ***Ruminococcaceae (β_ALM HIV +_:***  ***0.003; β_ALM HIV-_: 0.0240;***  ***β_LBM HIV-_: 0.0519)***  ***Prevotellaceae (β_ALM HIV+_:***  ***0.0330; β_LBM HIV+_: 0.0457;***  ***β_ALM HIV-:_ 0.0227; β_LBM HIV_ -:***  ***0.0278)***  ***Coriobacteriaceae (β_ALM HIV+_:***  ***0.0474; β_LBM HIV+_: 0.0584;***  ***β_LBM HIV-_: 0.1092)***  ***Rikenellaceae (β_ALM HIV+:_***  ***0.2496; β_LBM HIV+_: 0.3645)***  ***Erysipelotrichaceae (β_ALM HIV+_:***  ***0.0684; β_LBM HIV+_: 0.1531)***  ***Enterobacteriaceae (β_ALM HIV-_:***  ***0.0250; β_LBM HIV-_: 0.0497)***  **Genus: *Bifidobacterium (β_LBM HIV-_:***  ***0.236)***  ***Collinsellla (β_ALM HIV+_: 0.030;***  ***β_LBM HIV+_: 0.058; β_ALM HIV-_: 0.007;***  ***β_LBM HIV-_: 0.166)***  ***Alistipes (β_ALM HIV+_: 0.115;***  ***β_LBM HIV+_: 0.337)***  ***Prevotella (β_ALM HIV+_: 0.035;***  ***β _LBM HIV+_: 0.049;***  ***β_ALM HIV-_: 0.023;***  ***β_LBM HIV-_: 0.027)***  ***Anaerotruncus (β_ALM HIV+_: 0.163;***  ***β_LBM HIV+_: 0.231; β_ALM HIV-_: 0.525:***  ***β_LBM HIV-_: 1.055)***  ***Blautia (β_LBM HIV+_: 0.030;***  ***β_ALM HIV-_: 0.042; β_LBM HIV-_: 0.096)***  ***Catenibacterium (β_ALM HIV+_:***  ***0.076; β_LBM HIV+_: 0.154)***  ***Coprococcus (β_ALM HIV+_: 0.556; β***  ***_ALM HIV-_: 0.355; β_LBM HIV-_: 0.579)***  ***Dialister (β ALM HIV-: 0.040;***  ***β LBM HIV-: 0.086)***  ***Dorea (β_ALM HIV+_: 0.172;***  ***β_LBM HIV+_: 0.297; β_LBM HIV-_: 0.844;***  ***β_ALM HIV-_: 0.512)***  ***Faecalibacterium (β_ALM HIV+_:***  ***0.014; β_LBM HIV+_: 0.093; β_LBM HIV-_:***  ***0.049)***  ***Meganomonas (β_ALM HIV+_: 0.193;***  ***β_LBM HIV+_: 0.321; β_ALM HIV-_: 0.010)***  ***Phascolarctobacterium***  ***(β_ALM HIV+_: 0.104)***  ***Pseudobutyrivibrio (β_ALM HIV+_:***  ***0.010; β_LBM HIV+_: 0.036)***  ***Roseburia (β_ALM HIV-_: 0.034;***  ***β_LBM HIV-_: 0.112)***  ***Ruminococcus (β_ALM HIV-_: 0.005;***  ***β_LMB HIV-_: 0.039)***  ***Fusobacterium (β_ALM HIV+_: 0.065;***  ***β_LBM HIV+_: 0.136; β_ALM HIV-_: 0.047;***  ***β_LBM HIV-_: 0.072)***  ***Escherichia (β_ALM HIV-_: 0.025;***  ***β_LBM HIV-_: 0.050***  ***Succinivibrio (β_ALM HIV-_: 0.467;***  ***β_LBM HIV-_: 0.774)***  ***Sutterella (β_ALM HIV+_: 0.074;***  ***β_LBM HIV+_: 0.207; β_ALM HIV-_: 0.105;***  ***β_LBM HIV-_: 0.086)***  ***Butyrivibrio (β_ALM HIV+_: 0.58;***  ***β_LBM HIV+_: 0.80)*** | **Phylum:*Bacteroidetes (β_LBM HIV+_:***  ***-0.006; β _ALM HIV-_ :***  ***-0.014; β _LBM HIV-_:***  ***-0.041)***  ***Proteobacteria***  ***(β _LBM HIV+_: -0.104)***  ***Actinobacteria***  ***(β_ALM HIV-_: - 0.004)***  **Family: *Lachnospiraceae (β _ALM HIV -_: -0.004; β _LBM HIV-_ : -0.003)***  ***Ruminococcaceae***  ***(β _LBM HIV+_: -0.014)***  ***Bacteroidaceae (β _LBM HIV+_:***  ***-0.066; β _ALM HIV-_ : -0.022;***  ***β _LBM HIV-_: -0.046)***  ***Porphyrodomonaceae***  ***(β_ALM HIV+_: -0.022;***  ***β_LBM HIV+_: -0.032;***  ***β_ALM HIV-_ : -0.056;***  ***β_LBM HIV-_: -0.112)***  ***Veillonellaceae (β_ALM HIV+_:***  ***-0.024; β_LBM HIV+_: -0.041;***  ***β _ALM HIV-_: -0.003; β_LBM HIV-_:***  ***-0.031)***  ***Coriobacteriaceae (β_ALM HIV-_:***  ***-0.006)***  ***Rikenellaceae (β_ALM HIV-_:***  ***-0.008; β_LBM HIV-_: -0.049)***  ***Enterobacteriaceae***  ***(β _ALM HIV+_: -0.090;***  ***β_LBM HIV+_: -0.163)***  **Genus: *Bifidobacterium (β_ALM HIV+_:***  ***-0.564; β_LBM HIV+_: -1.14;***  ***β_ALM HIV-_: -0.007)***  ***Alistipes (β_ALM HIV-_: -0.030;***  ***β_LBM HIV-_: -0.083)***  ***Bacteroides (β_LBM HIV+_:***  ***-0.0066; β_ALM HIV-_: -0.022;***  ***β_LBM HIV-_: -0.046)***  ***Parabacteroides (β_ALM HIV+_:***  ***-0.024; β _LBM HIV+_: -0.037;***  ***β_ALM HIV-_: -0.057; β_LBM HIV-_:***  ***-0.111)***  **Anaerostipes (β_ALM HIV+_:**  ***-0.04; β_LBM HIV+_: -0.06;***  ***β_ALM HIV-_: -0.04; β_LBM HIV-_:***  ***-0.12)***  ***Blautia (β_ALM HIV+_: -0.025)***  ***Dialister (β_ALM HIV+_: -0.033;***  ***β_LBM HIV+_: -0.085)***  ***Faecalibacterium (β_ALM HIV-_:***  ***-0.005)***  ***Meganomonas (β_LBM HIV-_:***  ***-0.008)***  ***Megasphaera (β_ALM HIV+_:***  ***-0.108; β_LBM HIV+_: -0.175;***  ***β_ALM HIV-_: -0.064; β_LBM HIV-_:***  ***-0.213)***  ***Phascolarctobacterium***  ***(β_LBM HIV+_: -0.377; β_ALM HIV-_:***  ***-0.055; β_LBM HIV-_: -0.191)***  ***Pseudobutyrivibrio (β_ALM HIV-_:***  ***-0.003; β_LBM HIV-_: -0.014)***  ***Roseburia (β_ALM HIV+_: -0.147;***  ***β_LBM HIV+_: -0.396)***  ***Ruminococcus (β_ALM HIV+_:***  ***-0.653; β_LBM HIV+_: -1.29)***  ***Subdoligranulum (β_ALM HIV-_:***  ***-0.009; β_LBM HIV-_: -0.059)***  ***Succinivibrio (β_ALM HIV+_:***  ***-0.042; β_LBM HIV+_: -0.021)*** | | **α-diversity**  NA  **β-diversity**  NA | Not adjusted |
| Houttu et al., The Netherlands, 2021 (31) | N = 1334,  CC, TC | **Phylum*:Lentisphaerae***  ***(r_CC_: 0.013)***  ***Verrucomicrobia***  ***(r_CC_ : 0.028; r_TC_ : 0.054)***  ***Proteobacteria***  ***(r_CC_ : 0.0097)***  ***Tenericutes (r_CC_ : 0.0052)***  ***Firmicutes***  ***(r_CC_ : 0.029; r_TC_ : 0.052)***  ***Actinobacteria (r_TC_ : 0.046)***  **Family*: Rikenellaceae***  ***(r_CC_ : 0.0096)***  ***Barnesiellaceae***  ***(r_CC_ : 0.0072)***  ***Prevotellaceae***  ***(r_CC_ : 0.0034)***  ***Bacteroidaceae***  ***(r_CC_ : 0.0032)***  ***Peptococcaceae***  ***(r_CC_ : 0.018; r_TC_ : 0.049)***  ***Akkermansiaceae***  ***(r_CC_ :0.026; r_TC_ : 0.051)***  ***Desulfovibrionaceae***  ***(r_CC_ : 0.046)***  ***Enterobacteriaceae***  ***(r_CC_ : 0.035)***  ***Clostridiaceae (r_CC_ : 0.074;***  ***r_TC_ : 0.038)***  ***Ruminococcaceae***  ***(r_CC_ : 0.063; r_TC_ : 0.052)***  ***Lactobacillaceae (r_TC_ : 0.018)***  ***Bifidobacteriaceae (r_TC_ : 0.033)***  **Genus: *Prevotella 2 (r_CC :_ 0.0040)***  ***Bacteroides (r_CC_ : 0.0033)***  ***Roseburia (r_CC_ : 0.027)***  ***Lachnospira***  ***(r_CC_ : 0.075; r_TC_ : 0.044)***  ***Desulfovibrio***  ***(r_CC_ : 0.046; r_TC_ : 0.042)***  ***Romboutsia***  ***(r_CC_ : 0.054; r_TC_ : 0.033)***  ***Faecalibacterium***  ***(r_CC_ : 0.019)***  ***Ruminiclostridium***  ***(r_CC_ : 0.037; r_TC_ : 0.084)***  ***Anearostipes (r_TC_ : 0.007)***  ***Peptococcus (r_TC_ : 0.029)***  ***Akkermansia (r_TC_ : 0.051)***  ***Lactobacillus (r_TC_ : 0.018)***  **Species: *Barnesiella intestinihominis***  ***(r_CC_ : 0.031, r_TC_: 0.012)***  ***Parabacteroides distasonis***  ***(r_CC_: 0.013; )***  ***Parabacteroides merdae***  ***(r_CC_ :0.031)***  ***Bacteroides massiliensis***  ***(r_CC_ : 0.019)***  ***Roseburia inulinivorans***  ***(r_CC_ : 0.0063)***  ***Roseburia hominis***  ***(r_CC_ : 0.043; r_TC_ : 0.023)***  ***Roseburia intestinalis***  ***(r_CC_ : 0.022)***  ***Dorea formicigenerans***  ***(r_CC_ : 0.00089; r_TC_ : 0.02)***  ***Coprococcus 1 catus***  ***(r_CC_ : 0.018)***  ***Akkermansia muciniphila***  ***(r_CC_ : 0.017; r_TC_: 0.039)***  ***Faecalibacterium prausnitzii***  ***(r_CC_; 0.07) r_TC_ : 0.017)***  ***Anaerostipes hadrus***  ***(r_TC_ : 0.0067)***  ***Lactobacillus ruminis***  ***(r_TC_ : 0.025)***  ***Slackia isoflavoniconvertens***  ***(r_TC_: 0.044)*** | **Phylum*: Bacteroidetes***  ***(r_CC_ : -0.017)***  ***Actinobacteria***  ***(r_CC_ : -0.026)***  ***Proteobacteria***  ***(r_TC_ : -0.005)***  **Family*: Lachnospiraceae***  ***(r_CC_ : -0.012;***  ***r_TC_ : -0.005)***  ***Bifidobacteriaceae***  ***(r_CC_ : -0.02)***  ***Lactobacillaceae***  ***(r_CC_ : -0.011)***  ***Rikenellaceae***  ***(r_TC_ : -0.0021)***  ***Barnesiellaceae***  ***(r_TC_ : -0.012)***  ***Prevotellaceae***  ***(r_TC_ : -0.022)***  ***Enterobacteriaceae***  ***(r_TC_ : -0.019)***  **Genus: *Prevotella 2 (r_TC_ : - 0.062)***  ***Prevotella 7***  ***(r_C_c : -0.005;r_TC_ : -0.0063)***  ***Prevotella 9***  ***(r_CC_ : -0.011;r_TC_ : -0.021)***  ***Coprococcus 3***  ***(r_CC_ -0.021;r_TC_ : -0.0018)***  ***Anaerostipes***  ***(r_CC_ : -0.05)***  ***Peptococcus (r_CC_ : -0.016)***  ***Bifidobacterium***  ***(r_CC_ : -0.021)***  ***Slackia (r_CC_ : -0.017)***  ***Lactobacillus***  ***(r_CC_ : -0.011)***  ***Streptococcus***  ***(r_CC_ : -0.00018;***  ***r_TC_ – 0.0835)***  ***Bacteroides***  ***(r_TC_ : -0.050)***  ***Faecalibacterium***  ***(r_TC_ : -0.00092)***  **Species: *Parabacteroides***  ***distansonis (r_TC:_ -0.023)***  ***Bacteroides***  ***massiliensis***  ***(r_TC_ : -0.0036)***  ***Prevotella 9 copri***  ***(r_CC_ : -0.032;r_TC_ :-0.038)***  ***Coprococcus 3***  ***comes (r_CC_; r_TC_)***  ***Anaerostipes hadrus***  ***(_rCC_  : -0.057)***  ***Bifidobacterium***  ***bifidum (r_CC_)***  ***Collinsella***  ***aerofaciens (r_CC_)***  ***Lactobacillus ruminis***  ***(r_CC_ : -0.019)***  ***Roseburia***  ***inulinivorans (r_TC_ : -0.0025)***  ***Parabacteroides***  ***merdae (r_TC :_ -0.014)***  ***Roseburia intestinalis***  ***(r_TC_ : -0.035)***  ***Coprococcus 1 catus***  ***(r_TC_ : -0.0025)***  ***Slackia isoflavoniconvertens***  ***(r_CC_: -0.024)*** | | **α-diversity**  **Shannon index β_TC +_ Richness (β_TC_: +)**  **Faith’s phylogenetic diversity index (β_TC_ +)**  **Inverse Simpson index (β_CC_  +)**  **β-diversity**  NA | Age, sex, BMI, ethnicity, average fat- , grain and carbohydrate intake |
| Hung et al., Taiwan, 2021 (33) | N = 179,  LTI% | **Genus: *Bifidobacterium***  **Species*: Clostridium leptum***  ***Escherichia coli*** | **Phylum: *Bacteroidetes***  **Genus: *Bacteroides***  **Species: *Akkermansia***  ***muciniphila***  ***Faecalibacterium***  ***prausnitzii*** | | **F/B-ratio**  **(r : 0.239)**  **β-diversity**  NA | Age, sex, BMI |
| Kang et al., China, 2021 (14) | N = 87,  ASMI | **Genus: *Lachnoclostridium***  ***Megasphaera***  ***Megamonas***  ***Alistipes***  ***Fusicatenibacter***  ***Faecalibacterium***  ***Blautia***  ***Prevotella 9***  ***Lachnospira***  ***Ruminococcus***  ***Subdoligranulum*** | **Genus: *Parabacteroides***  ***Bacteroides***  ***Escherichia/***  ***Shigella*** | | **α -diversity**  NA  **β-diversity**  NA | Not adjusted |
| Palmas et al., Italy, 2021 (37) | N = 92,  DXA-determined muscle mass | **No non-significant associations were specified** | **No non-significant associations were specified** | | **α-diversity**  NA  **β-diversity**  NA | Age, sex, smoking |
| Ponziani et al., Italy, 2021 (46) | N = 100,  ALM | **Family: *Prevotellaceae (r : 0.234)***  ***Ruminococcaceae (r : 0.166)***  ***Porphyromonadaceae***  ***(r : 0.082)***  ***Methanobacteriaceae***  ***(r : 0.0706)***  **Genus: *Prevotella (r : 0.023)***  ***Christensenella (r : 0.212)***  ***Ruminococcus (r : 0.189)***  ***Atopobium (r : 0.100)***  ***Lachnobacterium (r : 0.091)***  ***Dialister (r : 0.023)***  ***Streptococcus (r : 0.0106)*** | **Family: *Rikenellaceae***  ***(r : -0.091)***  ***Barnesiellaceae***  ***(r : -0.077)***  ***Verrucomicrobiaceae***  ***(r : -0.043)***  ***Desulfovibrionaceae***  ***(r : -0.023)***  ***Veillonellaceae***  ***(r : -0.0059)***  **Genus: *Eggerthella***  ***( r : -0.260)***  ***Klebsiella (r : -0.153)***  ***Akkermansia***  ***(r : -0.0409)*** | | **α -diversity**  NA  **β-diversity**  NA | Not adjusted |
| Tavella et al., Italy, 2021 (52)* | N = 201,  SMI | Genus: ***Bacteroides*** | Genus: ***Streptococcus***  ***Alistipes*** | | **α-diversity**  NA  **β-diversity**  NA | Not adjusted |
| Han et al., Taiwan, 2022 (13) | N = 88,  SMI | **Genus: *Akkermansia sp.***  ***Streptococcus sp.***  **Species: Bacteroides eggerthii**  **Parabacteroides**  **johnsonii CL02T12C29**  **Eubacterium**  **coprostanoligenes** | **Genus: *Fusobacterium sp.***  ***Ruminoclostridium***  ***5 sp.***  ***Enterococcus sp.***  **Species*: Clostridium***  ***innocuum***  ***Ruminococcus***  ***gnavus group sp.***  ***Bifidobacterium***  ***longum*** | | **α -diversity**  ***No non-significant findings were specified***  **β-diversity**  NA | Age, BMI, MNA-score, physical activity level |
| Hu et al., China, 2022 (32) | N = 102,  MAC, MAMC, TSF | **Genus*: Roseburia (r_TSF, HD+:_ 0.016;***  ***r_TSF, PD+_ 0.012; r_MAMC, PD+_ 0.251)***  ***Phascolarctobacterium***  ***(r_TSE, HD+_ :0.014; r_TSE, PD+_***  ***0.024; r_MAC, HD+_ 0.137;***  ***r_MAMC, HD+_ 0.123)***  ***Coprococcus (r_TSF, HD+:_ 0.099;***  ***r_TSF_, _PD+_ 0.024)*** | **Genus: *Escherichia (r_TSF,HD+:_ -0.013; r_TSF, PD+_ -0.094; r_MAC, HD+_ -0.28; r_MAMC, HD+_ -0.265 )*** | | **α-diversity**  **Shannon index (r_TSF_, _HD+_ 0.117; r_TSF, PD+_ 0.033),**  **Simpson index (r_TSE, HD+_ -0.11; rTSE, PD+ -0.030; rMAC_, HD+_ -0.278)**  **β-diversity**  NA | Not adjusted |
| Davis et al., Australia, 2023 (44) | N = 490,  SMI | **No non-significant associations specified** | **No non-significant associations specified** | | **α -diversity**  **Shannon (β: -0.026), Observed species (β : -0.001)**  **β-diversity**  **Unweighted UniFrac distance, Weighted UniFrac distance** | Age, smoking, physical activity, intestinal symptoms, batch effects, medications |
| Grahnemo et al., Norway, 2023 (55) | N = 5196,  ALM | **Species*: Bifidobacterium adolescentis***  ***(β: 0.09)***  ***Bifidobacterium bifidum***  ***(β: 0.01)***  ***Bifidobacterium catenulatum***  ***(β:0.11)***  ***Bifidobacterium longum***  ***(β: 0.07)***  ***Collinsella aerofaciens***  ***(β: 0.27)***  ***Bacteroides caccae (β: 017)***  ***Bacteroides cellulosilyticus***  ***(β: 0.15)***  ***Bacteroides eggerthii (β:0.08)***  ***Bacteroides finegoldii (β: 0.03)***  ***Bacteroides ovatus (β: 0.10)***  ***Bacteroides thetaiotaomicron***  ***(β: 0.16)***  ***Bacteroides xylanisolvens***  ***(β: 0.18)***  ***Phocaeicola dorei (β :0.18)***  ***Phocaeicola massiliensis***  ***(β : 0.19)***  ***Phocaeicola vulgatus (β : 0.06)***  ***Barnesiella intestinihominis***  ***(β : 0.11)***  ***Parabacteroides distasonis***  ***(β : 0.16)***  ***Parabacteroides merdae***  ***(β :0.20)***  ***Prevotella copri (β :0.18)***  ***Alistipes finegoldii (β :0.18)***  ***Alistipes putredinis (β :0.17)***  ***Streptococcus thermophilus***  ***(β : 0.15)***  ***Anaerobutyricum hallii (β : 0.09)***  ***Dorea formicigenerans (β: 0.24)***  ***Roseburia intestinalis (β: 0.17)***  ***Faecalibacterium prausnitzii***  ***(β: 0.27)***  ***Agathobacter rectalis (β: 0.26)***  ***Lachnospira eligens (β: 0.14)***  ***Sutterella wadsworthensis***  ***(β: 0.17)*** | **Species*: Bifidobacterium pseudocatenulatum***  ***(β: -0.10)***  ***Christensenella***  ***minuta (β: -0.25)***  ***Clostridium***  ***leptum (β: -0.22)***  ***Clostridium***  ***scindens (β: -0.31)***  ***Blautia***  ***hydrogenotrophica***  ***(β : -0.22)***  ***Roseburia hominis***  ***(β: -0.05)***  ***Escherichia coli***  ***(β: -0.03)***  ***Akkermansia***  ***muciniphila (β: -0.04)***  ***Methanobrevibacter***  ***smithii (β:-0.09)*** | | **α-diversity**  NA  **β-diversity**  NA | Age, sex, height, fat mass, chronic disease, medication, smoking, stool consistency **& Bonferroni correction** |
| Yan et al., China, 2023 (42) | N = 276,  ASMI, CC | **Phylum: *Proteobacteria (r_ASMI_)***  ***Desulfobacterota (r_CC_)***  **Genus: *Ruminococcus torques***  ***group (r_ASMI & CC_)***  ***Clostridia (r_ASMI_)***  ***Dorea (r_ASMI_)***  ***Fusicatenibacter (r_ASMI_)***  ***Ruminococcus (r_ASMI_)***  ***Collinsella (r_ASMI & CC_)***  ***Enterobacter(r_ASMI_)***  ***Faecalibacterium (r_ASMI & CC_)***  ***Shigella (r_ASMI_)***  ***Ruminococcus (r_CC_)***  ***Agathobacter (r_CC_)***  ***Streptococcus (r_CC_)***  ***Eubacterium hallii group (r_CC_)***  ***Enterobacter (r_CC_)*** | **Phylum: *Firmicutes (r_ASMI_)***  ***Proteobacteria (r_CC_)***  ***Verrucomicrobia***  ***(r_CC_)***  **Genus: *Bacteroides***  ***(r_ASMI & CC_)***  ***UCG014 (r_ASMI & CC_)***  ***Clostridium senso***  ***stricto 1(r_ASMI_)***  ***Agathobacter (r_ASMI_)***  ***Streptococcus***  ***(r_ASMI_)***  ***Romboutsia (r_ASMI_)***  ***Eubacterium hallii***  ***group (r_ASMI_)***  ***Blautia (r_ASMI & CC_)***  ***Clostridia (r_CC_)***  ***Fusicatenibacter***  ***(r_CC_)***  ***Bifidobacterium***  ***(r_CC_)***  ***Shigella (r_CC_)***  ***Dorea (r_CC_)*** | | **α-diversity**  **F/B ratio (+ r_ASMI & CC_)**  **β-diversity**  NA | Age, BMI, height, weight, waistline, ALT, ALT/AST, total bilirubin |
| Wang et al., China, 2023 | N = 100,  BIA-derived skeletal muscle mass | **Species*: Clostridium sp. L250***  ***Parasutterella***  ***exrementihominis***  ***Burkholderiales bacterium***  ***1147***  ***Phascolarctobacterium***  ***succinatutens***  ***Prevotella copri***  ***Dialister succinatiphilus*** | **Genus: *Collinsella***  ***Blautia***  ***Ruminococcus***  **Species: *candidate division***  ***TM7 single cell***  ***isolate TM7b***  ***Fusobacterium***  ***periodonticum***  ***Bifidobacterium***  ***longum***  ***Lactobacillus***  ***fermentum***  ***Lachnospiraceae***  ***bacterium***  ***2158FAA***  ***Catenibacterium***  ***mitsuokai*** | |  |  |

##

## **Table S3: Associations between GM taxa and muscle strength: non-significant findings**

| Authors, Country, Year | Sample size,  Muscle strength measure | GM taxa | | Diversity markers | Adjustment for confounders |
| --- | --- | --- | --- | --- | --- |
|  |  | **Positive associations** | **Negative associations** |  |  |
| Dillon et al., USA, 2020 (47) | N = 36,  CST time, HGS, BP, LPD and LP | **Phylum*: Firmicutes (β_CST HIV-:_***  ***0.0738; β_BP HIV+_: 0.0027;***  ***β_LPD HIV+_: 0.0029)***  ***Bacteroidetes (β_CST HIV+_:***  ***0.0921; β_HGS HIV+_: 0.0233;***  ***β_HGS HIV-:_ 0.0793; β_LP HIV+/-_:***  ***0.0094)***  ***Proteobacteria (β_CST HIV-_:***  ***0.0546; β_HGS HIV+_: 0.2007;***  ***β_BP, LP HIV-_: 0.0065; β_LP HIV+_:***  ***0.0144)***  ***Actinobacteria (β_CST HIV-_:***  ***0.2204; β_HGS HIV+_: 0.1380;***  ***β_BP HIV+_: 0.0064; β_LPD HIV-_:***  ***0.0065)***  **Family*:Lachnospiraceae (β _CST HIV-_:***  ***0.0883; β BP HIV+: 0.0054;***  ***β _LPD HIV+_: 0.0026)***  ***Ruminococcaceae (β _CST HIV-_***  ***: 0.1155; β_HGS HIV+_: 0.1642;***  ***β_HGS HIV-_: 0.2466; β_BP HIV+_:***  ***0.002; β_LPD HIV+_: 0.0025)***  ***Bacteroidaceae (β_CSR HIV+_:***  ***0.1928; β_BP HIV-_: 0.0044;***  ***β_LP HIV+_: 0.0135; β_LP HIV-_:***  ***0.0036)***  ***Prevotellaceae (β_HGS HIV+_:***  ***0.1913; β_HGS HIV-_: 0.2771;***  ***β_BP HIV+_: 0.0005; β_BP HIV-_:***  ***00016; β_LP HIV+_: 0.0015;***  ***β LP HIV-: 0.001)***  ***Porphyromonaceae***  ***(β _CST HIV+_: 0.1252; β_CST HIV-:_***  ***0.0105; β_HGS HIV-_; 0.0145;***  ***β_BP HIV-_: 0.0128; β_LPD HIV-_:***  ***0.0078; β_LP HIV+_: 0.0211;***  ***β_LP HIV-_: 0.0207)***  ***Veillonellaceae (β_CST HIV+_:***  ***0.0533; β_HGS HIV-_: 0.3407;***  ***β_BP HIV-_: 0.0002; β_LPD HIV+_:***  ***0.0071; β_LP HIV-_: 0.0337)***  ***Coriobacteriaceae (β_CST HIV-_:***  ***0.2700; β_HGS HIV+_: 0.1547;***  ***β_BP HIV+_: 0.0056)***  ***Rikenellaceae (β_CST HIV+_:***  ***1.071; β_HGS HIV_+: 1.473;***  ***β_LP HIV+_: 0.0107)***  ***Erysipelotrichaceae***  ***(β_HGS HIV-_: 1.614; β_BP HIV+_:***  ***0.0271; β_BP HIV-_: 0.0230;***  ***β LPD HIV+: 0.0237;***  ***β LP HIV+: 0.0016)***  ***Enterobacteriaceae***  ***(β_CST HIV+_: 0.4353; β_CST HIV-_:***  ***0.2877; β_HGS HIV+_: 0.58;***  ***β_BP HIV+_: 0.001; β_LPD HIV-_:***  ***0.0015; β_LP HIV+_: 0.0056;***  ***β_LP HIV-_: 0.0250)***  **Genus: *Bifidobacterium (β _CST HIV+_:***  ***0.667; β _CST HIV-_: 0.742;***  ***β _BP HIV+_: 0.015; β _LPD HIV+_:***  ***0.040)***  ***Collinsella (β _CST HIV-_: 0.434;***  ***β _HGS HIV+_: 0.268;***  ***β _BP HIV+_: 0.001)***  ***Alistipes (β _HGS HIV+_: 0.311;***  ***β _LP HIV+_: 0.074)***  ***Bacteroides (β _CST HIV+_:***  ***0.193; β_BP HIV-_: 0.004;***  ***β_LP HIV+_: 0.013; β_LP HIV-_:0.004)***  ***Parabacteroides (β_CST HIV+_:***  ***0.120; β_CST HIV-_: 0.099;***  ***β_HGS HIV-_: 0.041; β_BP HIV-_:***  ***0.013; β_LBP HIV-_: 0.007;***  ***β_LP HIV-_: 0.002; β_LP HIV+_:***  ***0.020)***  ***Prevotella (β_HGS HIV+_: 0.202;***  ***β_HGS HIV-_: 0.294; β_BP HIV+_:***  ***0.001; β_BP HIV-_: 0.002;***  ***β_LP HIV+_: 0.002)***  ***Anaerostipes (β_LBD HIV-_: 0.01;***  ***β LP HIV-: 0.02)***  ***Anaerotruncus (β_CST HIV-_:***  ***1.897; β_HGS HIV+_: 2.563;***  ***β_HGS HIV-_: 3.558;***  ***β_BP HIV+_: 0.003; β _LBD HIV+_:***  ***0.037; β_LP HIV+_: 0.005)***  ***Blautia (β_CST HIV-_: 0.506;***  ***β_BP HIV+_: 0.025; β_LPD HIV+_:***  ***0.018; β_LPD HIV-_: 0.002)***  ***Catenibacterium (β _HGS HIV-_:***  ***1.314; β_BP HIV_+: 0.032;***  ***β_BP HIV-_: 0.017; β_LPD HIV+_:***  ***0.031; β_LP HIV+_: 0.004;***  ***β_LP HIV-_: 0.002)***  ***Coprococcus (β_CST HIV_-: 0.041;***  ***β_HGS HIV-_: 1.628; β_BP HIV+_:***  ***0.008; β_LPD HIV+_: 0.036;***  ***β_LP HIV-_: 0.072)***  ***Dialister (β_HGS HIV-_: 0.872;***  ***β_LP HIV-_: 0.033; β_LPD HIV+_: 0.007;***  ***β_LPD HIV-_: 0.001; β_LP HIV-_: 0.101)***  ***Dorea (β_HGS HIV -_: 2.020;***  ***β_BP HIV+_: 0.046; β_LPD HIV+_: 0.029;***  ***β_LP HIV-_ : 0.087)***  ***Faecalibacterium (β_CST HIV+_:***  ***0.350; β_CST HIV-_: 0.432; β_HGS HIV-_:***  ***0.176)***  ***Megamonas (β_BP HIV+_: 0.011;***  ***β_LPD HIV-_: 0.021; β_LP HIV+_: 0.026;***  ***β_LP HIV-_: 0.002)***  ***Megasphaera (β_CST HIV+_:***  ***0.573; β_HGS HIV-_: 2.165;***  ***β_BP HIV+_: 0.001; β_LPD HIV+_: 0.007;***  ***β_LPD HIV-_: 0.004; β_LP HIV+_: 0.017;***  ***β_LP HIV-_: 0.078)***  ***Phascolarctobacterium***  ***(β_HGS HIV+_****: 1.608;* ***β_HGS HIV-_****: 0.789;*  ***β_BP HIV+_: 0.047; β_BP HIV-_: 0.013;***  ***β_LPD HIV+_: 0.031; β_LPD HIV-_: 0.033)***  ***Roseburia (β_CST HIV-:_ 0.131;***  ***β_HGS HIV+_: 0.673; β_BP HIV_+: 0.047;***  ***β_BP HIV-_: 0.016; β_LPD HIV+_: 0.063;***  ***β_LPD HIV-_: 0.008)***  ***Pseudobutyrivibrio (β_CST HIV-_:***  ***0.052; β_HGS HIV+_: 0.033; β_LPD HIV-_:***  ***0.009; β_LP HIV-_: 0.002)***  ***Ruminococcus (β_CST HIV+_: 0.891;***  ***β_CST HIV-_: 0.105; β_HGS HIV-_: 1.178;***  ***β_BP HIV+_: 0.124; β _BP HIV-_: 0.012;***  ***β_LPD HIV+_: 0.158; β _LPD HIV-_: 0.013;***  ***β_LP HIV-_: 0.032)***  ***Subdoligranulum (β _CST HIV+_:***  ***1.141; β_CST HIV-_: 0.120; β_HGS HIV-_:***  ***0.688; β _BP HIV-_: 0.007; β_LPD HIV-_:***  ***0.013; β_LP HIV-_: 0.008)***  ***Escherichia (β _CST HIV-_: 0.296;***  ***β _LPD HIV-_: 0.002; β _LP HIV+_: 0.217;***  ***β_LP HIV-_: 0.021)***  ***Succinovibrio (β _CST HIV+_: 0.369;***  ***β _HGS HIV-_: 0.552; β _BP HIV-_: 0.056;***  ***β _LP HIV+_: 0.064)***  ***Sutterella (β_HGS HIV+_: 0.584;***  ***β_HGS HIV+_: 0.494; β_BP HIV-_: 0.083;***  ***β_LPD HIV+_: 0.008; β_LPD HIV-_: 0.070;***  ***β_LP HIV+_: 0.155)***  ***Butyrivibrio (β_HGS HIV+_: 0.10;***  ***β_HGS HIV-_: 2.98; β_BP HIV+_: 0.34;***  ***β_BP HIV-_: 0.19; β_LPD HIV+_: 0.55;***  ***β_LP HIV+_: 0.28)*** | **Phylum*: Firmicutes (β_CST HIV+_: -0.169;***  ***β_HGS HIV+_: -0.075; β_HGS HIV-_:***  ***-0.019; β_BP HIV-_: -0.004;***  ***β_LPD HIV-_: -0.003; β_LP HIV-_:***  ***-0.007; β_LP HIV+_: -0.008)***  ***Bacteroidetes (β_CST HIV-_:***  ***-0.104; β_BP HIV+_: -0.002;***  ***β_LPD HIV+_: -0.001)***  ***Proteobacteria***  ***(β_HGS HIV-_: -0.272)***  ***Actinobacteria (β_CST HIV+_:***  ***-0.641; β_HGS HIV-_: -0.269;***  ***Β_LPD HIV+_: -0.009; β_LP HIV+_:***  ***-0.056; β_LP HIV-_: -0.024)***  ***Fusobacterium (β_CST HIV+_:***  ***-0.196; β_CST HIV-_: -0.398;***  ***β_HGS HIV+_: -0.052; β_HGS HIV-_:***  ***-0.474; β_BP HIV+_: -0.004;***  ***β_BP HIV-_: -0.003; β_LPD HIV+_:***  ***-0.010; β_LPD HIV-_: -0.017;***  ***β_LP HIV+_: -0.007;***  ***β_LP HIV-_: -0.025)***  **Family*: Lachnospiraceae (β_HGS HIV+_:***  ***-0.285; β_HGS HIV-_: -0.221;***  ***β_BP HIV-_: -0.005; β_LPD HIV-_: -0.001;***  ***β_LP HIV+/-_: -0.013)***  ***Ruminococcaceae (β_CST HIV+_:***  ***-0.161; β_BP HIV-_: -0.001; β_LPD HIV-_:***  ***-0.003; β_LP HIV+_: -0.016)***  ***Bacteroidaceae (β_CST HIV-_:***  ***-0.036; β_HGS HIV+_: -0.175;***  ***β_HGS HIV-_: -0.119; β_BP HIV+_: -0.003)***  ***Prevotellaceae (β_CST HIV+_:***  ***-0.060; β_CST HIV-_: -0.088;***  ***β_LPD HIV-_: -0.001)***  ***Porphyrodomonaceae***  ***(β_HGS HIV+_: -0.239; β _BP HIV+_:***  ***-0.006; β_LBP HIV+_: -0.001)***  ***Veillonellaceae (β_CST HIV-_:***  ***-0.339; β_HGS HIV+_: -0.465;***  ***β_BP HIV+_: -0.001; β_LPD HIV-_:***  ***-0.005; β_LP HIV+_: -0.012)***  ***Coriobacteriaceae (β_CST HIV+_:***  ***-0.712; β_HGS HIV-_: -0.414; β_LP HIV+_:***  ***-0.042; β LP HIV-: -0.018)***  ***Rikenellaceae (β_CST HIV-_:***  ***-0.133; β_HGS HIV-_: -0.736; β_BP HIV+_:***  ***-0.006; β_BP HIV-_: -0.009; β_LPD HIV+_:***  ***-0.017; β_LPD HIV-_: -0.002)***  ***Erysipelotrichaceae (β_CST HIV+_:***  ***-0.756; β_CST HIV-_: -0.535; β_HGS HIV+_:***  ***-0.704; β_LPD HIV-_: 0.004)***  ***Enterobacteriaceae (β_BP HIV-_:***  ***-0.008; β_LPD HIV+_: -0.002; β_HGS HIV-_:***  ***-0.808)***  **Genus: *Bifidobacterium (β _HGS HIV+_:***  ***-0.189; β _HGS HIV-_: -0.527;***  ***β _LP HIV+_: -0.0241; -0.122)***  ***Collinsella (β_HGS HIV+_: -0.610;***  ***β_HGS HIV-_: -0.507; β_LPD HIV+_:***  ***-0.019; β_LP HIV+_: -0.049; β_LP HIV-_:***  ***-0.043)***  ***Alistipes (β_HGS HIV-_: -0.389;***  ***β_BP HIV+_: -0.019; β_BP HIV-_: -0.011;***  ***β_LPD HIV+_: -0.017; β_LPD HIV-_: -0.002)***  ***Bacteroides (β_CST HIV-_: -0.036;***  ***β _HGS HIV-_: -0.119; _HGS HIV+_:***  ***-0.175; β _BP HIV+_: -0.003)***  ***Parabacteroies (β _HGS HIV+_:***  ***-0.242; β_BP HIV+_: -0.006; β_LBP HIV+_:***  ***-0.001)***  ***Prevotella (β_HGS HIV+_: - 0.061;***  ***β_HGS HIV-_: -0.092; β_LBP HIV-_: -0.001)***  ***Anaerostipes (β _CST HIV+_: -0.36;***  ***β _CST HIV-_: -0.23; β_HGS HIV+_: -0.44;***  ***β_HGS HIV-_: -0.92; β_BP HIV+_: - 0.02)***  ***Anaerotruncus (β_CST HIV+_:***  ***-0.158; β_BP HIV-_: -0.049; β_LPD HIV-_:***  ***-0.085; β _LP HIV-_: -0.032)***  ***Blautia (β_CST HIV+_: -0.324;***  ***β_HGS HIV+_: -0.414; β_HGS HIV-_:***  ***-0.808; β_BP HIV-_: -0.002; β_LP HIV+_:***  ***-0.043; β_LP HIV-_: -0.032)***  ***Catenibacterium (β_CST HIV+_:***  ***-0.915; β_CST HIV-_: -0.861;***  ***β_HGS HIV+_: -0.696; β_LPD HIV-_:***  ***-0.018)***  ***Coprococcus (β_CST HIV+_: -0.117;***  ***β_HGS HIV+_: -3.32; β_BP HIV-_: -0.010;***  ***β_LPD HIV-_: -0.013; β_LP HIV+_: -0.003)***  ***DIalister (β_CST HIV-_: 0.699;***  ***Β_CST HIV+_: -0.224; β_HGS HIV+_:***  ***-0.110;***  ***β_BP HIV+_: -0.007; β_LP HIV+_: -0.083)***  ***Dorea (β_CST HIV-_: -0.619; β_HGS HIV+_:***  ***-0.883; β_BP HIV-_: -0.030; β_LPD HIV-_:***  ***-0.012; β*** ***LP HIV+: -0.068)***  ***Faecalibacterium (β_HGS HIV+_:***  ***-0.164; β_BP HIV+_: -0.023; β_LPD HIV+_:***  ***-0.029; β_LP HIV+_: -0.007; β_LP HIV-_:***  ***-0.040; β_LPD HIV-_: -0.013; β_BP HIV-_:***  ***-0.003)***  ***Megamonas (β_HGS HIV+_: -0.767;***  ***β_HGS HIV-_: -0.297); β_HGS HIV+_: -2.10;***  ***β_HGS HIV-_: -0.258; β_BP HIV-_: -0.002;***  ***β_LPD HIV-_:-0.011)***  ***Megasphaera (β_CST HIV-_: -0.751;***  ***β_HGS HIV+_: -0.381; β_BP HIV-_: -0.001)***  ***Phascolarctobacterium***  ***(β_CST HIV+_: -4.08; β_CST HIV-_: -0.257;***  ***β_LP HIV+_: -0.102; β_LP HIV-_: -0.076)***  ***Roseburia (β_CST HIV+_: -0.589;***  ***β_HGS HIV+_: -0.232; β_LP HIV+_: -0.141;***  ***β_LP HIV-_: -0.016)***  ***Pseudobutyrivibrio (β_CST HIV+_:***  ***-0.242; β_HGS HIV-_: -0.069; β_BP HIV+_:***  ***-0.031; β_BP HIV-_: -0.004; β_LP HIV+_:***  ***-0.048)***  ***Ruminococcus (β_HGS HIV+_: -0.219;***  ***β_LP HIV+_: -0.120)***  ***Subdoligranulum (β_HGS HIV+_:***  ***-0.351; β_BP HIV+_: -0.013; β_LPD HIV+_:***  ***-0.006; β_LP HIV+_: -0.188)***  ***Fusobacterium (β_CST HIV+_:***  ***-0.227; β_CST HIV-_: -0.402;***  ***β_HGS HIV+_:-0.059; β _HGS HIV-_:***  ***-0.477; β_BP HIV+_: -0.005; β_BP HIV-_:***  ***0.003; β_LPD HIV+_: -0.011;***  ***β_LPD HIV-_: -0.017; β_LP HIV+_: -0.008;***  ***β_LP HIV-_: -0.025***  ***Escherichia (β_CST HIV+:_ -0.709;***  ***β_CST HIV-_: -0.834; β_BP HIV+_: -0.215;***  ***β_BP HIV+_: -0.009; β_LPD HIV+_:***  ***-0.083)***  ***Succinovibrio (β_CST HIV-_: -0.566;***  ***β_HGS HIV+_: -0.292; β_BP HIV+_:***  ***-0.073; β_LPD HIV-_: -0.007;***  ***β_LPD HIV+_: -0.022; β_LP HIV-_: -0.067)***  ***Sutterella (β_CST HIV-_: -1.26;***  ***β_LP HIV-_: -0.026)***  ***Butyrivibrio (β_CST HIV+_: -16.66;***  ***β_CST HIV-_: -3.72; β_LPD HIV-_: -0.07;***  ***β_LP HIV-_ : -0.05)*** | **α-diversity**  NA  **β-diversity**  NA | Not adjusted |
| Lim et al., South-Korea, 2020 (34) | N = 176,  HGS | **Multiple associations with bacteria at genus and species level were assessed but no exact coefficients or p-values were derived.** | **Multiple associations with bacteria at genus and species level were assessed but no exact coefficients or p-values were derived.**  **:** | **α-diversity**  **Shannon’s diversity (β: -0.013), Pielou’s evenness (β: - 0.001); Observed ASVs (β: -1.174), Faith’ Phylogenetic Diversity (β: -0.088)**  **β-diversity**  **NA** | Age, sex |
| Houttu et al., The Netherlands, 2021 (31) | N = 1334,  HGS | **Species*: Bacteroides clarus***  ***Bacteroides caccae***  ***Bacteroides ovatus***  ***Bacteroides eggerthii***  ***Bacteroides plebeius***  ***Alistipes putredinis***  ***Parabacteroides distasionis***  ***Prevotella 9 copri***  ***Bacteroides coprophilus***  ***Roseburia unulinivorans***  ***Roseburia hominis***  ***Desulfovibrio piger***  ***Collinsella aerofaciens***  ***Faecalibacterium***  ***prausnitzii***  **Genus: *Roseburia***  ***Agathobacter***  ***Desulfovibrio***  ***Slackia***  ***Collinsella***  ***Phascorarctobacterium***  ***Ruminococcus 2***  ***Ruminiclostridium***  **Family: *Marinifilaceae***  ***Rikenellaceae***  ***Barnesiellaceae***  ***Muribaculaceae***  ***Prevotellaceae***  ***Tannerellaceae***  ***Ruminococcaceae***  ***Lentisphaerae***  ***Akkermansiaceae***  ***Desulfovibrionaceaea***  ***Peptococcaceae***  **Phylum:*Verrucomicrobia***  ***Firmicutes***  ***Tenericutes***  ***Proteobacteria*** | **Species*: Bacteroides uniformis***  ***Bacteroides intestinalis***  ***Odoribacter splanchnicus***  ***Alistipes shahii***  ***Alistipes finegoldii***  ***Barnesiella intestinihominis***  ***Bacteroides massiliensis***  ***Parabacteroides merdae***  ***Bifidobacterium bifidum***  **Genus: *Fusicatenibacter***  ***Succinivibrio***  ***Haemophilus***  ***Klebsiella***  ***Bifidobacterium***  ***Megamonas***  ***Catenibacterium***  ***Flavonifractor***  **Family*: Bacteroidaceae***  ***Lachnospiraceae***  ***Succinivirbionaceae***  ***Lactobacillaceae***  **Phylum: *Bacteroidetes***  ***Actinobacteria*** | **α-diversity**  **Shannon index (β: 0.00), Faith’s phylogenetic diversity index (β: 0.005), Simpson index (β: 0.00)**  **β-diversity**  NA | Age, sex, BMI, ethnicity, average fat -, grain and carbohydrate intake |
| *Ponziani et al., Italy, 2021* | ***N = 100***  ***HGS*** | **Family *: Prevotellaceae***  ***(r :0.230)***  ***Porphyromonadaceae***  ***(r : 0.1394)***  ***Ruminococcaceae***  ***(r : 0.079)***  ***Barnesiellaceae***  ***(r : 0.008)***  **Genus *: Prevotella***  ***(r : 0.2309)***  ***Slackia (r : 0.19)***  ***Methanobrevibacter***  ***(r : 0.144)***  ***Ruminococcus***  ***(r : 0.121)***  ***Lachnobacterium***  ***(r : 0.099)***  ***Atopobium (r : 0.08)***  ***Christenella (r : 0.04)*** | **Family: *Veillonellaceae (r: -0.109)***  ***Rikenellaceae (r: -0.0841)***  ***Desulfovibrionaceae***  ***(r: -0.00978)***  ***Verrucomicrobiaceae***  ***(r: -0.0023)***  **Genus: *Eggerthella (r: -0.251)***  ***Dialister (r: -0.230)***  ***Streptococcus (r: -0.101)***  ***Klebsiella (r: -0.084)***  ***Akkermansia (r: -0.0027)*** |  |  |
| Kang et al., China, 2021 (14) | N =87,  CST time, HGS | **Genus: *Blautia (r_HGS_)***  ***Megasphaera***  ***(r_HGS&CST_)***  ***Megamonas***  ***(r_HGS&CST_)***  ***Alistipes(r_HGS&CST_)***  ***Fusicatenibacter(r_HGS&CST_)***  ***Phascolarctobacterium(r_HGS_)***  ***Bifidobacterium(r_HGS&CST_)***  ***Ruminococcus(r_HGS&CST_)***  ***Lactobacillus(r_HGS&CST_)***  ***Roseburia(r_HGS_)***  ***Subdoligranulum(r_HGS_)***  ***Escherichia/Shigella(r_CST_)***  ***Bacteroides (r_CST_)***  ***Parabacteroides (r_CST_ )*** | **Genus: *Parabacteroides(r_HGS_)***  ***Bacteroides(r_HGS_)***  ***Escherichia/***  ***Shigella(r_HGS_)***  ***Lachnoclostridium (r_HGS&CST_)***  ***Blautia (r_CST_)***  ***Faecalibacterium(r_CST_)***  ***Prevotella 9(r_CST_)***  ***Fusicatenibacter(r_CST_)***  ***Phascolarctobacterium(r_CST_)*** | **α-diversity**  NA  **β-diversity**  NA | Not adjusted |
| Han et al., Taiwan, 2022 (13) | N = 88;  HGS | **Species*: Family XII***  ***AD3001 group sp.***  ***Family XII UCG***  ***001 sp.***  ***Christensenellaceae***  ***R-Z group sp.***  ***Eubacterium***  ***coprostanoligenes***  ***group sp.***  ***Ruminococcaceae***  ***NK4A214 group sp.***  ***Parabacteroides***  ***johnsonii***  ***CL02T12C29*** | **Genus: *Marvinbryantia***  ***Fusobacterium sp.***  ***Streptococcus sp.***  ***Leuconostroc sp.***  ***Sellimonas sp.***  ***Favonifractor sp.***  ***Enterococcus sp.***  **Species: *Ruminococcaceae***  ***UCG-010 sp.***  ***Ruminococcus gnavus group***  ***sp.***  ***Ruminiclostridium 5 sp.***  ***Clostridium innocuum group***  ***sp.*** | **α-diversity**  NA  **β-diversity**  NA | Age, BMI, MNA-score, physical activity level |
| Hu et al., China, 2022 (32) | N = 102,  HGS | **No associations with other bacterial taxa were specified** | Genus: ***Escherichia (r_PD+_ : -0.212)*** | **α-diversity**  **No non-significant correlations specified**  **β-diversity**  NA | Not adjusted |
| Yan et al., China, 2023 (42) | N = 276,  HGS | Phylum: ***Desulfobacteriota***  ***Bacteroidota***  ***Firmicutes***  Genus: ***Bacteroides***  ***Dorea***  ***Ruminococcus***  ***Eubacterium hallii***  ***group***  ***Faecalibacterium***  ***Shigella***  ***Blautia*** | Phylum: ***Verrucomicrobiota***  Genus: ***Clostridia***  ***Clostridium sensu stricto 1***  ***Collinsella***  ***Romboutsia***  ***Enterobacter*** | **α-diversity**  ***F/B ratio (-)***  **β-diversity**  NA | Age, BMI, waistline, ALT, ALT/AST, total bilirubin |
| Davis et al., Australia, 2021 | N = 490  HGS | **No non-significant associations specified** | **No non-significant associations specified** | **α-diversity**  **Shannon index (β: 0.803), Observed species (β: 0.001)**  **β-diversity**  **Unweighted UniFrac distance Weighted UniFrac distance** | **Age, smoking, physical activity, intestinal symptoms, batch effects, medications** |

## **Table S4: Associations between GM taxa and physical performance: non-significant findings**

| Author, country, year | Sample size (N),  Physical performance estimate | GM taxa | | Diversity markers | Adjustment for confounders |
| --- | --- | --- | --- | --- | --- |
|  |  | **Positive association** | **Negative association** |  |  |
| Dillon et al., USA, 2020 (47) | N = 36,  SPPB, 400 m walk test, stair climb time | **Phylum*: Firmicutes (β_SPPB HIV+_:***  ***0.0165; β_walk time_ _HIV-_:***  ***0.3142)***  ***Bacteroidetes***  ***(β_SPPB HIV-_: 0.0062***  ***; β_stair climb HIV+_: 0.0116)***  ***Proteobacteria***  ***(β_SPPB HIV-_: 0.0274;***  ***β_stair climb HIV-_: 0.0211;***  ***β_walk time HIV-_: 1.015)***  ***Actinobacteria***  ***(β _SPPB HIV+_: 0.0903;***  ***β_stair climb HIV-_: 0.0571;***  ***β_walk time HIV-_: 1.693)***  ***Fusobacterium***  ***(β_SPPB HIV+_: 0.0316;***  ***β SPPB HIV-: 0.0358)***  **Family*: Lachnospiraceae***  ***(β_SPPB HIV+_: 0.0521;***  ***β_stair climb HIV-_:***  ***0.0039; β_walk time HIV-_:***  ***0.2531)***  ***Ruminococcaceae***  ***(β_SPPB HIV+_: 0.0228;***  ***β_walk time HIV-_: 0.2949)***  ***Bacteroidaceae***  ***(β_walk time HIV+_: 0.7399;***  ***β_stair climb HIV-_: 0.0007***  ***Prevotellaceae***  ***(β_SPPB HIV+_: 0.0140;***  ***β_SPPB HIV-_: 0.0089;***  ***β_stair climb HIV+_: 0.0237;***  ***β_walk time HIV+_: 0.2351)***  ***Porphyromonadaceae***  ***(β_SPPB HIV-_: 0.0003;***  ***β_walk time HIV+_: 1.523)***  ***Veillonellaceae***  ***(β_SPPB HIV-_: 0.0220)***  ***Coriobacteriaceae***  ***(β_SPPB HIV+_: 0.0967;***  ***β_stair climb HIV-_: 0.073;***  ***β_walk time HIV-_: 1.875)***  ***Rikenellaceae (β_SPPB HIV-_:***  ***0.0271; β_walk time HIV+_:***  ***2.713)***  ***Erysipelotrichaceae***  ***(β_SPPB HIV+_: 0.0510;***  ***β_SPPB HIV-_: 0.0622)***  ***Enterobacteriaceae***  ***(β _SPPB HIV-_: 0.0228;***  ***β_stair climb HIV-_: 0.0197;***  ***β_walk time HIV-_: 2.604;***  ***β_walk time_ HIV+: 1.542)***  **Genus*: Bifidobacterium***  ***(β_stair climb HIV+_: 0.175)***  ***Collinsella (β_SPPB HIV+_:***  ***0.095; β_stair climb HIV-_:***  ***0.105; β_walk time HIV-_:***  ***3.283)***  ***Alistipes (β_SPPB HIV-_:***  ***0.024; β_walk time HIV+_:***  ***4.621)***  ***Bacteroides***  ***(β_walk time HIV+_:***  ***0.740; β_stair climb HIV-_:***  ***0.001)***  ***Parabacteroides***  ***(β_walk time HIV+_: 1.507;***  ***β_stair climb HIV-_: 0.004)***  ***Prevotella (β _SPPB HIV+_:***  ***0.014; β_walk time HIV+_:***  ***0.287; β_SPPB HIV-_: 0.009)***  ***Anaerostipes (β_SPPB HIV+_:***  ***0.03; β_walk time HIV+_: 4.14;***  ***β_stair climb HIV-_: 0.01)***  ***Blautia (β_SPPB HIV+_: 0.009;***  ***β_stair climb HIV-_: 0.056;***  ***β_walk time HIV-_: 1.016)***  ***Catenibacterium***  ***(β_SPPB HIV+_: 0.057;***  ***β_SPPB HIV-_: 0.113;***  ***β_walk time HIV-_: 0.539)***  ***Coprococcus***  ***(β_walk time HIV+_: 4.642;***  ***β_walk time HIV-_: 1.498)***  ***Dialister (β_SPPB HIV+_:***  ***0.061; β_SPPB HIV-_: 0.055;***  ***β_stair climb HIV+_: 0.033)***  ***Dorea (β _SPPB HIV-_: 0.184)***  ***Faecalibacterium***  ***(β_stair climb HIV+_: 0.139;***  ***β_stair climb HIV-_: 0.012;***  ***β_walk time HIV-_: 1.275)***  ***Megamonas (β_SPPB HIV+_:***  ***0.114; β_SPPB HIV-_: 0.029;***  ***β_stair climb HIV+_: 0.163;***  ***β_walk time HIV+_: 0.497)***  ***Megasphaera***  ***(β_walk time HIV+_: 0.805;***  ***β_SPPB HIV-_: 0.006)***  ***Phascolarctobacterium***  ***(β_stair climb HIV+_: 0.378;***  ***β_SPPB HIV-_: 0.049)***  ***Pseudobutyrivibrio***  ***(β_SPPB HIV+_: 0.006;***  ***β_walk time HIV-_: 1.024)***  ***Roseburia (β_SPPB HIV+_:***  ***0.161; β_SPPB HIV-_: 0.002;***  ***β_stair climb HIV+_: 0.399)***  ***Ruminococcus (_β SPPB HIV-_:***  ***0.012; β_walk time HIV-_:***  ***0.183)***  ***Fusobacterium***  ***(β_SPPB HIV+/-_ : 0.036)***  ***Escherichia (β_SPPB HIV-_:***  ***0.023; β_walk time HIV+_:***  ***53.24; β_SPPB HIV-_: .023;***  ***β_stair climb HIV-_: 0.020;***  ***β_walk time HIV-:_ 2.683)***  ***Succinivibrio (β_SPPB HIV-_:***  ***0.057; β_stair climb HIV-_:***  ***0.065)***  ***Sutterella (β_SPPB HIV-_:***  ***0.006; β_stair climb HIV+_:***  ***0.167;***  ***β_walk time HIV+_: 2.527)***  ***Butyrivibrio (β_SPPB HIV+_:***  ***0.72; β_SPPB HIV-_: 0.27)*** | **Phylum:** ***Firmicutes (β_SPPB HIV-_:***  ***-0.005; β_stair climb HIV+_:***  ***-0.019; β_walk time HIV+_:***  ***-0.816; β _stair climb HIV-_:***  ***-0.003)***  ***Bacteroidetes (β_SPPB HIV+_:***  ***-0.007; β_stair climb HIV-_:***  ***-0.006; β_walk time HIV-_:***  ***-0.597)***  ***Actinobacteria***  ***(β_stair climb HIV+_: -0.032;***  ***β_walk time HIV+_: -1.96;***  ***β_SPPB HIV-_: 0.024)***  ***Fusobacterium***  ***(β_stair climb HIV+_: -0.034;***  ***β_walk time HIV+_: -0.967;***  ***β_stair climb HIV-_: -0.039;***  ***β_walk time HIV-_: -0.698)***  **Family: *Lachnospiraceae***  *(****β_SPPB HIV-_: -0.005;***  ***β_stair climb HIV+_: -0.057;***  ***β_walk time HIV+_: -1.32)***  ***Ruminococcaceae***  ***(β_SPPB HIV-_: -0.013;***  ***β_stair climb HIV-_: -0.016;***  ***β_stair climb HIV+_: -0.041;***  ***β_walk time HIV+_: -1.17)***  ***Bacteroidaceae (β_SPPB HIV-_:***  ***-0.001; β_walk time HIV-_:***  ***-0.345; β_stair climb HIV+_:***  ***-0.005)***  ***Prevotellaceae***  ***(β_stair climb HIV-_: -0.004;***  ***β_walk time HIV-_: -0.201;***  ***β_stair climb HIV+_: -0.016)***  ***Porphyromonadaceae***  ***(β_SPPB HIV+_: -0.013;***  ***β_stair climb HIV+_: -0.016;***  ***β_stair climb HIV-_: -0.008;***  ***β_walk time HIV-_: -0.715)***  ***Veillonellaceae***  ***(β_SPPB HIV+_: -0.013;***  ***β_walk time HIV+_: -0.439;***  ***β_stair climb HIV-_: -0.025;***  ***β_walk time HIV-_: -0.135)***  ***Coriobacteriaceae***  ***(β_SPPB HIV-_: - 0.032;***  ***β_stair climb HIV+_: -0.045;***  ***β_walk time HIV+_: -0.185)***  ***Rikenellaceae (β_SPPB HIV+_:***  ***-0.023; β_stair climb HIV+_:***  ***-0.218; β_stair climb HIV-_:***  ***-0.058; β_walk time HIV-_:***  ***-0.679)***  ***Erysipelotrichaceae***  ***(β_stair climb HIV+_: -0.058;***  ***β_walk time HIV+_: -2.74;***  ***β _stair climb HIV-_: -0.052;***  ***β_walk time HIV-_: -1.83)***  **Genus: *Bifidobacterium***  ***(β_SPPB HIV+_: -0.034;***  ***β _SPPB HIV-_: -0.070;***  ***β_walk time HIV+_: -2.85)***  ***Collinsella (β_SPPB HIV-_:***  ***-0.042; β_stair climb HIV+_:***  ***-0.036; β_walk time HIV+_:***  ***-1.64)***  ***Alistipes (β_SPPB HIV+_: -0.241;***  ***β_stair climb HIV+_: -0.049;***  ***β_stair climb HIV-_: -0.059;***  ***β_walk time HIV-_: -0.541)***  ***Bacteroides (β_SPPB HIV-_:***  ***-0.001; β_walk time HIV-_:***  ***-0.345; β_stair climb HIV+_:***  ***-0.005)***  ***Parabacteroides***  ***(β_SPPB HIV+_: - 0.014;***  ***β_SPPB HIV-_: -0.007;***  ***β_walk time HIV-_: -0.213;***  ***β_stair climb HIV+_: -0.015)***  ***Prevotella (β_stair climb HIV-_:***  ***-0.005; β_walk time HIV-_:***  ***-0.204)***  ***Anaerostipes***  ***(β_stair climb HIV+_: -0.03;***  ***β_walk time HIV -_: -1.05)***  ***Anaerotruncus (β_SPPB HIV+_:***  ***-0.138; β_SPPB HIV-_: -0.231;***  ***β_stair climb HIV-_: -0.020;***  ***β_walk time HIV-_: -1.19;***  ***β_stair climb HIV+_: -0.377;***  ***β_walk time HIV+_: -5.33)***  ***Blautia (β _SPPB HIV-_: -0.039;***  ***β_stair climb HIV+_: -0.102;***  ***β_walk time HIV+_: -1.01)***  ***Catenibacterium***  ***(β_stair climb HIV+_: -0.043;***  ***β_walk time HIV+_: -0.281;***  ***β_stair climb HIV-_: -0.034)***  ***Coprococcus (β_SPPB HIV+_:***  ***-0.055; β _SPPB HIV-_:***  ***-0.010; β_stair climb HIV+_:***  ***-0.241; β_stair climb HIV-_:***  ***-0.138)***  ***Dialister (β_walk time HIV+_:***  ***-0.266; β_stair climb HIV-_:***  ***-0.086; β_walk time HIV-_: -2.07)***  ***Dorea (β_stair climb HIV+_:***  ***-0.171; β_walk time HIV+_: -7.94;***  ***β_stair climb HIV-_: -0.253;***  ***β_walk time HIV-_: -1.38)***  ***Faecalibacterium***  ***(β_SPPB HIV+_: -0.082; β_SPPB HIV-_:***  ***-0.017; β_walk time HIV+_:-0.885)***  ***Megamonas (β_stair climb HIV-_:***  ***-0.038; β_walk time HIV-_: -0.443)***  ***Megasphaera (β_SPPB HIV+_:***  ***-0.110; β_stair climb HIV-_:***  ***-0.054; β_walk time HIV-_: - 1.07)***  ***Phascolarctobacterium***  ***(β_walk time HIV+_: -2.37;***  ***β_stair climb HIV-_: -0.073;***  ***β_walk time HIV-_: 0.310)***  ***Pseudobutyrivibrio***  ***(β_stair climb HIV+_: -0.098;***  ***β_walk time HIV+_: -4.66;***  ***β_SPPB HIV-_: -0.002;***  ***β_stair climb HIV-_: -0.003)***  ***Roseburia (β_stair climb HIV-_:***  ***-0.027; β_walk time HIV-_:***  ***-2.42; β_walk time HIV+_: -2.07)***  ***Ruminococcus (β_SPPB HIV+_:***  ***-0.310; β_stair climb HIV+_:***  ***-0.156; β_walk time HIV+_: 0.699;***  ***β_stair climb HIV-_:-0.096)***  ***Subdoligranulum***  ***(β_SPPB HIV+_: -0.233;***  ***β_SPPB HIV-_: -0.081;***  ***β_stair climb HIV+_: -0.175;***  ***β_stair climb HIV-_: -0.009;***  ***β_walk time HIV+_: -10.7;***  ***β_walk time HIV-_: -2.26)***  ***Fusobacterium***  ***(β_stair climb HIV+_: -0.038;***  ***β_stair climb HIV-_: -0.039;***  ***β_walk time HIV+_: -1.09;***  ***β_walk time HIV-_: -0.714)***  ***Escherichia***  ***(β_stair climb HIV+_: -0.156)***  ***Succinivibrio***  ***(β_SPPB HIV+_: -0.062;***  ***β_stair climb HIV+_: -0.214;***  ***β_walk time HIV+_: -0.947;***  ***β_walk time HIV-_: -2.95)***  ***Butyrivibrio (β_stair climb HIV+_:***  ***-1.82; β_stair climb HIV-_: -0.01;***  ***β_walk time HIV+_: -65.28;***  ***β_walk time HIV-_ : 13.37)*** | **α-diversity**  NA  **β-diversity**  NA | Not adjusted |
| Davis et al., Australia, 2021 (44) | N = 490,  TUG test | **No non-significant associations specified** | **No non-significant associations specified** | **α-diversity**  **Shannon index (β: -0.133), Observed species (β: 0.001)**  **β-diversity**  **Unweighted UniFrac distance p:0.253, Weighted UniFrac distance p: 0.266)** | Age, smoking, physical activity, intestinal symptoms, medications, batch effects, fat mass, Australian Recommended Food Score |
| Han et al., Taiwan, 2022 (13) | N = 88,  Gait speed | **Species: *Bacteroides eggerthii***  ***DSM 20697***  ***Bifidobacterium***  ***longum***  ***Ruminococcaceae***  ***NK4A214 group sp.*** | **Genus: *Akkermansia sp.***  ***Sellimonas sp.***  ***Enterococcus sp.***  ***Ruminiclostridium 5 sp.***  ***Leuconostoc sp.***  **Species: *Ruminococcaceae***  ***UCG-010 sp.***  ***Chistensenellaceae R-7***  ***group sp.***  ***Family XII UCG-001 sp.***  ***Family XII AD30111***  ***Ruminococcus gnavus***  ***Clostridium innocuum***  ***group sp.***  ***Lactobacillus salivarius*** | **α-diversity**  NA  **β-diversity**  NA | Age, BMI, MNA-score, physical activity |
| Yan et al., China, 2023 | N = 276  Gait speed | **Phylum: Desulfobacterota**  **Firmicutes**  **Genus: *Clostridia***  ***Fusicatenibacter***  ***Agathobacter***  ***Faecalibacterium***  ***Blautia*** | **Phylum: *Verrucomicrobiota***  ***Actinobacteriota***  ***Proteobacteria***  **Genus: *Ruminococcus***  ***torques group***  ***UCG014***  ***Clostridium***  ***sensu stricto1***  ***Dorea***  ***Ruminococcus***  ***Collinsella***  ***Bifidobacterium***  ***Shigella*** | F/B ratio (r+) |  |

| **Section and Topic** | **Item #** | **Checklist item** | **Location where item**  **is reported** |
| --- | --- | --- | --- |
| **TITLE** | | |  |
| Title | 1 | Identify the report as a systematic review. | Page 1 |
| **ABSTRACT** | | |  |
| Abstract | 2 | See the PRISMA 2020 for Abstracts checklist. | Page 2 |
| **INTRODUCTION** | | |  |
| Rationale | 3 | Describe the rationale for the review in the context of existing knowledge. | Page 3-4 |
| Objectives | 4 | Provide an explicit statement of the objective(s) or question(s) the review addresses. | Page 4-5 |
| **METHODS** | | |  |
| Eligibility criteria | 5 | Specify the inclusion and exclusion criteria for the review and how studies were grouped for the syntheses. | Page 4-5 |
| Information sources | 6 | Specify all databases, registers, websites, organisations, reference lists and other sources searched or consulted to identify studies. Specify the date when each source was last searched or consulted. | Page 4-5 |
| Search strategy | 7 | Present the full search strategies for all databases, registers and websites, including any filters and limits used. | Page 4 |
| Selection process | 8 | Specify the methods used to decide whether a study met the inclusion criteria of the review, including how many reviewers screened each record and each report retrieved, whether they worked independently, and if applicable, details of automation tools used in the process. | Supplementary materials, page 2-6 (Appendix I) |
| Data collection process | 9 | Specify the methods used to collect data from reports, including how many reviewers collected data from each report, whether they worked independently, any processes for obtaining or confirming data from study investigators, and if applicable, details of automation tools used in the process. | Page 4 |
| Data items | 10a | List and define all outcomes for which data were sought. Specify whether all results that were compatible with each outcome domain in each study were sought (e.g. for all measures, time points, analyses), and if not, the methods used to decide which results to collect. | Page 5 |
|  | 10b | List and define all other variables for which data were sought (e.g. participant and intervention characteristics, funding sources). Describe any assumptions made about any missing or unclear information. | Page 5 |
| Study risk of bias assessment | 11 | Specify the methods used to assess risk of bias in the included studies, including details of the tool(s) used, how many reviewers assessed each study and whether they worked independently, and if applicable, details of automation tools used in the process. | Page 4-5 |
| Effect measures | 12 | Specify for each outcome the effect measure(s) (e.g. risk ratio, mean difference) used in the synthesis or presentation of results. | NA |
| Synthesis methods | 13a | Describe the processes used to decide which studies were eligible for each synthesis (e.g. tabulating the study intervention characteristics and comparing against the planned groups for each synthesis (item #5)). | Page 5 |
|  | 13b | Describe any methods required to prepare the data for presentation or synthesis, such as handling of missing summary statistics, or data conversions. | NA |
|  | 13c | Describe any methods used to tabulate or visually display results of individual studies and syntheses. | NA |
|  | 13d | Describe any methods used to synthesize results and provide a rationale for the choice(s). If meta-analysis was performed, describe the model(s), method(s) to identify the presence and extent of statistical heterogeneity, and software package(s) used. | NA |
|  | 13e | Describe any methods used to explore possible causes of heterogeneity among study results (e.g. subgroup analysis, meta-regression). | NA |
|  | 13f | Describe any sensitivity analyses conducted to assess robustness of the synthesized results. | NA |
| Reporting bias assessment | 14 | Describe any methods used to assess risk of bias due to missing results in a synthesis (arising from reporting biases). | NA |
| Certainty assessment | 15 | Describe any methods used to assess certainty (or confidence) in the body of evidence for an outcome. | NA |

| **Section and Topic** | **Item #** | **Checklist item** | **Location where item**  **is reported** |
| --- | --- | --- | --- |
| **RESULTS** | | |  |
| Study selection | 16a | Describe the results of the search and selection process, from the number of records identified in the search to the number of studies included in the review, ideally using a flow diagram. | Page 6-8 |
|  | 16b | Cite studies that might appear to meet the inclusion criteria, but which were excluded, and explain why they were excluded. | Page 8: Reasons for exclusion in PRISMA flow diagram. |
| Study characteristics | 17 | Cite each included study and present its characteristics. | Supplementary materials, Appendix II, page 7-12 |
| Risk of bias in studies | 18 | Present assessments of risk of bias for each included study. | Page 7  Supplementary materials Appendix VII, page 20 |
| Results of individual studies | 19 | For all outcomes, present, for each study: (a) summary statistics for each group (where appropriate) and (b) an effect estimate and its precision (e.g. confidence/credible interval), ideally using structured tables or plots. | Page 12-13; page 17-20; page |
| Results of syntheses | 20a | For each synthesis, briefly summarise the characteristics and risk of bias among contributing studies. | Supplementary materials, Appendix II, page 7-12; Appendix VII, page 20. |
|  | 20b | Present results of all statistical syntheses conducted. If meta-analysis was done, present for each the summary estimate and its precision (e.g. confidence/credible interval) and measures of statistical heterogeneity. If comparing groups, describe the direction of the effect. | Page 9-24 |
|  | 20c | Present results of all investigations of possible causes of heterogeneity among study results. | NA |
|  | 20d | Present results of all sensitivity analyses conducted to assess the robustness of the synthesized results. | NA |
| Reporting biases | 21 | Present assessments of risk of bias due to missing results (arising from reporting biases) for each synthesis assessed. | NA |
| Certainty of evidence | 22 | Present assessments of certainty (or confidence) in the body of evidence for each outcome assessed. | NA |
| **DISCUSSION** | | |  |
| Discussion | 23a | Provide a general interpretation of the results in the context of other evidence. | Page 29-32 |
|  | 23b | Discuss any limitations of the evidence included in the review. | Page 31-32 |
|  | 23c | Discuss any limitations of the review processes used. | Page 29-32 |
|  | 23d | Discuss implications of the results for practice, policy, and future research. | Page 31-32 |
| **OTHER INFORMATION** | | |  |
| Registration and protocol | 24a | Provide registration information for the review, including register name and registration number, or state that the review was not registered. | Page 4 |
|  | 24b | Indicate where the review protocol can be accessed, or state that a protocol was not prepared. | Page 4 |
|  | 24c | Describe and explain any amendments to information provided at registration or in the protocol. | NA |
| Support | 25 | Describe sources of financial or non-financial support for the review, and the role of the funders or sponsors in the review. | NA |
| Competing interests | 26 | Declare any competing interests of review authors. | NA |
| Availability of data, code and other materials | 27 | Report which of the following are publicly available and where they can be found: template data collection forms; data extracted from included studies; data used for all analyses; analytic code; any other materials used in the review. | NA |

*From:* Page MJ, McKenzie JE, Bossuyt PM, Boutron I, Hoffmann TC, Mulrow CD, et al. The PRISMA 2020 statement: an updated guideline for reporting systematic reviews. BMJ 2021;372:n71. doi: 10.1136/bmj.n71

For more information, visit: <http://www.prisma-statement.org/>
